# Multifunctional Photocatalysts Based on Half-Sandwich Cyclometalated Ir(III) Complexes with π‑Extended Ligands

**DOI:** 10.1021/acs.inorgchem.6c00584

**Published:** 2026-04-03

**Authors:** Carlos Gonzalo-Navarro, Melania Sánchez, Gregorio Castañeda, Pedro Tavares, Blanca R. Manzano, Gema Durá

**Affiliations:** 1 Departamento de Q. Inorgánica, Orgánica y Bioquímica, Facultad de Ciencias y Tecnologías Químicas, 16733 Universidad de Castilla-La Mancha, Avda. Camilo José Cela, 10, Ciudad Real 13071, Spain; 2 Instituto Regional de Investigación Científica Aplicada (IRICA), UCLM, Avda. Camilo José Cela, Ciudad Real 13071, Spain; 3 Departamento de Q. Analítica y Tecnología de los Alimentos, Facultad de Ciencias y Tecnologías Químicas, 16733 Universidad de Castilla-La Mancha, Avda. Camilo José Cela, 10, Ciudad Real 13071, Spain; 4 Associate Laboratory i4HB - Institute for Health and Bioeconomy, NOVA School of Science and Technology, Universidade NOVA de Lisboa, Caparica 2829-516, Portugal; 5 UCIBIO − Applied Molecular Biosciences Unit, Department of Chemistry, NOVA School of Science and Technology, Universidade NOVA de Lisboa, Caparica 2829-516, Portugal

## Abstract

Photocatalysis has emerged as a powerful strategy in synthetic organic chemistry to avoid harsh conditions and toxic oxidants. Herein, we report the photocatalytic activity of half-sandwich Ir­(III) complexes in different transformations. For the first time, Cp*Ir­(III) complexes were applied in photooxidative coupling reactions of amines, without any additives, achieving quantitative imine formation, and in thioether photooxidation reactions, reaching high chemoselectivity for sulfoxide formation. Furthermore, exceptional activity was observed in photodehydrogenation of N-heterocyclic substrates. Reactions proceeded under mild conditions, including room temperature and an ambient air or oxygen atmosphere, with considerably low catalyst loadings (0.005–0.25 mol %). Complex **1**, bearing the π-extended C^∧^N pbpn ligand, was also active even in aqueous media (MeCN/H_2_O 1:1) and under sunlight, reaching full conversion within hours. Mechanistic studies indicated the involvement of reactive oxygen species, such as singlet oxygen (^1^O_2_) and superoxide anion radical (O_2_^•‑^), operating via both energy and electron transfer pathways. These findings establish this Cp*Ir­(III) complex as an efficient, versatile, and sustainable photocatalyst, constituting one of the first examples of half-sandwich Ir complexes employed in diverse photooxidative organic reactions.

## Graphical abstract

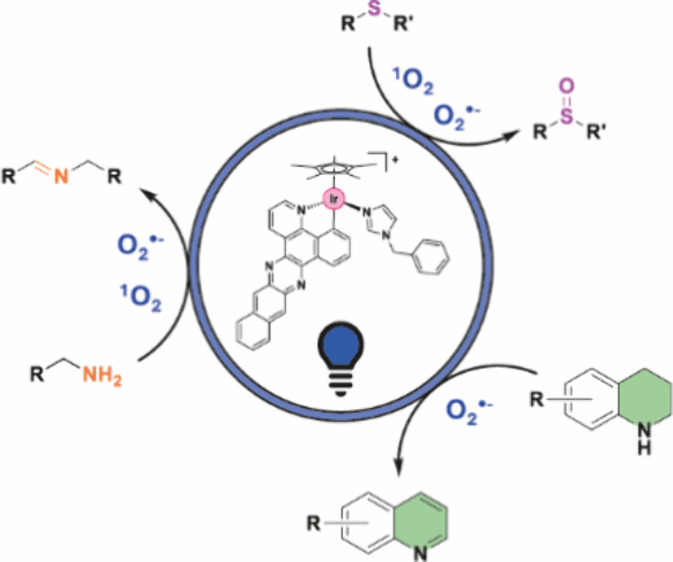

## 1 Introduction

In recent decades, the scientific community has focused on developing new strategies to carry out reactions under environmentally friendly conditions, either due to the energy crisis or to reduce byproduct formation.^1–4^ In this context, visible light-induced catalysis has emerged as a powerful tool to achieve this goal. Since the pioneering works by groups of McMillan,^5^ Yoon,^6^ Stephenson,^7^ and many others, photocatalysis have gained much attention. Photocatalysis relies on the use of complexes that can be excited by visible light, generating long-lived excited states, which are capable of activating chemical bonds, enabling the activation of small molecules and the formation of complex structures under mild conditions. The resultant excited species can act as both a strong oxidant and a strong reductant simultaneously, providing access to a reaction environment that is unique to organic chemistry. Furthermore, visible light energy is a mild, safe, and easy-to-handle energy source. The inherently weak absorption of visible light by most organic molecules participating in the transformations markedly lowers the probability of undesired side reactions.^8^ Together, these attributes make photocatalysis a greener, more sustainable strategy for chemical transformations that may be challenging or unattainable by using conventional methods.

Traditional methods for synthesizing organic compounds not only often rely on harsh conditions, including high temperatures, but also imply the use of toxic or corrosive oxidants or reductants. In contrast, photocatalysis offers a more sustainable alternative, typically requiring no additional additives, thereby enhancing the atom economy of the process. Notably, the environmentally friendly activation of oxygen to generate singlet oxygen (^1^O_2_) or other reactive oxygen species (ROS) presents a promising strategy for the selective oxidation of organic compounds under mild conditions—eliminating the need for stoichiometric strong oxidants and minimizing harmful byproducts or waste.

Among the various mechanisms in photocatalysis, one of the most common is photoinduced electron transfer, in which an electron is transferred between the substrate and the photocatalyst (PC).^9^ Alternatively, or in parallel, some systems can operate via an energy transfer mechanism, in which there is a physical energy transfer from the photoexcited species to an acceptor.^10,11^

Photocatalysis has found broad and diverse applications in organic synthesis. Sulfoxides are valuable intermediates in organic synthesis^12,13^ and are key structural motifs in numerous pharmaceutical drugs such as omeprazole, sulindac, and modafinil.^14^ Traditional synthetic protocols for these compounds typically require strong oxidants, high temperatures, and prolonged reaction times. These harsh conditions can lead to overoxidation of the sulfoxide derivatives into sulfones and result in the formation of large amounts of byproducts. In parallel, the oxidation of amines to imines using molecular oxygen and visible light is another valuable transformation in organic synthesis. Imine groups serve as key intermediates in the preparation of various fine chemicals, dyes, and pharmaceutical compounds.^15–17^ Traditionally, imines are synthesized via the direct condensation of aldehydes or ketones with amines, typically catalyzed by acetic acid through a dehydration process. However, this approach suffers from several limitations, including the need for unstable aldehyde substrates and the use of acidic conditions, which restrict its broader applicability in organic synthesis.^18–21^

Another class of compounds attracting considerable interest is quinolines and their derivatives, since they are commonly found in a variety of natural products and have shown potential as antibacterial, antifungal, and anticancer agents.^22–24^ In this context, the visible light-driven oxidation and dehydrogenation of N-heterocyclic compounds has emerged as a promising strategy for synthesizing quinoline-based intermediates used in drug development.^25–27^

Polypyridyl complexes based on ruthenium and iridium have been intensively studied as photocatalysts, due to their exceptional photophysical properties such as strong absorption in the visible region and long-lived photoexcited states.^28,29^ These photophysical properties can be finely tuned through rational ligand design, rendering these photocatalysts highly adaptable, depending on the chemical process. The key features that define an effective photocatalyst can be summarized as follows: (i) strong absorption in the visible spectrum, where most organic substrates do not interfere; (ii) long-lived photoexcited states—on the order of microseconds—sufficient for diffusion and reaction with substrates; (iii) the ability to generate highly oxidizing or reducing species in both the ground and excited states; and (iv) reversible electrochemical behavior and high photostability to prevent degradation during catalysis.^9^

Given the remarkable photophysical properties of iridium complexes, many have been successfully employed as photocatalysts in a range of light-driven transformations, although most of the photocatalysts are based on bis-cyclometalated Ir­(III) complexes.^8,30–37^ Concerning half-sandwich iridium complexes, most of the reported catalytic studies have been focused on their behavior in thermal processes, in traditional organometallic catalysis,^38–48^ mainly focused on transfer hydrogenation of unsaturated substrates. In contrast, relatively few investigations have explored their potential as photocatalysts. Mata et al. reported an outstanding half-sandwich Ir­(III) system, which was used in the dehydrogenation reaction of N-heterocycles under light irradiation.^49^ In an elegant study, Alemán and collaborators^50^ reported piano-stool Ir­(III) complexes based on oxyquinolinate ligands as photocatalysts exhibiting dual activity in dehalogenation reactions coupled with hydrogen transfer processes. Another remarkable contribution was made by Chirik,^51,52^ who reported the use of half-sandwich iridium hydride photocatalysts for blue light-driven hydrogenation of organic substrates.

Recently, we reported the first half-sandwich iridium cyclometalated complexes capable of acting as photodynamic therapy (PDT) agents, demonstrating exceptional singlet oxygen (^1^O_2_) generation quantum yields under visible-light irradiation (ϕ_Δ_ up to 99%).^53^ This exceptional behavior of the Ir­(III) derivatives, with formula [Cp*Ir­(C^∧^N)­L]^0/+^, was achieved thanks to the use of π-extended C^∧^N ligands. The remarkable bioactivity observed under light irradiation prompted us to investigate their photocatalytic behavior and explore the effect of structural modifications on their performance (Figure 1). Two different C^∧^N ligands were used: 4,9,16-triazadibenzo­[*a*,*c*]­naphthacene (pbpn) in complexes **1** and **3** and 4,9,14-triazadibenzo­[*a*,*c*]­anthracene (pbpz) in complex **2**. The coordinated L ligands were *N*-benzylimidazole (bzIm) in monocationic complexes **1** and **2** and chloride in neutral complex **3**. In this work, we report the electrochemical properties, the O_2_^•–^ generation, and the photocatalytic activity of three half-sandwich Ir­(III) complexes in three different oxidation reactions. To the best of our knowledge, this work represents the first examples of half-sandwich Ir­(III) photocatalysts applied to the oxidative coupling of amines and the selective oxidation of thioethers. Complex **1** exhibited exceptional catalytic performance, efficiently transforming a wide range of substrates into valuable molecules that serve as key scaffolds across various fields. This derivative operated with an exceptionally low photocatalyst loading—as low as 0.005 mol %—under mild, room-temperature conditions and without the need for an oxygen-enriched atmosphere in certain transformations. Reusability tests demonstrated that the photocatalyst can be recycled for up to five consecutive cycles without a significant loss of activity.

**Figure 1:**
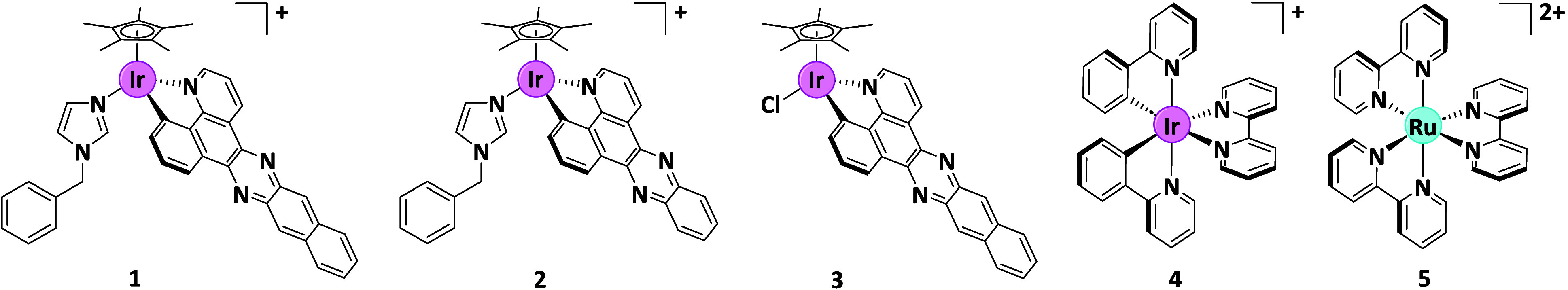
Photosensitizers (PSs) employed in this work. **4** and **5** were used as references.

## 2 Materials and Methods

### 2.1 Materials

IrCl_3_·*x*H_2_O was purchased from Johnson Matthey and used as received. Benzo­[*h*]­quinoline-5,6-dione was prepared as reported.^54^ The proligands and complexes were prepared as previously reported by us.^53^ For photocatalytic studies, benzylamine, 4-aminobenzylamine, DL-α-methylbenzylamine, (2,4-dimethoxyphenyl)­methanamine, 1-(4-methoxyphenyl)­ethan-1-amine, and 1-propylamine were purchased from Acros; 2-picolylamine, (R)-(+)-1-(1-naphthyl)-ethylamine, benzhydrylamine, cyclohexylamine, and tetrahydrothiophene from Aldrich; 4-picolylamine, 4-methoxybenzylamine, propargylamine, 4-chlorothioanisole, α-chlorothioanisole, bis­(chloromethyl)­sulfide, and 1,2,3,4-tetrahydroquinoline from TCI; 4-chlorobenzylamine, 4-nitrobenzylamine, tryptamine, 1-butylamine, and 1,2,3,4-tetrahydroisoquinoline from Alfa Aesar; 2-ethylhexylamine, thioanisole, di-*n*-propyl sulfide, 4-methylthioanisole, 4-nitrothioanisole, 4-(methylthio)­aniline, 4-(methylthio)­benzaldehyde, diphenyl sulfide, thiophene, dibenzothiophene, and indoline from ThermoScientific; and 4-cyanobenzylamine, 4-dimethylaminobenzylamine, 4-methylbenzylamine, and 4-methoxythioanisole from Indagoo.

### 2.2 Experimental Details

All synthetic manipulations were performed under an inert, oxygen-free, dry nitrogen atmosphere by using standard Schlenk techniques. All metal complexes were synthesized in dark conditions and protected from light using aluminum foil throughout each step of synthesis, isolation, and characterization. Solvents were dried and distilled under a nitrogen atmosphere begore use. Unless otherwise stated, reagents and solvents were of reagent quality and commercially available. Flash chromatography was carried out using silica gel or neutral alumina (Scharlau). UV–vis absorption spectra were recorded on a Secomam Uvikon XS spectrophotometer using the LabPower Junior program. Multinuclear NMR (nuclear magnetic resonance) spectra were recorded at 298 K on a Bruker 400 spectrometer or on a Bruker 500 spectrometer. Chemical shift values (δ) are reported in ppm (parts per million) and coupling constants (*J*) in Hertz. The splitting of proton resonances is defined as follows: s = singlet, d = doublet, t = triplet, q = quadruplet, m = multiplet, and bs = broad singlet. ^1^H NMR chemical shifts were internally referenced to C*H*D_2_CN (1.94 ppm) for acetonitrile-*d*_*3*_ and (C*H*D_2_)­(CD_3_)­CO (2.05 ppm) for acetone-*d*_*6*_ via the residual proton solvent resonances. The probe temperature (±1 K) was controlled by a standard unit calibrated with a methanol reference. All NMR data processing was performed by using MestReNova version 12.0.0.

No unexpected or unusually high safety hazards were encountered in this work.

### 2.3 Methods and Instrumentation

#### 2.3.1 Irradiation System

Light irradiation was performed in a 16-compartment “Medusa” photomultireactor built by Microbeam, equipped with high-power LEDs (2.3 W, 700 mA, Luxeon Rebel, Philips Lumileds). The LEDs were situated below each compartment (Figure S1). Every reactor position is composed of a 40 mL cylindrical glass flask provided by Scharlau. All reaction positions were irradiated from the bottom using monochromatic light provided by LEDs of blue (447 ± 20 nm, 59.2 mW cm^–2^), light blue (470 nm, 51.4 mW cm^–2^), green (530 nm, 29.1 mW cm^–2^), orange (591 nm, 36.1 mW cm^–2^), and red (655 nm, 80.6 mW cm^–2^) light. All reactions in the system were stirred using an orbital stirrer, which was set at 50 rpm.

#### 2.3.2 Stability and photostability studies

Solutions of all of the complexes at 2 mM in DMSO-*d*_6_ were monitored by ^1^H NMR spectroscopy in dark conditions and upon blue light irradiation (447 nm, 59.2 mW cm^–2^). The use of DMSO was due to the higher solubility of the complexes in this solvent than in acetonitrile.

#### 2.3.3 Photophysical Properties

For photophysical measurements, all solvents used were of spectroscopic grade. UV–vis absorption spectra were recorded on a Secomam Uvikon XS spectrophotometer at room temperature using the LabPower Junior program. Quartz cuvettes with a 1 cm optical path length were used for the measurements. Molar absorption coefficients (ε) were determined by direct application of the Beer–Lambert law, using solutions of the compounds with concentrations ranging from 10^–6^ to 10^–5^ M.

#### 2.3.4 Superoxide Anion Radical (O_2_^•–^) Generation

Dihydrorhodamine 123 (DHR123) was employed as a superoxide anion radical indicator. Ir­(III) complexes and [Ru­(bpy)_3_]^2+^ were prepared as 10 μM in water (1% DMSO) and DHR123 (TargetMol) was prepared as 30 μM in water (0.3% DMSO). In a quartz cell, 300 μL of PS solution and 1000 μL of DHR123 solution were mixed with 1700 μL of water to obtain a final solution (3 mL) of DHR123 (10 μM) and PS (1 μM) in water (0.2% DMSO). Then, the cuvette was exposed to blue light (470 nm, 51.4 mW cm^–2^) for indicated times, and the fluorescence spectra were immediately recorded. [Ru­(bpy)_3_]^2+^ was used as a reference. As control, DHR123 solution without PS was monitored under blue light irradiation. For superoxide anion radical quenching experiment, tiron was added to the above solution before light irradiation.

#### 2.3.5 Electron-Paramagnetic Resonance (EPR) Measurements

EPR spectra were acquired at room temperature using a MiniScope MS 400 X-band EPR spectrometer (Magnettech, Germany), with samples loaded into 10 μL glass capillaries (Duran Ringcaps, Hirschmann, Germany). Acquisition parameters for superoxide detection were as follows: four scans (60 s); gain, 500; modulation amplitude, 0.2 mT; microwave power, 50 mW; and microwave frequency, 9.45 GHz. Acquisition parameters for singlet oxygen detection were as follows: single scan (60 s); gain, 100; modulation amplitude, 0.1 mT; microwave power, 10 mW; and microwave frequency, 9.45 GHz. Irradiation was performed *in situ* using a UV-LED spot light source of 365 nm ± 2 nm and 2000 mW cm^–2^ (model LC-L1 V5 equipped with a L14310-115 LED, Hamamatsu Photonics K.J., Hamamatsu City, Japan).

#### 2.3.6 Electrochemical Measurements

CV was performed on a DropSens μStat400 potentiostat coupled to a Metrohm 757 VA Computrace cell at a potential sweep rate of 100 mV/s. A glassy carbon (GC) disk electrode (3 mm diameter) served as the working electrode, a coiled platinum wire was used as the counter electrode, and a Ag/AgCl (3 M KCl) electrode was used as the reference. Electrochemical measurements were performed in a 10 mL cell at an approximate complex concentration of 1 mM using 0.1 M of [*n*Bu_4_N]­[BF_4_] as supporting electrolyte in DMF. The samples were degassed by bubbling N_2_ into the solution for 10 min before the voltammograms were recorded to ensure that they were oxygen-free. The GC electrode was polished with a 0.05 μm γ-alumina slurry, rinsed clean with deionized water and ethanol, and subsequently dried. Each compound was obtained against its corresponding background, and all voltammograms were background-corrected.

#### 2.3.7 General Procedure for Photocatalysis Experiments

A 40 mL cylindrical glass flask was charged with 2 mL of an acetonitrile solution containing 50 mM of the corresponding substrate, 8 mM of the standard 1,3,5-trioxane, and the corresponding concentration of the photocatalyst. When an oxygen-enriched atmosphere was needed, the solution was bubbled with O_2_ for 2 min and then kept under an oxygen atmosphere using an O_2_-filled ballon (1 atm). For ^1^H NMR analysis, 50 μL aliquots were diluted in 450 μL of CD_3_CN. For reactions under an inert atmosphere, the same procedure was followed by bubbling N_2_ and using a N_2_-filled balloon (1 atm). For studies with scavengers, the solutions were prepared containing additional 11 mM of the corresponding scavenger. All the photocatalysis experiments were carried out in duplicate.

#### 2.3.8 Emission-Quenching Experiments

A solution of photocatalyst **1** (10 μM) in degassed acetonitrile was monitored by photoluminescence spectroscopy upon incremental concentrations (0–10 equiv) of benzylamine (**1a**), thioanisole (**3a**), and 1,2,3,4-tetrahydroquinoline (**6a**). Photoluminescence spectra were recorded upon irradiation at λ_exc_ = 420 nm on a PTI Quanta Master TM spectrofluorometer from Photon Technology International (PTI) equipped with a Xenon short arc lamp (75 W) and an 814PTM detector. Hellma quartz cuvettes of a 1 cm optical path length were used. Felix32 software was employed to collect and process the luminescence data. The quenching of the emission, reflected as the ratio *I*_0_/*I*, was represented versus the concentration of the quencher and fitted to the Stern–Volmer eq 1:

$$\frac{I_{0}}{I}=1+K_{\mathrm{SV}}\cdot \left[Q\right]$$ 1

where *I*_0_ is the maximum intensity of the emission spectrum in the absence of a quencher, *I* is the maximum intensity of the emission spectra in the presence of a quencher, *K*_SV_ is the Stern–Volmer constant, and [*Q*] is the concentration of the quencher.

#### 2.3.9 Reusability Study

The experiment was carried out using benzylamine **1a** as a substrate. The reaction was scaled up to a total volume of 5 mL. The solution contained 50 mM of the substrate, **1a**, 8 mM of the standard 1,3,5-trioxane, and 25 nM of photocatalyst **1** (0.05 mol %). 50 μL aliquots were taken from the crude of the reaction and subsequently diluted in 450 μL of CD_3_CN to analyze by ^1^H NMR spectroscopy. When the catalytic cycle was completed, fresh benzylamine was added (27.3 μL, 0.25 mmol) to obtain a final solution containing 50 mM of **1a**. A total of six cycles were studied.

## 3 Results and Discussion

### 3.1 Photostability Experiments

The stability of the three Ir­(III) complexes, previously reported by us,^53^ was studied by ^1^H NMR spectroscopy monitoring the evolution of the spectra in DMSO-*d*_*6*_, in dark conditions and upon blue light irradiation (LED, λ_ir_ = 447 nm, 59.2 mW cm^–2^) in aerated conditions at room temperature (Figures S2–S7). Blue light was selected as the excitation source, given that the visible absorption bands of the complexes are centered in the blue region of the spectrum.

Both cationic complexes exhibited good stability in dark conditions. Upon blue light irradiation, complex **1** showed good stability, with around 16% transformation after 24 h of light irradiation. This process was attributed to the photodissociation of the *N*-benzylimidazole (bzIm) ligand and the formation of the corresponding aqua adduct [Cp*Ir­(pbpn)­(D_2_O)]^+^, **1w**. In contrast, complex **2** exhibited a much faster photodissociation of the bzIm ligand to yield the aqua adduct [Cp*Ir­(pbpz)­(D_2_O)]^+^, **2w**, which was completed after only 1 h of light exposure. These observations suggest that irradiation promotes ligand exchange processes in coordinating media. The stability of complex **3** was also monitored in DMSO-*d*_*6*_. As previously described,^53^ under dark conditions, the chloride ligand is slowly exchanged by a water molecule from the aqueous traces in DMSO-*d*_*6*_, producing the corresponding aqua adduct, **1w**. Exposure to blue light enhances the rate of chloride ligand exchange in complex **3**.

### 3.2 Superoxide Radical Anion (O_2_^•–^) and Singlet Oxygen (^1^O_2_)

In some photocatalytic reactions, O_2_^•–^ plays a crucial role in the transformation of the reactants. An O_2_^•–^ generation experiment was conducted to determine the ability of these complexes to produce this radical, using the dihydrorhodamine 123 (DHR123) assay in water (0.2% DMSO), monitoring the emission change of the probe by fluorescence spectroscopy upon irradiation. The nonemissive DHR123 probe is oxidized by the photogenerated O_2_^•–^ into the fluorescent rhodamine 123, with a maximum emission at 526 nm. The experiment was carried out in dark conditions and upon blue light irradiation (470 nm, 51.4 mW cm^–2^) for 30 s using [Ru­(bpy)_3_]^2+^ as the reference. In dark conditions (Figure S8), no oxidation of the probe was detected. Upon blue-light irradiation, in the presence of complexes **1**, **2**, **3**, and [Ru­(bpy)_3_]^2+^, O_2_^•–^ generation was confirmed by the increase in the emission of oxidized rhodamine 123 (Figures S8 and S9). The three complexes produced O_2_^•–^ at rates comparable to those of the reference. The detection of O_2_^•–^ was further confirmed by repeating the experiment in the presence of tiron, a well-known selective inhibitor of O_2_^•–^ (Figure S10).

The ^1^O_2_ generation efficiency of these complexes was quantified in our previous work,^53^ where quantum yields of up to 99% were achieved.

Finally, the generation of both ^1^O_2_ and O_2_^•–^ under light irradiation by complexes **1** and **2** was further verified by electron-paramagnetic resonance (EPR) spectroscopy. EPR measurements were conducted in an acetonitrile solution in the presence of two specific radical spin-traps, 2,2,6,6-tetramethylpyridine (TEMP) and 5,5-dimethyl-1-pyrroline-*N*-oxide (DMPO). The spin-trapping agent TEMP selectively reacts with ^1^O_2_ to form the persistent nitroxide radical 2,2,6,6-tetramethylpiperidine-*N*-oxide (TEMPO), which displays a characteristic three-line EPR spectrum.^55–57^ Owing to its high thermodynamic and kinetic stability, TEMPO accumulates efficiently in the solution, enabling straightforward detection. The clear EPR signal observed after light irradiation (Figure S11) provides strong evidence for the generation of ^1^O_2_ by both complexes, **1** and **2**.

The reaction of the nitrone spin-trap DMPO with O_2_^•–^ leads to the formation of the DMPO-OOH spin adduct, which exhibits a characteristic quartet EPR signal arising from the hyperfine coupling with the nitroxide nitrogen and the adjacent β-hydrogen.^58^ This spin adduct is inherently unstable, undergoing rapid decay due to spontaneous rearrangement and degradation, which significantly restricts the detection window. Consequently, *in situ* irradiation was employed, and the resulting EPR spectra (Figure S12) confirmed the presence of aqueous O_2_^•–^ in the solution.

### 3.3 Electrochemical Measurements

The electrochemical properties of the three half-sandwich Ir­(III) complexes **1**–**3** and the two C^∧^N proligands Hpbpn and Hpbpz were evaluated by cyclic voltammetry (CV) in DMF. The cyclic voltammograms are presented in Figure 2, and the redox potentials, referenced to the ferrocene/ferrocenium (Fc/Fc^+^) couple, are summarized in Table 1.

**Figure 2:**
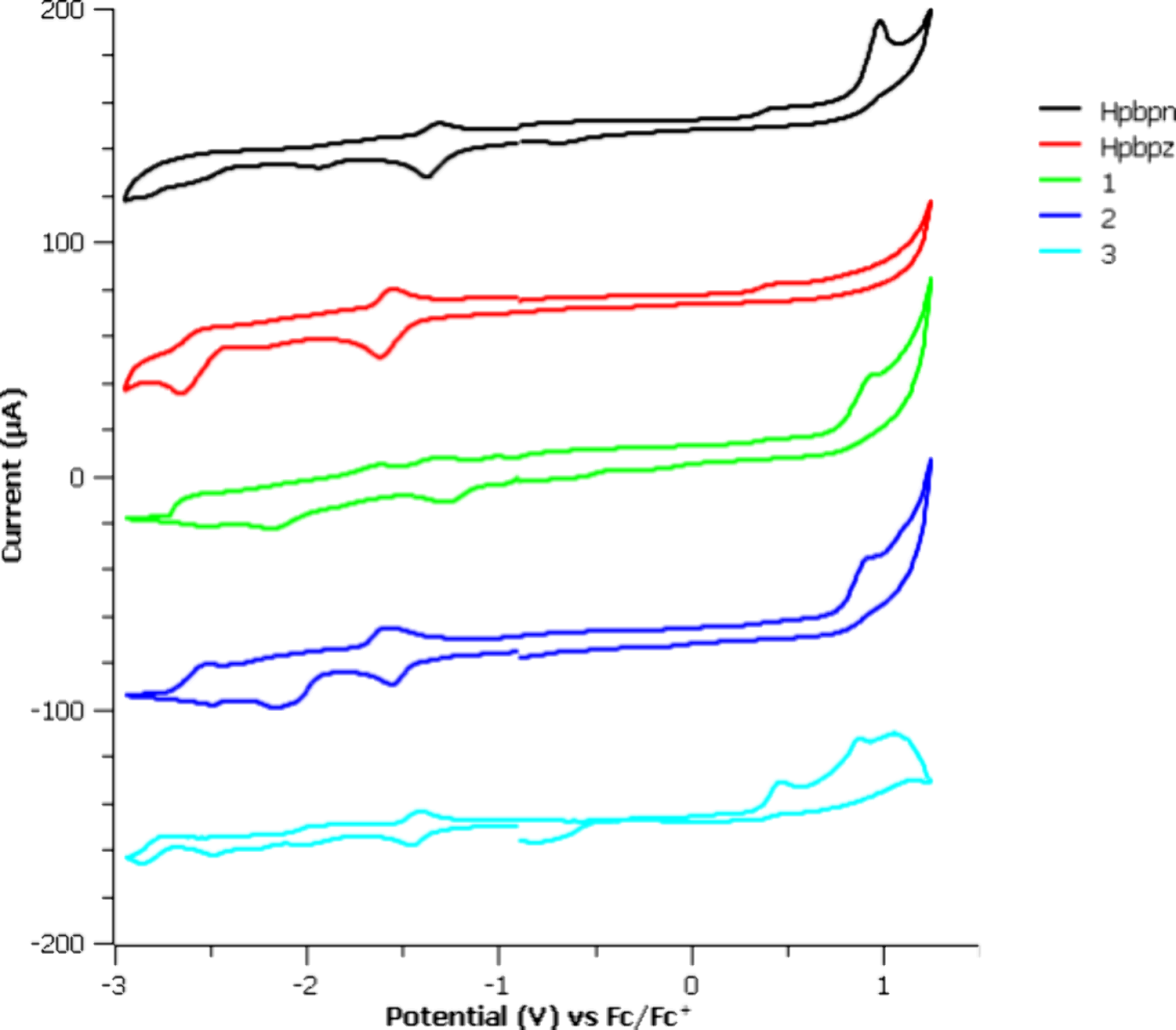
Cyclic voltammograms of proligands Hpbpn and Hpbpz and complexes **1**–**3** vs Fc/Fc^+^ in DMF.

**Table 1:** Redox Potentials of Proligands Hpbpn and Hpbpz, Complexes **1**–**3** and reference complexes **4** and **5** vs Fc/Fc^+^ Determined by Cyclic Voltammetry^a^

**compound**	***E***_**1**/**2**_^**ox**^ **(V)**	***E***_**1**/**2**_^**red**^ **(V)**	**Δ*E***_**1/2**_ **(V)**	***E***_**0–0**_ **(V)**	***E*** **_1/2_** **(PS** **^+^** /PS*) ^f^	***E***_**1/2**_ (PS*/PS ^**–**^ **)** ^g^
**Hpbpn**	+0.98 (ir)	–1.34 (qr)	2.32			
		–2.59 (ir)				
		–2.82 (ir)				
**Hpbpz**		–1.59 (qr)				
		–2.65 (qr)				
**1**	+0.95 (qr)	–1.14 (qr)	2.09	2.52	–1.57	+1.38
		–1.46 (qr)^e^				
		–2.18 (ir)				
**2**	+0.92 (qr)	–1.58 (qr)^e^	2.50	2.57	–1.65	+0.99
		–2.16 (ir)				
		–2.50 (qr)^e^				
**3**	+0.40 (qr)^d^	–1.43 (qr)^e^	2.26	2.51	–1.68	+1.08
		–1.98 (qr)				
	+0.83 (qr)	–2.25 (qr)				
		–2.49 (ir)				
		–2.79 (qr)^e^				
**4** ^b^	+0.87 (r)	–1.78 (r)	2.65		–1.19	+0.28
**5** ^c^	+0.90 (r)	–1.72 (r)	2.62		–1.10	+0.28
		–1.89 (r)				
		–2.15 (r)				

a Voltammograms recorded in DMF solutions (1 mM) of the compounds using 0.1 M of [*n*Bu_4_N]­[BF_4_] as supporting electrolyte with a scan rate of 0.10 V s^–1^ and referenced to Fc/Fc^+^ (r = reversible, qr = quasi-reversible, ir = irreversible). Δ*E*_1/2_ = *E*_1/2_^ox^ – *E*_1/2_^red^ = first reversible oxidation potential–first reversible reduction potential.

b From ref 63 in acetonitrile.

c From ref 64 inacetonitrile.

d Oxidation of Cl^–^.

e Peaks assigned to reduction of the C^∧^N ligands.

f *E*_1/2_(PS^+^/PS*) = *E*_*ox*,1_^1/2^ – *E*_0–0_.

g *E*_1/2_(PS*/PS^–^) = *E*_*red*,1_^1/2^ + *E*_0–0_.

The studies showed several redox processes. In the anodic region, one quasi-reversible peak was observed for complexes **1**–**2**, appearing at approximately +0.9 V. In the case of complex **3**, this peak appeared at +0.8 V, together with an additional quasi-reversible peak at +0.4 V, which was assigned to the oxidation of chloride bound to the metal center.^59^ The oxidation potentials can be associated with the HOMO orbitals. According to TD-DFT calculations, the HOMO orbitals of these complexes are composed of a combination of metal-centered dπ orbitals (≈43%) and π orbitals located in the orthometalated phenyl ring of the C^∧^N ligands (≈26%) and the Cp* moiety (≈25%).^53^ Notably, it was observed that only complex **1** possesses a nearly isoenergetic HOMO-1 orbital, which is essentially composed of π orbitals from the benzo-quinoxaline fragment (87%). However, given the similarity of the oxidation peaks in both complexes containing pbpn or pbpz ligands, the oxidations are attributed to the removal of an electron from Ir­(III)-(C^∧^N)-centered orbitals.

Regarding the cathodic region, multiple quasi-reversible reduction peaks were observed for all of the compounds. These reductions involve the promotion of an electron in the LUMO orbitals. TD-DFT calculations indicated that in complexes **1** and **2**, the LUMO orbitals are primarily composed (≈75%) of π orbitals from the benzoquinoxaline and quinoxaline fragments of the C^∧^N ligands, respectively. As the cyclic voltammograms of the free ligands also showed several reduction events, the cathodic peaks are likely associated with sequential reductions occurring on the pbpn and pbpz ligands.

The electrochemical band gap, Δ*E*_1/2_, of complex **1** (2.09 V), bearing the pbpn ligand, is smaller than that of complex **2** (2.50 V), with the pbpz ligand. These findings are in accordance with the HOMO–LUMO gaps calculated by TD-DFT calculations, which are 2.86 and 3.17 V for **1** and **2**, respectively. The narrower gap observed for complex **1** can be attributed to the greater stabilization of its LUMO, as a result from the larger electron-withdrawing character of the most π-extended ligand.^53^

To estimate the excited-state redox potentials of the complexes, both their ground-state redox potentials and excited-state energies (*E*_0-0_) were considered. *E*_0-0_ was determined using the method described by Weller,^60,61^ by identifying the intersection point between the lowest-energy absorption band in the UV–vis spectrum and the emission spectrum (Figure S13).^53,62^ Using the ground-state oxidation and reduction potentials, along with the estimated excited-state energies, the excited-state redox potentials of complexes **1**–**3** were calculated. The results are summarized in Table 1. These values indicate that complexes are expected to act as both strong photoreductants and photooxidants. Regarding the excited-state redox potentials, complexes **1**–**3** notably display greater reducing and oxidizing power compared to complexes **4** and **5**. Compared to other reported photocatalysts,^32^ these complexes exhibit higher excited-state redox potentials as well as longer excited-state lifetimes.

### 3.4 Photocatalysis

The photocatalytic activity of the half-sandwich Ir­(III) complexes was evaluated across three different transformations: the oxidative coupling of amines, the oxidation of thioethers, and the dehydrogenation of N-heterocycles. These reactions were selected to be explored due to their importance as key intermediates in the preparation of high-value molecules. Complex **1** was selected as model for testing the conditions, based on its excellent electrochemical and photophysical properties, suggesting its potential as an effective photocatalyst.

#### 3.4.1 Photocatalytic Oxidation of Benzylamine and Other Amines

In the initial set of experiments, the photocatalytic oxidative coupling of benzylamine (**1a**) with **1** was evaluated. This transformation was selected as a model reaction due to its well-established conversion into the corresponding imine (**2a**). The reaction was performed under mild conditions using 0.05 mol % complex **1**, under air, with blue light irradiation (LED, λ_ir_ = 447 nm, 59.2 mW cm^–2^), at room temperature in acetonitrile. Blue light was initially selected based on the UV–vis absorption spectra of the Ir­(III) complexes. Remarkably, the reaction reached completion within just 40 min, affording the imine product selectively in >99% yield (entry 1, Table 2), as confirmed by ^1^H NMR analysis of the crude reaction mixture. The reaction proceeded with high selectivity, producing only the desired imine, with no detectable byproducts from hydrolysis or overoxidation.

**Table 2:**
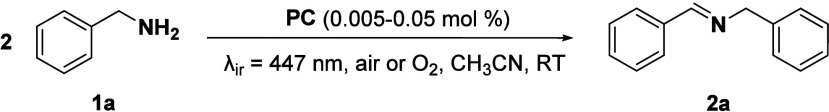
PC/Substrate Rate Screening in the Photooxidation of Benzylamine (**1a**) with **1** ^a^

**entry**	**[PC]/[1a] rate**	**[PC] (%)**	**conditions**	**time**	**conversion (%)**
1	1/2000	0.05	Air	40 min	99
2	1/20,000	0.005	Air	8 h	99
3	1/20,000	0.005	O_2_	3 h	99

a Reaction conditions: benzylamine (**1a**, 50 mM), PC (**1**, 0.005–0.05 mol %), acetonitrile (up to 2 mL), room temperature, under air or O_2_ (balloon, 1 atm), under irradiation with blue light (LED, λ_ir_ = 447 nm, 59.2 mW·cm^–2^), in a septum-capped tube. Conversions were experimentally determined from ^1^H NMR integration of the corresponding reaction crudes with 1,3,5-trioxane as internal standard. The conversion values were calculated as the mean of two independent experiments.

In view of these outstanding results, the catalyst loading was further dropped to 0.005 mol %, and the reaction was also performed under air. To our delight, the reaction was completed in 8 h with no O_2_-enriched atmosphere even under this very low content of PC, with a [PC]/[substrate] rate of 1/20,000 (entry 2, Table 2), demonstrating the exceptional activity of complex **1**. Moreover, we explored the effect of employing an O_2_-enriched atmosphere and the reaction time was diminished to just 3 h (entry 3, Table 2).

Then, the photocatalytic oxidative coupling of benzylamine (**1a**) was evaluated under irradiation with different light sources (Table 3). Low-energy visible-light sources such as green (530 nm), yellow (591 nm), and red (655 nm) LEDs resulted in negligible conversions (2–9%) to the corresponding imine (**2a**). Similarly, irradiation with white light led to only a 7% conversion. Based on these results, blue light was selected as the optimal irradiation wavelength for all subsequent photocatalytic studies.

**Table 3:** Irradiation Wavelength Screening in the Photooxidation of Benzylamine (**1a**) with **1** ^a^

**entry**	**λ**_**ir**_ **(nm)**	**conversion (%)**
1	447	99
2	530	9
3	591	2
4	655	2
5	white light	7

a Reaction conditions: benzylamine (**1a**, 50 mM), PC (**1**, 0.005 mol %), acetonitrile (up to 2 mL), room temperature, under air, under irradiation with blue (LED, λ_ir_ = 447 nm, 59.2 mW cm^–2^), green (LED, λ_ir_ = 530 nm, 29.1 mW cm^–2^), orange (LED, λ_ir_ = 591 nm, 36.1 mW cm^–2^), red (LED, λ_ir_ = 655 nm, 80.6 mW cm^–2^), or white light (35.0 mW cm^–2^) in a septum-capped tube. Conversions were experimentally determined from ^1^H NMR integration of the corresponding reaction crudes with 1,3,5-trioxane as internal standard. The conversion values were calculated as the mean of two independent experiments.

Based on these preliminary screenings of the catalytic activity, it was established that the optimal [PC]/[substrate] rate was 1/20,000 (0.005 mol %), operating under air atmosphere, at room temperature, and under blue light irradiation. Compared to previously reported systems employing half-sandwich iridium derivatives for the thermal catalytic coupling of amines to form imines,^65,66^ our photocatalytic system exhibits significantly superior performance. It operates under milder conditions, at room temperature rather than 65 °C, without the need for enriched O_2_-atmosphere oxygen and requires a catalyst loading that is up to 100 times lower, highlighting its remarkable selectivity, efficiency, and sustainability.

The photocatalytic activity of the remaining half-sandwich Ir­(III) complexes was also evaluated in this reaction (entries 2–3, Table 4) under these exceptional sustainable conditions. Notably, complex **3**, which also contains the pbpn ligand, displayed catalytic performance comparable to that of complex **1**. However, complex **2**, bearing a pbpz ligand, exhibited a lower activity, achieving a yield of 59% after 8 h of reaction. The activity of the two well-studied photocatalysts, **4** and **5**, was also explored (entries 4–5, Table 4). Under identical conditions, complex **5** achieved a modest 24% conversion, while complex **4** exhibited activity slightly lower to that of complexes **1** and **3**.

**Table 4:** Photocatalysts Screening in the Photooxidation of Benzylamine (**1a**)­^a^

**entry**	**PC**	**conversion (%)**
1	**1**	99
2	**2**	59
3	**3**	99
4	**4**	93
5	**5**	24

a Reaction conditions: benzylamine (**1a**, 50 mM), PC (0.005 mol %), acetonitrile (up to 2 mL), room temperature, under air and irradiation with blue light (LED, λ_ir_ = 447 nm, 59.2 mW·cm^–2^), during 8 h in a septum-capped tube. Conversions were experimentally determined from ^1^H NMR integration of the corresponding reaction crudes with 1,3,5-trioxane as internal standard. The conversion values were calculated as the mean of two independent experiments.

The results obtained for PCs **1**–**3** highlight the crucial role of the additional fused aromatic ring present in the more π-extended ligand pbpn in determining the photochemical properties of the resulting Ir­(III) complexes. The introduction of this extra ring reduces the HOMO–LUMO energy gap and markedly increases the ^3^LC (^3^ππ*) character of the lowest-lying triplet excited state, resulting in longer triplet excited-state lifetimes, as previously reported by us.^53^ Longer triplet lifetimes are advantageous for photochemical processes, as they increase the probability of efficient energy- or electron-transfer processes with the substrates. Consistent with this interpretation, the pbpz-containing complex **2**, which lacks this extra ring, exhibits a lower ^3^LC contribution and shorter triplet excited-state lifetime and consequently displays reduced catalytic activity. In contrast, both pbpn derivatives, **1** and **3**, differing in the monodentate ligand, show similar photocatalytic behavior, suggesting that the nature of this ligand has a negligible influence on the excited-state properties. These observations indicate that the photocatalytic activity primarily originates from the “Cp*Ir­(C^∧^N)” framework, while π-extension of the cyclometalating ligand plays the dominant role in tuning the photophysical and photocatalytic properties of these systems.

To further demonstrate the excellent catalytic performance of **1**, the substrate scope was extended to a broad range of amine derivatives (Figure 3). Similar conditions were employed to evaluate the effect of different groups on the oxidation of the amines **1b**–**1u** and subsequent coupling to yield the corresponding secondary imines (**2b**–**2u**). Notably, the oxidative coupling of primary amines, such as picolylamines (**1b** and **1c**), proceeded with excellent selectivity and high yields, underscoring the efficiency and versatility of the catalytic system. The reaction also proceeded in good yields with different electron-donating and electron-withdrawing groups in the *p*-position, such as chloride (**1d**), nitrile (**1e**), methyl (**1h**), and methoxy (**1j**). However, a significant reduction in the conversion to the corresponding imine was found for the case of substrates with dimethylamino (**1g**) and amino (**1i**) groups in the *p*-position. The reaction also failed in the oxidative coupling of strongly electron-deficient *p*-nitrobenzylamine (**1f**). The introduction of an additional methoxy group in the *o*-position in substrate **1k** also produced a decrease in the reaction rate, achieving a 29% conversion under standard conditions.

**Figure 3:**
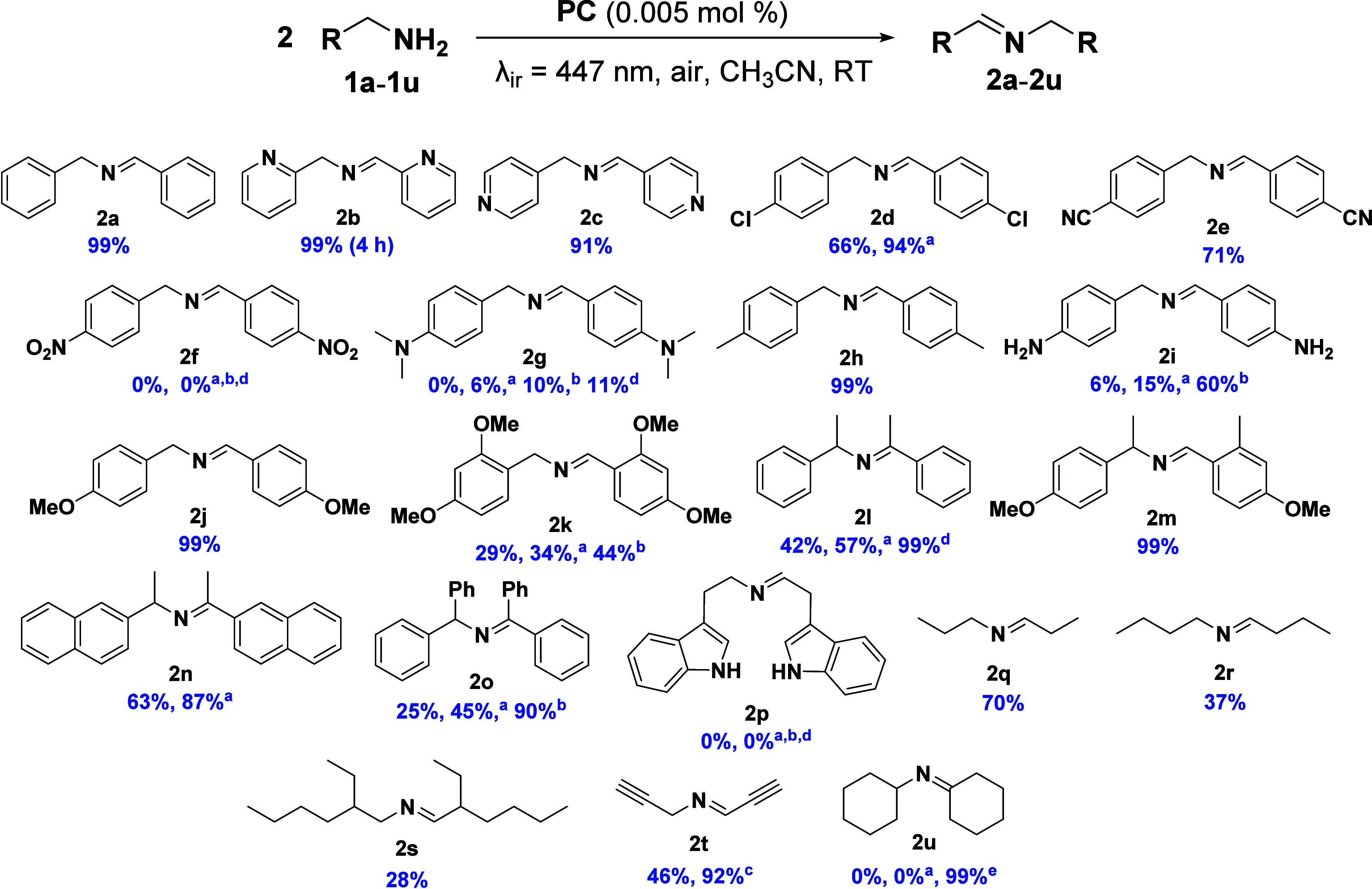
Photocatalytic oxidative coupling of amines **1a**–**1u**. Reaction conditions: amine derivative (50 mM), PC (**1**, 0.005 mol %), acetonitrile (up to 2 mL), room temperature, under air and irradiation with blue light (LED, λ_ir_ = 447 nm, 59.2 mW cm^–2^) during 8 h in a septum-capped tube. Conversions were experimentally determined from ^1^H NMR integration of the corresponding reaction crudes with 1,3,5-trioxane as internal standard. The conversion values were calculated as the mean of two independent experiments. ^a^In the presence of an O_2_-enriched atmosphere (balloon, 1 atm), PC (**1**, 0.005 mol %). ^b^56 h of reaction under standard conditions. ^c^72 h of reaction in standard conditions. ^d^8 h of reaction in an O_2_-enriched atmosphere (balloon, 1 atm) with 0.05 mol % of **1**. ^e^2 h of reaction under air conditions with 0.5 mol % of **1**.

The reaction also proceeded with secondary amines, such as DL-α-methylbenzylamine (**1l**), 1-(4-methoxyphenyl)­ethan-1-amine (**1m**), 1-(naphthalen-2-yl)­ethan-1-amine (**1n**), and benzhydrylamine (**1e**). However, under the general reaction conditions, complete conversion was only achieved in the case of **1m**, likely due to the electron-donating methoxy group in the *p*-position. Increasing the O_2_ concentration (O_2_, 1 atm) led to a significant improvement in reaction yields for **1n** and **1o**; nevertheless, complete conversion of **1l** was achieved under an O_2_-enriched atmosphere and with higher catalyst loading (0.05 mol %). In the case of tryptamine (**1p**), no reaction was observed under different reaction conditions.

In the case of purely aliphatic amines, such as *n*-propylamine (**1q**), *n*-butylamine (**1r**), and 2-ethylhexylamine (**1s**), the reaction proceeded more slowly but remained selective. Notably, propargylamine (**1t**) also underwent the reaction with high selectivity, reaching near-complete conversion after 72 h. In contrast, the significant steric hindrance presented by cyclohexylamine (**1u**) severely impeded the reaction, effectively inhibiting it at this low content of catalyst. Conversely, it is worth noting that even a low photocatalyst loading of 0.5 mol % was sufficient to complete the oxidation and coupling reaction of the sterically hindered imine, demonstrating the potential of complex **1**. Taken together, these results demonstrate that electron-withdrawing substituents diminish the reaction, whereas electron-donating substituents accelerate the rate of oxidative coupling of the resulting imines.

A comparison with representative photocatalytic systems reported in the literature is provided in Table S1, allowing a direct evaluation of the catalytic performance of PC **1** with respect to previously reported systems. Notably, PC **1** exhibits efficient catalytic activity under relatively mild conditions and low catalyst loadings, achieving one of the lowest PC loadings reported to date for this transformation.

Following these promising results, a series of control experiments were carried out to confirm the photocatalytic nature of the reaction and to elucidate the role of molecular oxygen. As anticipated, no product formation was observed in the absence of either light or the PC, confirming that the transformation is indeed light-mediated and dependent on the presence of the PC (entries 3–4, Table 5).

**Table 5:** Control Experiments for the Photooxidation of Benzylamine **1a** with **1** as PC^a^

**entry**	**conditions**	**conversion (%)**
1	PC, **O**_**2**_, light	99 (3 h)
2	PC, **air**, light	99
3	PC, air, **no light**	0
4	**no PC**, O_2_, light	0
5	PC, **N**_**2**_, light	23
6	PC, air, **65 °C**, **no light**	0
7	PC, air, light, **DABCO** ^b^	9
8	PC, air, light, **TEMPO** ^b^	12
9	PC, air, light, **BQ** ^b^	35
10	PC, air, light, *t* **BuOH** ^b^	99

a Reaction conditions: benzylamine (**1a**, 50 mM), PC (**1**, 0.005 mol %), acetonitrile (up to 2 mL), room temperature, under air or a saturated atmosphere of either O_2_ or N_2_ (balloon, 1 atm), under irradiation with blue light (LED, λ_ir_ = 447 nm, 59.2 mW·cm^–2^) during 8 h in a septum-capped tube. Conversions were experimentally determined from ^1^H NMR integration of the corresponding reaction crudes with 1,3,5-trioxane as internal standard. The conversion values were calculated as the mean of two independent experiments.

b 11 mM of DABCO, TEMPO, 1–4-benzoquinone (BQ), and *tert*-butanol (*t*BuOH) scavengers.

The results indicate that conducting the reaction under ambient air is sufficient to achieve complete conversion of the model substrate in 8 h (entry 2, Table 5). Notably, when the same reaction was performed under an oxygen atmosphere, a significant rate acceleration was observed (entry 1, Table 5, complete conversion in 3 h). In contrast, under a nitrogen atmosphere, only 23% conversion was attained (entry 5, Table 5) highlighting the crucial role of oxygen in promoting the reaction. Finally, the reaction was also investigated under thermal conditions (65 °C, entry 6, Table 5); however, no conversion to the imine product was detected after 8 h of reaction.

To identify the reactive oxygen species (ROS) involved in this process, the reaction was performed in the presence of a wide variety of quenchers. When the reaction was performed in the presence of DABCO, a well-known ^1^O_2_ scavenger, the yield of the reaction was strongly dropped to 9% (entry 7, Table 5), suggesting that ^1^O_2_ is involved in the oxidation. Further experiments were carried out in the presence of TEMPO and 1,4-benzoquinone (BQ) as general radicals and O_2_^•–^ quenchers, respectively, and the yield dropped to 12 and 35% (entries 8–9, Table 5), suggesting the participation of O_2_^•–^ and possibly other radicals in this transformation. Finally, the reaction was also evaluated in the presence of *tert*-butanol (*t*BuOH) as HO^•^ quencher, but it proceeded quantitatively (entry 10, Table 5), excluding HO^•^ as an oxidant in this reaction.

A plausible photocatalytic mechanism for the transformation of benzylamine (**1a**) is proposed in Figure 4 based on our results and previously reported studies.^67–69^ Considering the effect of ^1^O_2_ and O_2_^•–^ scavengers, the reaction is proposed to proceed via two distinct pathways: energy transfer and electron transfer mechanisms (Figure 4).

**Figure 4:**
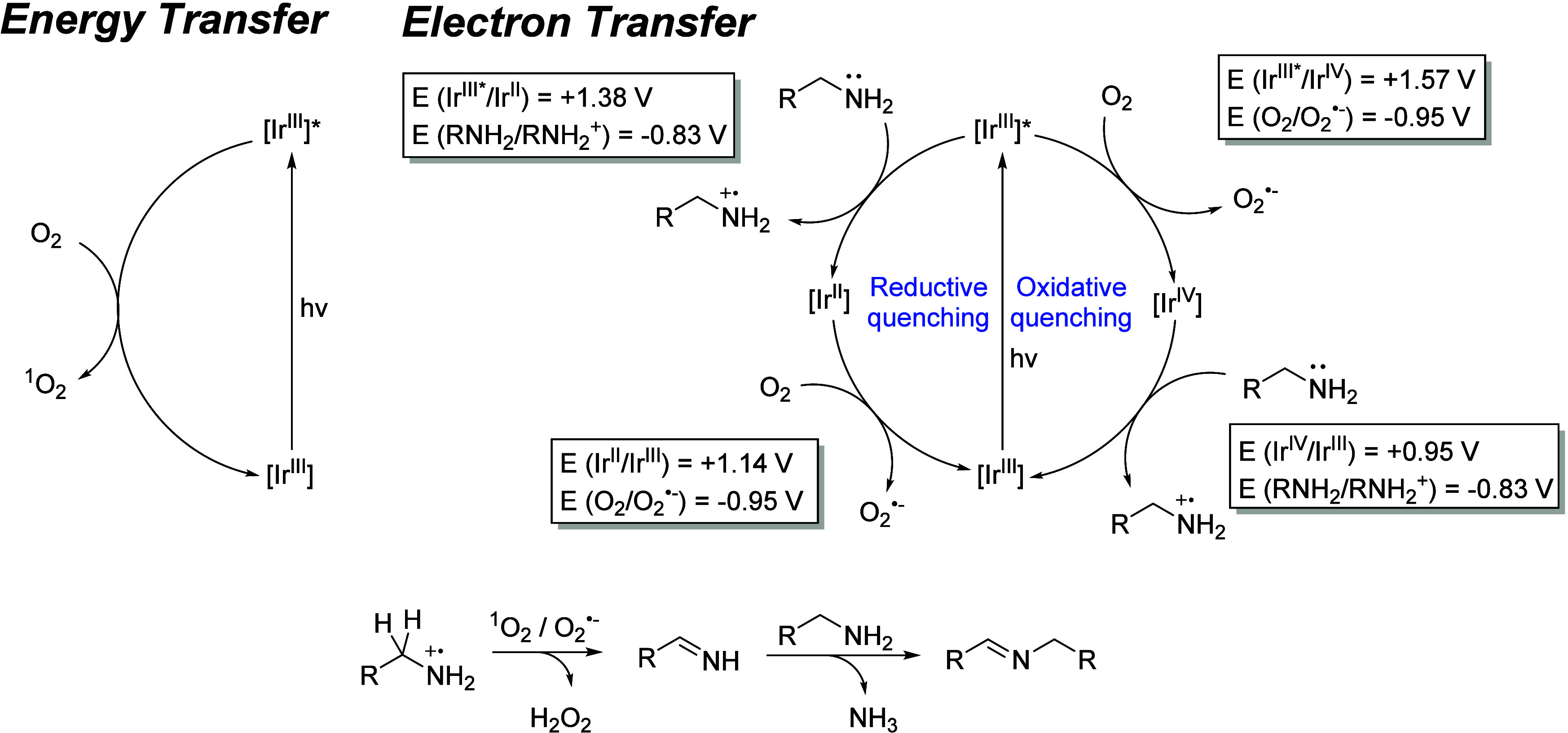
Plausible mechanism of the photocatalytic oxidative coupling of benzylamine (**1a**) with complex **1**.

Following the electron transfer pathway and based on the redox potentials of the PC in both its ground and excited states, and considering the case of benzylamine (**1a**) (E°(bzNH_2_/bzNH_2^•^_^+^) = −0.83 V),^70^ two thermodynamically feasible quenching pathways can be proposed. In the reductive quenching pathway, the PC is initially promoted to a singlet excited state upon photoexcitation, and then, it undergoes intersystem crossing (ISC) to generate a long-lived triplet excited state, [Ir^III^]*. In this state, excited PC [Ir^III^]* can oxidize **1a** by accepting an electron, thereby forming the reduced species [Ir^II^]. The PC is subsequently regenerated via electron transfer to molecular oxygen, generating superoxide radical anion O_2_^•–^. In contrast, in the oxidative quenching pathway, the photoexcited [Ir^III^]* species is first oxidized by molecular oxygen, which acts as an electron acceptor to produce O_2_^•–^. The oxidized PC, [Ir^IV^], is then able to oxidize **1a**, yielding the corresponding amine radical cation and regenerating the PC. In the case of the energy transfer pathway, upon photoexcitation, the triplet excited state, [Ir^III^]* can react with molecular oxygen, generating ^1^O_2_. Both O_2_^•–^ and ^1^O_2_ then participate in the oxidation of the benzylamine to form the corresponding primary imine intermediate, which finally undergoes a condensation reaction with a second molecule of **1a** to afford the secondary imine product **2a**. These findings clearly support the strong oxidizing character of the complex in its excited state.

Additionally, emission-quenching experiments were performed to further ascertain the most feasible quenching mechanism. A solution of photocatalyst **1** (10 μM) in degassed acetonitrile was studied in the presence of increasing concentrations of benzylamine **1a** (0–10 equiv). As shown in Figure S14, the emission of **1** was progressively diminished upon incremental addition of **1a**, thereby demonstrating quenching of the triplet excited state by **1a** and supporting the proposal of an electron transfer mechanism, with a Stern–Volmer quenching constant of K_SV_ = 2.80 × 10^3^ M^–1^.

#### 3.4.2 Photocatalytic Oxidation of Thioanisole and Other Thioether Derivatives

After the excellent photocatalytic activity of complex **1** in the oxidative coupling of amines, we decided to expand the catalytic behavior of the complex to other oxidative reactions. For this purpose, the oxidation of thioether derivatives to the corresponding sulfoxides and/or sulfones was investigated. As a model reaction, the photocatalytic oxidation of thioanisole (**3a**) was selected. The reaction was initially attempted using a PC loading of 0.005 mol % under ambient air conditions. However, this resulted in only very low yields (entries 1–2, Table 6). Increasing the PC loading 10-fold to 0.05 mol % enabled the reaction to reach completion within 24 h under the same air conditions (entry 3, Table 6) and in a very selective manner to the sulfoxide derivative **4a** and not to the sulfone species **5a**. Most reported thioether oxidation systems require an O_2_-enriched atmosphere^71,72^ and often demand strict control over reaction times or the use of additives such as hydrogen peroxide (H_2_O_2_) to avoid the over oxidation of the substrates, yielding mixtures of sulfoxide and sulfone derivatives.^73–75^

**Table 6:**
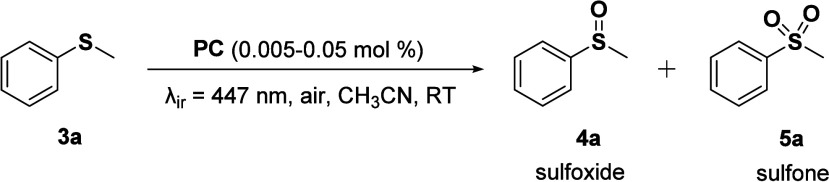
PC/Substrate Rate Screening in the Photooxidation of Thioanisole (**3a**) with **1** ^a^

**entry**	**[PC]/[3a] rate**	**[PC] (%)**	**time (h)**	**conversion (%)**
1	1/20,000	0.005	8	2
2	1/20,000	0.005	24	4
3	1/2,000	0.05	24	99

a Reaction conditions: thioanisole (**3a**, 50 mM), PC (**1**, 0.005–0.1 mol %), acetonitrile (up to 2 mL), room temperature, under air and irradiation with blue light (LED, λ_ir_ = 447 nm, 59.2 mW·cm^–2^), in a septum-capped tube. Conversions were experimentally determined from ^1^H NMR integration of the corresponding reaction crudes with 1,3,5-trioxane as internal standard. The conversion values were calculated as the mean of two independent experiments.

Remarkably, our system efficiently employs molecular oxygen from ambient air to achieve highly selective thioether oxidation under visible light without requiring any additional reagents. This highlights once again the exceptional photocatalytic performance of complex **1**.

The activity of photocatalyst **1** was compared with complexes **2** and **3** and the reference complexes **4** and **5** under similar conditions. As it can be seen in Table 7, the photocatalytic activity of **1** in the oxidation of thioanisole (**3a**) is comparable to that of reference complex **4** and significantly higher than that of complexes **2** and **3**. Notably, complex **5** showed no catalytic activity under these conditions.

**Table 7:** Photocatalyst Screening in the Photooxidation of Thioanisole (**3a**)­^a^

**entry**	**PC**	**Conversion (%)**
1	**1**	99
2	**2**	40
3	**3**	66
4	**4**	99
5	**5**	1

a Reaction conditions: thioanisole (**3a**, 50 mM), PC (0.05 mol %), acetonitrile (up to 2 mL), room temperature, under air and irradiation with blue light (LED, λ_ir_ = 447 nm, 59.2 mW cm^–2^), during 24 h in a septum-capped tube. Conversions were experimentally determined from ^1^H NMR integration of the corresponding reaction crudes with 1,3,5-trioxane as internal standard. The conversion values were calculated as the mean of two independent experiments.

Encouraged by the remarkable photocatalytic performance of complex **1**, we explored the oxidation of diverse thioether derivatives to assess the influence of different substituents (Figure 5).

**Figure 5:**
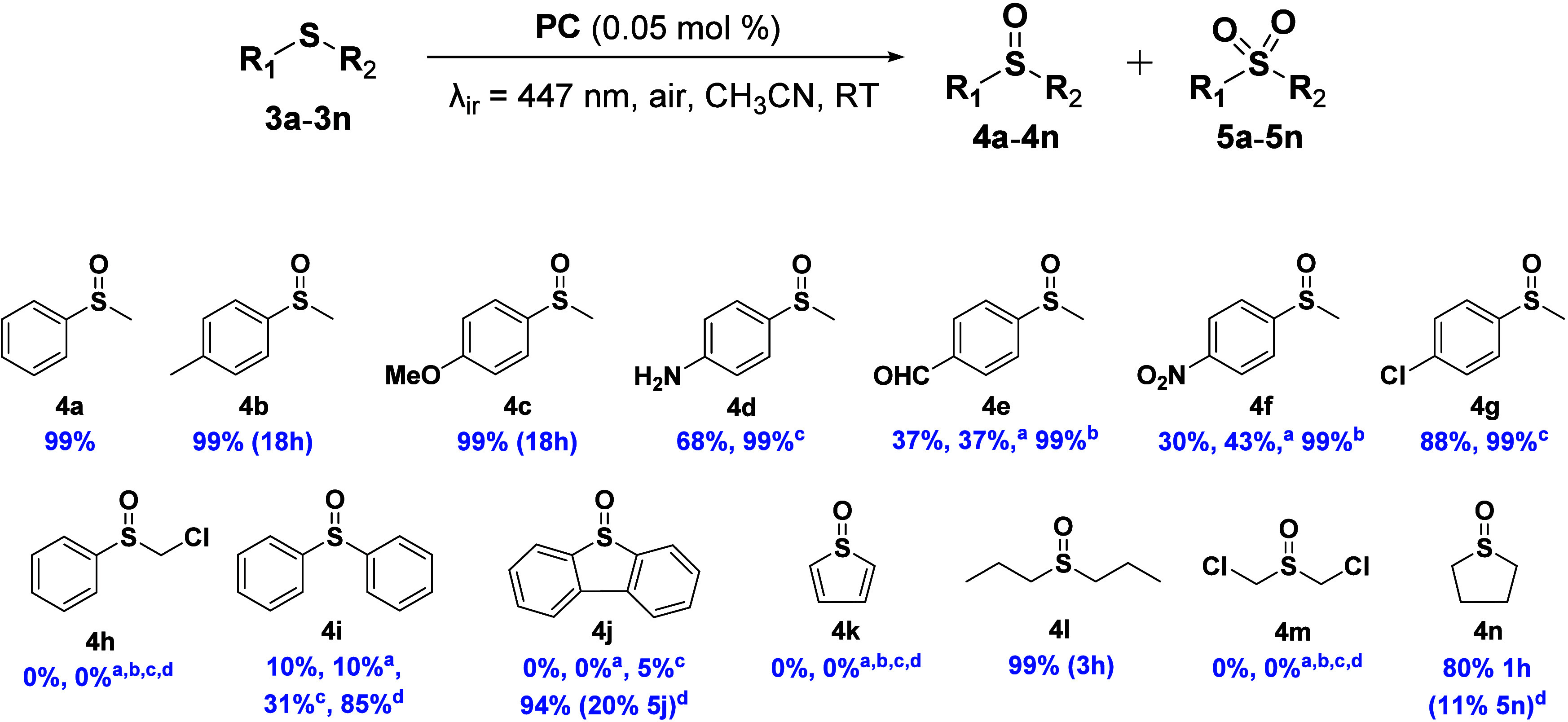
Photocatalytic oxidation of thioether derivatives **3a**–**3n**. Reaction conditions: thioether derivative (50 mM), PC (**1**, 0.05 mol %), acetonitrile (up to 2 mL), room temperature, under air and irradiation with blue light (LED, λ_ir_ = 447 nm, 59.2 mW cm^–2^) during 24 h in a septum-capped tube. Conversions were experimentally determined from ^1^H NMR integration of the corresponding reaction crudes with 1,3,5-trioxane as internal standard. The conversion values were calculated as the mean of two independent experiments. ^a^In the presence of an O_2_-enriched atmosphere (balloon, 1 atm). ^b^72 h of reaction in the presence of an O_2_-enriched atmosphere (balloon, 1 atm). ^c^8 h of reaction with 0.5 mol % of **1**. ^d^48 h of reaction with 0.5 mol % of **1**. Unless otherwise stated, quantitative selectivity toward the sulfoxide derivative was obtained; percentages in brackets represent the selectivity toward sulfone derivative.

Regarding aromatic substrates, the reaction rate was slightly enhanced by electron-donating groups in the *p*-position, such as methyl (**3b**) and methoxy (**3c**), leading to complete conversion within just 18 h, whereas the amine group (**3d**) produced a moderate reduction in the conversion. In contrast, electron-withdrawing groups, such as aldehyde (**3e**) and nitro (**3f**), exerted a pronounced negative impact on the reaction efficiency, requiring an O_2_-enriched atmosphere and prolonged reaction times to achieve quantitative conversion. Upon forcing the reaction conditions for **3e**, different overoxidation products were detected by ^1^H NMR, reducing selectivity to **4e** to 78%. Even at low conversion levels, an additional product in addition to **3e** and **4e** was detected. Analysis by ^1^H NMR revealed that the resonance of the methyl group of this byproduct closely resembles that of **4e**, suggesting the possibility of further oxidation occurring within the molecule—potentially at the aldehyde moiety of **4e**—under the reaction conditions.

The presence of a chloride group in the *p*-position (**3g**) did not significantly reduce the conversion to the corresponding sulfoxide (**4g**). In contrast, when α-chlorothioanisole (**3h**) was used, no reaction was detected even when the reaction was carried out under an oxygen-enriched atmosphere or when higher PC loading (0.5 mol %) was employed.

The reaction was also studied with diphenyl sulfide (**3i**). As expected, the substitution of the methyl group from thioanisole by a phenyl ring markedly reduced the conversion to only 10% under the standard conditions. Only significant yields were obtained when high PC loading was used (0.5 mol %). The oxidation of a polycyclic aromatic derivative such as dibenzothiofurane (**3j**) was also evaluated. The results suggest that such a substrate is more challenging to oxidize, requiring an increased catalyst loading (0.5 mol %) to achieve full conversion. Compared to **3i**, this observation indicates that when rotational freedom of the aromatic rings is restricted (as in **3j**), the oxidation becomes significantly more difficult and less selective, resulting in the formation of 20% of the corresponding sulfone derivative **5j**. In the case of thiophene (**3k**), no reaction was obtained in any case.

The oxidation of aliphatic derivative **3l** was also tested under standard conditions, resulting in complete conversion within just 3 h and high selectivity for the corresponding sulfoxide. This finding highlights the exceptional efficiency of complex **1** in the oxidation of aliphatic thioethers. However, the presence of chloride substituents in bis­(chloromethyl)­sulfoxide (**3m**) hinders the reaction. In the case of tetrahydrothiophene (**3n**), the reaction was fast, achieving an 80% conversion within just 1 h, but selectivity was affected, obtaining 89% of **4n** (sulfoxide derivative) and 11% of **5n** (sulfone derivative).

Finally, this model reaction was also performed under environmentally benign conditions, employing a 1:1 H_2_O/MeCN solvent system or natural sunlight as the energy source. Both approaches afforded the selective oxidation of **3a** to corresponding sulfoxide **3b**, with comparable reaction rates. Taken together, these results demonstrate that a wide variety of substituents are well tolerated in this reaction, underscoring the potential of complex **1** for the efficient oxidation of diverse thioether derivatives.

When compared with previously reported photocatalytic systems for the oxidation of thioethers (Table S2), PC **1** ranks among the most active PCs for this light-driven transformation. Under the optimized conditions, PC **1** enables complete conversion of thioanisole (**3a**) to the corresponding sulfoxide (**4a**) with excellent selectivity. Notably, these results are achieved under air conditions and low PC loadings, while most reported systems operate under an oxygen-enriched atmosphere and require higher catalyst loadings.

In order to elucidate the possible oxidative mechanism, a series of control experiments was performed. First, it was observed that the reaction did not proceed in the absence of light, PC, or oxygen (air) nor when it was conducted under thermal conditions (65 °C) in the dark. These results indicate that the transformation is light-driven and requires both the PC and air (entries 2–5, Table 8). The reaction was further investigated in the presence of various ROS scavengers. The addition of DABCO completely inhibited the reaction (entry 6, Table 8), suggesting the involvement of ^1^O_2_ in the process. Similarly, the addition of TEMPO nearly completely suppressed the reaction (entry 7, Table 8), indicating that it proceeds via a radical-mediated pathway. Additional experiments using quenchers such as BQ, *t*BuOH, and 1,4-dimethoxybenzene (DMB), inhibitor of thioether radical cations (R_2_S^•+^),^76^ revealed that other radical species—including O_2_^•‑^ and R_2_S^•+^—participate in the oxidation mechanism, whereas HO^•^ radicals are not implicated (entries 8–10, Table 8).

**Table 8:** Control Experiments for the Photooxidation of Thioanisole (**3a**)­^a^

**entry**	**conditions**	**conversion (%)**
1	PC, **air**, light	99
2	PC, air, **no light**	0
3	**no PC**, air, light	0
4	PC, **N**_**2**_, light	18
5	PC, air, **65 °C**, **no light**	0
6	PC, air, light, **DABCO** ^b^	0
7	PC, air, light, **TEMPO** ^b^	3
8	PC, air, light, **BQ** ^b^	54
9	PC, air, light, *t* **BuOH** ^b^	99
10	PC, air, light, **DMB** ^b^	63

a Reaction conditions: thioanisole (**3a**, 50 mM), PC (**1**, 0.05 mol %), acetonitrile (up to 2 mL), room temperature, under air or a saturated atmosphere of N_2_ (balloon, 1 atm), under irradiation with blue light (LED, λ_ir_ = 447 nm, 59.2 mW cm^–2^) during 24 h in a septum-capped tube. Conversions were experimentally determined from ^1^H NMR integration of the corresponding reaction crudes with 1,3,5-trioxane as internal standard. The conversion values were calculated as the mean of two independent experiments.

b 11 mM of DABCO, TEMPO, 1,4-benzoquinone (BQ), *tert*-butanol (*t*BuOH) or 1,4-dimethoxybenzene (DMB) scavengers.

Based on these findings and previous reports,^71^ a plausible mechanism for the oxidation of thioanisole (**3a**) is proposed (Figure 6). The reaction can proceed via two alternative pathways: electron transfer and energy transfer. Regarding the electron transfer process, two different mechanisms can be identified (Figure S15), but only the reductive quenching pathway is thermodynamically favored based on the redox potentials of the PC and the substrate (E°(RSR’/RSR’^•+^) = −1.02 V).^77^ In this quenching pathway, the PC in its excited triplet state [Ir^III^]* is reduced to generate an [Ir^II^] species, while simultaneously oxidizing the thioether to form the RSR’^•+^ intermediate. In the subsequent step, [Ir^II^] is oxidized back to regenerate the PC in its ground state, while molecular oxygen is reduced to produce O_2_^•–^. Alternatively, the PC in its triplet excited state, [Ir^III^]*, can efficiently interact with molecular oxygen through an energy transfer process to generate ^1^O_2_. Both O_2_^•–^ and ^1^O_2_ then react with the RSR’^•+^ specie, generating the persulfoxide intermediate, which subsequently reacts with a second molecule of thioether, yielding two equivalents of the corresponding sulfoxide (**4a**).

**Figure 6:**
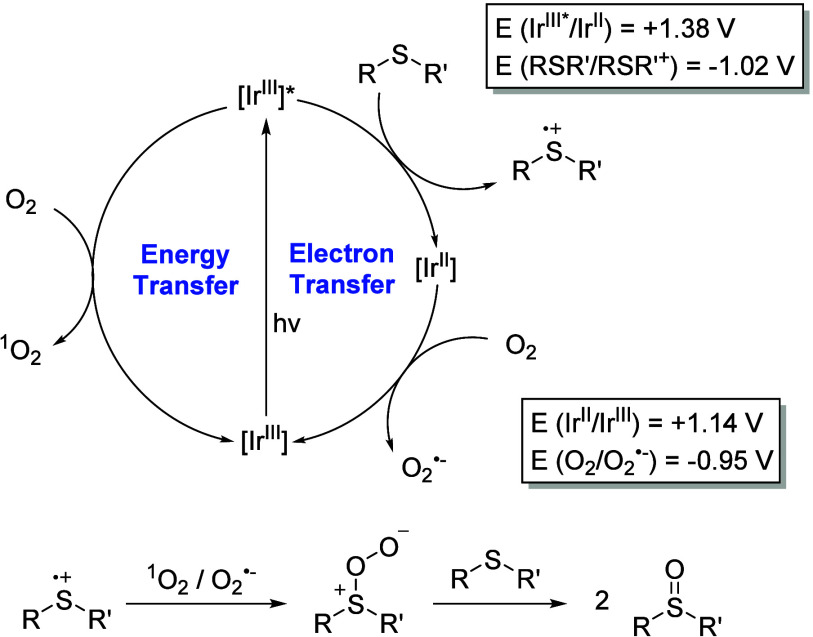
Plausible mechanism of the photocatalytic oxidation of thioanisole (**3a**) with half-sandwich Ir­(III) complex **1**.

Stern–Volmer quenching experiments were also performed for the case of thioanisole (**3a**). An acetonitrile solution of photocatalyst **1** (10 μM) in an inert atmosphere was studied in the presence of increasing concentrations of **3a** (0–10 equiv). In this case, the addition of the substrate strongly diminished the photoluminescence of **1** (Figure S16), confirming quenching of the excited state by the thioether. The calculated Stern–Volmer quenching constant was K_SV_ = 1.16 × 10^4^ M^–1^.

#### 3.4.3 Photocatalytic Dehydrogenation of 1,2,3,4-Tetrahydroquinoline and Other N-heterocycles

Finally, the photocatalytic activity of the half-sandwich Ir­(III) complexes was investigated in the dehydrogenative of N-heterocycles due to the significance of these structures in various natural products with valuable applications. 1,2,3,4-Tetrahydroquinoline (**6a**) was chosen as a model substrate to explore this type of photocatalysis. An initial attempt using 0.1 mol % of **1** under an O_2_-enriched atmosphere resulted in incomplete conversion after 24 h (entry 1, Table 9). Subsequently, the PC loading was increased up to 0.25 mol %, achieving total conversion after 24 h of reaction (entry 2, Table 9). The reaction afforded the double dehydrogenation product **8a** with 84% selectivity, whereas the monodehydrogenation product **7a** was obtained with 16% selectivity. Besides, replacing the O_2_-enriched atmosphere with ambient air led to a lower yield (entry 3, Table 9), indicating that the reaction efficiency is strongly dependent on the oxygen concentration.

**Table 9:**
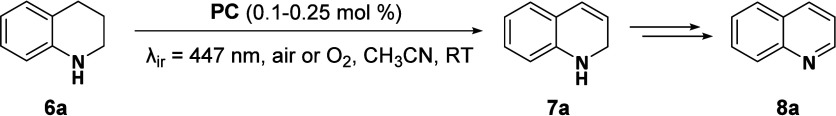
PC/Substrate Rate Screening in the Photocatalytic Dehydrogenation of 1,2,3,4-Tetrahydroquinoline (**6a**) with **1** ^a^

**entry**	**[PC]/[6a] rate**	**[PC] (%)**	**conditions**	**conversion (%)**
1	1/1000	0.1	O_2_	48
2	1/400	0.25	O_2_	99
3	1/400	0.25	air	71

a Reaction conditions: 1,2,3,4-tetrahydroquinoline (**6a**, 50 mM), PC (**1**, 0.1–0.25 mol %), acetonitrile (up to 2 mL), room temperature, under air or O_2_ (balloon, 1 atm), under irradiation with blue light (LED, λ_ir_ = 447 nm, 59.2 mW cm^–2^), during 24 h in a septum-capped tube. Conversions were experimentally determined from ^1^H NMR integration of the corresponding reaction crudes with 1,3,5-trioxane as internal standard. The conversion values were calculated as the mean of two independent experiments.

**Figure 7:**
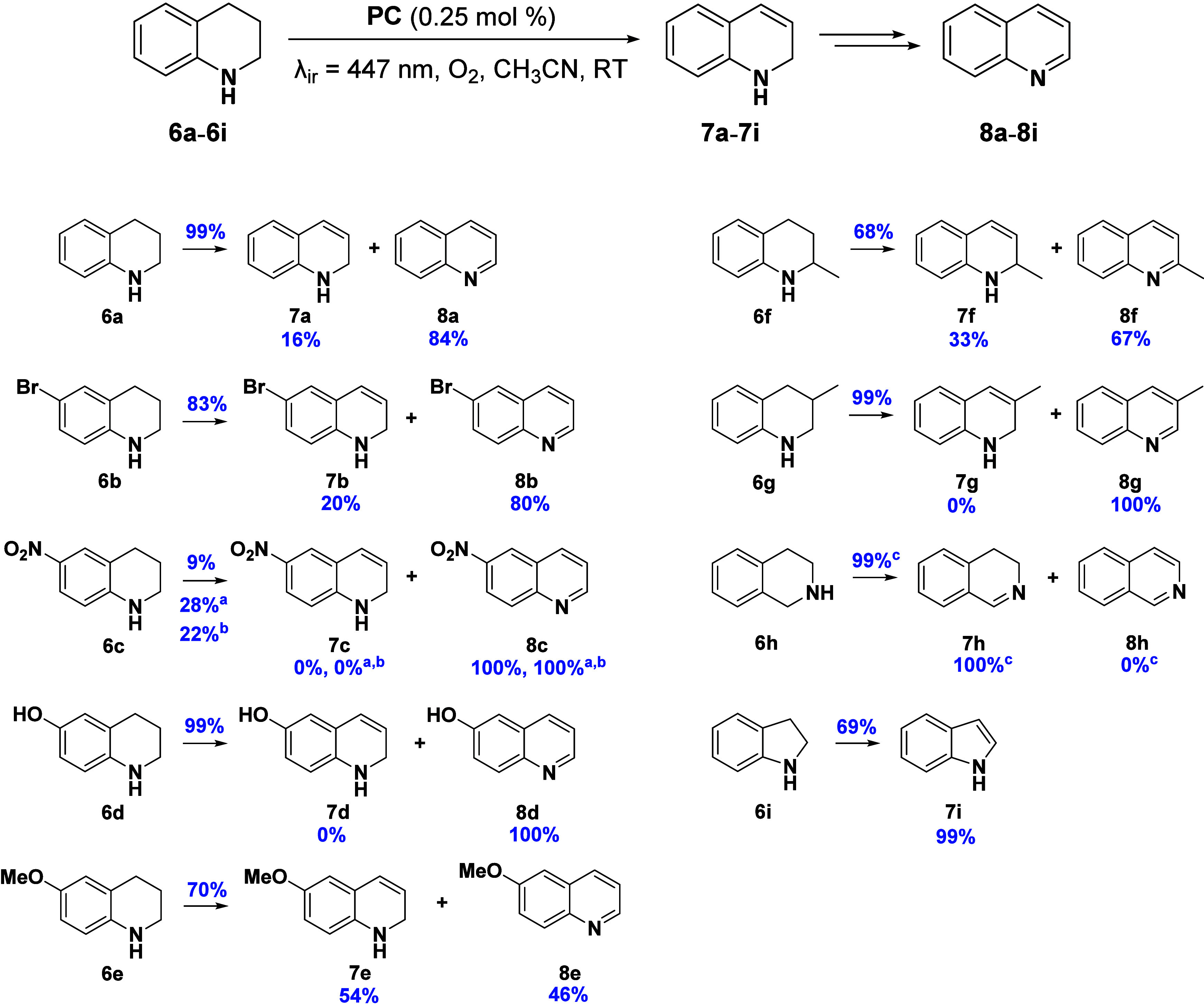
Photocatalytic oxidation of N-heterocycles **6a**–**6i**. Reaction conditions: N-heterocycle (50 mM), PC (**1**, 0.25 mol %), acetonitrile (2 mL), under O_2_ atmosphere (balloon, 1 atm), under irradiation with blue light (LED, λ_ir_ = 447 nm, 59.2 mW cm^–2^) during 24 h in a septum-capped tube. Conversions were experimentally determined from ^1^H NMR integration of the corresponding reaction crudes with 1,3,5-trioxane as internal standard. The conversion values were calculated as the mean of two independent experiments. ^a^48 h of reaction. ^b^24 h of reaction with 0.5 mol % of **1**. ^c^1 h under air conditions.

**Figure 8:**
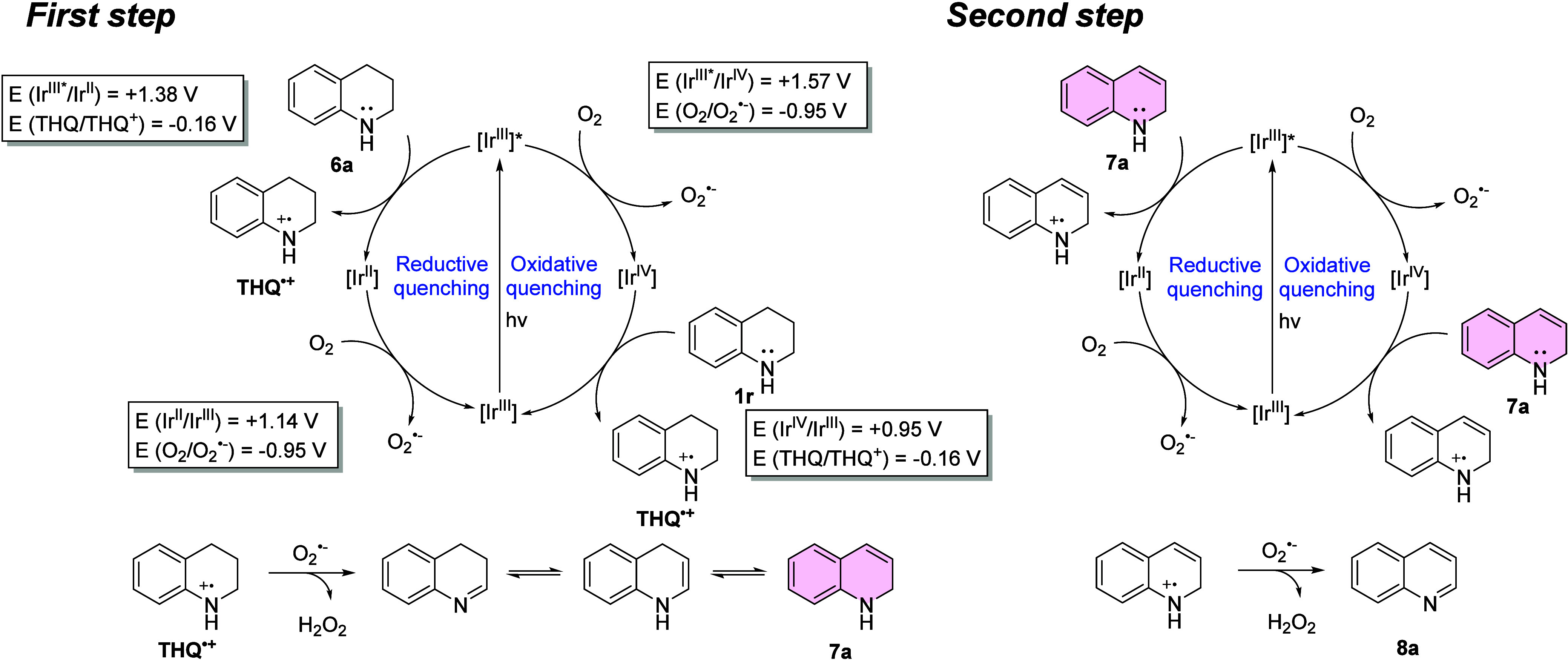
Plausible mechanism of the photocatalytic dehydrogenation of 1,2,3,4-tetrahydroquinoline (**6a**) with complex **1**.

To compare the photocatalytic activity of **1** with the rest of PCs present in this work, the reaction was also performed in the presence of the different complexes. As it can be observed in Table 10, both pbpn complexes **1** and **3** exhibited similar activities, achieving total conversion of **6a** after 24 h, while the pbpz-containing complex **2** reached only 75% conversion under the same conditions (entries 1–3, Table 10). Notably, complex **4** displayed higher activity, achieving complete conversion in just 6 h (entry 4, Table 10). Overall, these results demonstrate the exceptional performance of our system, exhibiting higher activity than previously reported half-sandwich Ir­(III) photocatalysts that required 2 mol % to perform the dehydrogenation reaction.^49^

**Table 10:** Photocatalysts Screening in the Photooxidation of 1,2,3,4-Tetrahydroquinoline (**6a**)­^a^

**entry**	**PC**	**time (h)**	**conversion (%)**
1	**1**	24	99
2	**2**	24	75
3	**3**	24	99
4	**4**	6	99
5	**5**	20	99

a Reaction conditions: 1,2,3,4-tetrahydroquinoline (**6a**, 50 mM), PC (0.25 mol %), acetonitrile (up to 2 mL), room temperature, under O_2_ (balloon, 1 atm), under irradiation with blue light (LED, λ_ir_ = 447 nm, 59.2 mW cm^–2^) in a septum-capped tube. Conversions were experimentally determined from ^1^H NMR integration of the corresponding reaction crudes with 1,3,5-trioxane as internal standard. The conversion values were calculated as the mean of two independent experiments.

The scope of the reaction was first expanded using other N-heterocycles derived from 1,2,3,4-tetrahydroquinole with different substituents in 6-position (Figure 7). Good results were obtained when 6-bromo-1,2,3,4-tetrahydroquinoline (**6b**) was studied, obtaining 83% conversion within 24 h with an 80% selectivity to the corresponding quinoline (**8b**). However, the presence of the stronger electron-withdrawing nitro group in **6c** dramatically reduced the yield to 9%. Longer reaction times or higher PC loadings were needed for increasing the yield. Interestingly, despite the moderate conversions obtained, 100% selectivity to 6-nitroquinoline (**8c**) was observed in all cases. Better results were obtained when electron-donating groups were used, such as hydroxy (**6d**) and methoxy (**6e**). Whereas 6-hydroxyquinoline (**8d**) was obtained in 100% selectivity, a mixture of 6-methoxy-1,2-dihydroquinoline (**7e**) and 6-methoxyquinoline (**8e**) was obtained.

Next, 2-methyl- (**6f**) and 3-methyl-1,2,3,4-tetrahydroquinoline (**6g**) were also evaluated. When the substrate was substituted in 3-position, complete conversion was obtained within 24 h with 100% selectivity to the corresponding quinoline (**8g**). In contrast, the substitution in the 2-position slightly reduced the conversion to 68%, producing a mixture of the corresponding dihydroquinoline (**7f**) and quinoline (**8f**).

Finally, the scope of the reaction was further expanded using other N-heterocycles, such as 1,2,3,4-tetrahydroisoquinoline (**6h**) and indoline (**6i**). It is worth noting that **1** exhibited excellent activity in the photocatalytic dehydrogenation of **6h**, achieving the total and selective conversion to 3,4-dihydroisoquinoline (**7h**) within just 1 h of reaction under air conditions. Indoline (**6i**) was also dehydrogenated to indole (**7i**) in a selective manner, although the reaction did not proceed as fast as with 1,2,3,4-tetrahydroquinoline (**6a**), achieving a 69% yield of **7i** after 24 h.

The catalytic performance of PC **1** was further compared to previously reported photocatalytic systems for the dehydrogenation of N-heterocycles. As summarized in Table S3, PC **1** efficiently promotes the dehydrogenation of 1,2,3,4-tetrahydroquinoline (**6a**) to quinoline (**8a**) under mild conditions and low PC loading. Remarkably, it displays higher activity than several reported PCs and catalytic performance comparable to that of bis-cyclometalated Ir­(III) complexes.

Afterward, a series of control experiments was conducted using PC **1** to gain insight into the plausible reaction mechanism. The reaction was performed under three different conditions, including in the absence of light, oxygen, or photocatalyst (entries 3–5, Table 11). As expected, no conversion was observed in the absence of either light or photocatalyst. When the reaction was conducted under ambient air, incomplete conversion was obtained, whereas under a nitrogen atmosphere, no reaction occurred. These observations indicate that oxygen plays a crucial role in the transformation and that a sufficiently high oxygen concentration is required for efficient conversion. The reaction was also evaluated under purely thermal conditions (65 °C) in the absence of light (entry 6, Table 11). No reaction was detected under these conditions, confirming that the process is not thermally driven.

**Table 11:** Control Experiments for the Oxidative Dehydrogenation of 1,2,3,4-Tetrahydroquinoline (**6a**)­^a^

**entry**	**conditions**	**conversion (%)**
1	PC, **O**_**2**_, light	99
2	PC, **air**, light	71
3	PC, O_2_, **no light**	0
4	**no PC**, O_2_, light	0
5	PC, **N**_**2**_, light	2
6	PC, air, **65 °C**, **no light**	0
7	PC, O_2_, light, **DABCO** ^b^	99
8	PC, O_2_, light, **TEMPO** ^b^	59
9	PC, O_2_, light, **BQ** ^b^	44
10	PC, O_2_, light, *t* **BuOH** ^b^	99

a Reaction conditions: 1,2,3,4-tetrahydroquinoline (**6a**, 50 mM), PC (**1**, 0.25 mol %), acetonitrile (up to 2 mL), room temperature, under air or a saturated atmosphere of either O_2_ or N_2_ (balloon, 1 atm), under irradiation with blue light (LED, λ_ir_ = 447 nm, 59.2 mW cm^–2^) during 24 h in a septum-capped tube. Conversions were experimentally determined from ^1^H NMR integration of the corresponding reaction crudes with 1,3,5-trioxane as internal standard. The conversion values were calculated as the mean of two independent experiments.

b 11 mM of DABCO, TEMPO, 1,4-benzoquinone (BQ) or *tert*-butanol (*t*BuOH) scavengers.

Differently to the previous processes, when the reaction was performed in the presence of DABCO, no decrease in the activity was observed (entry 7, Table 11), indicating that ^1^O_2_ is not involved in the mechanism. Furthermore, experiments carried out in the presence of TEMPO and BQ showed a partial inhibition of the reaction, leading to 59 and 44% conversion, respectively (entries 8–9, Table 11). These results suggest the participation of some radical species, such as O_2_^•–^. When *t*BuOH was employed as scavenger, no decrease in activity was detected (entry 10, Table 11), indicating that HO^•^ radicals do not participate in the reaction. Additionally, H_2_O_2_ was detected in the reaction crude mixture by ^1^H NMR as a broad signal at around 8.5–8.7 ppm, and its presence was further confirmed using test strips.

Based on our results and consistent with previous reports on Ir­(III) photosensitizers in the photocatalytic dehydrogenation of tetrahydroquinolines,^27^ a plausible reaction mechanism is proposed (Figure 8). In this reaction, only the electron transfer mechanism is operative, which may proceed via either a reductive or an oxidative quenching pathway, based on the potential values. Upon light excitation, the PC reaches its triplet excited state and undergoes reductive quenching to generate an Ir­(II) species, while substrate **6a** is simultaneously oxidized to radical cation intermediate THQ^•+^. Concurrently, molecular oxygen is reduced to produce superoxide radicals O_2_^•–^. Alternatively, through an oxidative quenching pathway, the excited triplet state of the PC can efficiently transfer an electron to oxygen, generating O_2_^•–^, with **6a** simultaneously oxidizing to THQ^•+^. In both mechanistic pathways, the resulting THQ^•+^ species further reacts to afford the corresponding cyclic imine, accompanied by the formation of H_2_O_2_. The initially formed imine undergoes a rearrangement from 1,2-dihydroquinoline to 3,4-dihydroquinoline (**7a**), which is then further oxidized in a subsequent dehydrogenation cycle to yield the fully aromatic quinoline product (**8a**).

Finally, Stern–Volmer quenching experiments were performed by using substrate **6a**. In line with the previous studies, the emission of PC **1** (10 μM) was progressively quenched upon addition of increasing concentrations of **6a** (0–10 equiv) (Figure S17), indicating quenching of the excited state of PC by the substrate. In this case, the Stern–Volmer quenching constant was determined to be *K*_SV_ = 4.1 ×10^3^ M^–1^.

### 3.5 Reusability

Once demonstrated the outstanding activity of photocatalyst **1** in all the reactions performed, we decided to test its reusability in the photocatalytic oxidative coupling of benzylamine (**1a**) to the corresponding imine product (**2a**). The experiment was performed on a larger scale (5 mL final volume), and the reaction time was fixed to 8 h. Notably, an exceptionally low photocatalyst-to-substrate ratio of 1/2000 (0.05 mol %) was employed, highlighting the remarkable efficiency of the system (Figure 9). Despite this minimal catalyst loading, the reaction proceeded quantitatively over the first five cycles, demonstrating both high activity and robustness, while a decrease in conversion was observed in the sixth cycle. These results underscore the excellent performance of catalyst **1** even at very low concentrations, pointing to its high versatility for photocatalytic transformations. The decrease in activity could be attributed to photodegradation of the complex under irradiation, as supported by the photostability assays.

**Figure 9:**
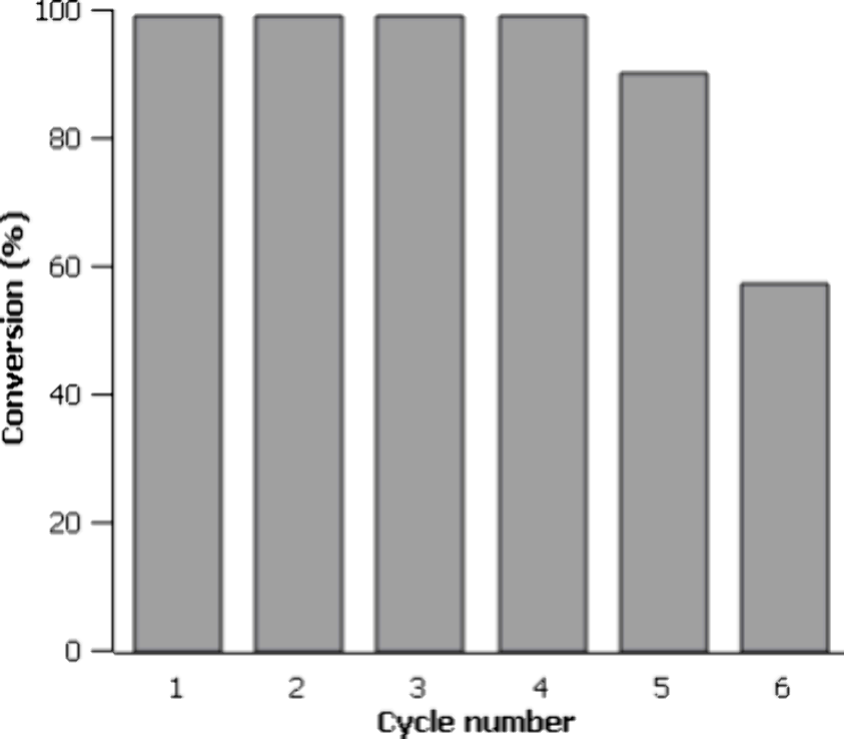
Conversion of the photocatalytic oxidative coupling of benzylamine (**1a**) with **1** (0.05 mol %). Reaction conditions: benzylamine (**1a**, 50 mM), PC (**1**, 0.05 mol %), acetonitrile (up to 5 mL), room temperature, under air and irradiation with blue light (LED, λ_ir_ = 447 nm, 59.2 mW cm^–2^), in a septum-capped tube. Conversions were experimentally determined from ^1^H NMR integration of the corresponding reaction crudes with 1,3,5-trioxane as internal standard.

## 4 Conclusions

In summary, we demonstrate that half-sandwich Ir­(III) complexes bearing π-extended ligands (pbpn and pbpz) can be efficiently employed as photocatalysts in a broad range of synthetically relevant organic transformations, including the oxidative coupling of amines and the oxidation of thioethers. Although octahedral Ir complexes have previously been reported for the photooxidation of amines and thioethers, to the best of our knowledge, this study represents the first example of a piano-stool Ir­(III) complex exhibiting such reactivity in these reactions. These complexes also display high activity in the dehydrogenation of N-heterocycles. These results highlight the expanded catalytic potential of this family of Ir complexes in photocatalytic organic transformations.

Electrochemical analysis indicated that the Ir complexes act as potent photooxidants and photoreductants from their excited states, highlighting their dual-redox activity under photoexcitation. Furthermore, the photoredox potential displayed by these Cp*Ir complexes was higher than other photocatalytic systems reported previously, suggesting an enhanced capacity to engage in challenging redox transformations.

Complex **1** catalyzed these organic transformations with a broad substrate scope and excellent chemoselectivity under mild conditions, including room temperature and ambient air or an oxygen atmosphere as green oxidants. Quantitative conversions were achieved at very low catalyst loadings (0.005 mol %). In general, electron-donating substituents showed a positive effect on the catalytic rates. It is worth noting that high selectivity toward sulfoxide formation was observed in most thioether oxidation reactions tested. The plausible mechanisms for these reactions were investigated, indicating that some ROS such as ^1^O_2_ and O_2_^•‑–^ play a major role in these light-driven transformations. Importantly, the results also demonstrate a clear structure–property relationship in which π-extension of the cyclometalating ligand strongly influences the electronic structure and excited-state properties of the resulting Ir­(III) complexes, ultimately leading to enhanced photocatalytic activity in the pbpn-containing derivatives.

The catalytic activity was also investigated under environmentally relevant conditions, including an aqueous solvent mixture (MeCN/H_2_O 1:1) or sunlight irradiation, exhibiting outstanding conversions. Furthermore, complex **1** maintained high activity over at least five consecutive cycles, supporting its stability and reusability. Overall, these results establish these Cp*Ir­(III) complexes as highly efficient, versatile, and robust photocatalysts, providing a valuable platform for the development of sustainable methodologies in organic synthesis.

## Supplementary Material



## Notes

^1^H NMR spectra of photostability studies; superoxide anion radical generation experiments; UV–vis absorption and emission spectra; photocatalysis mechanism schemes; emission-quenching experiments; NMR characterization of photocatalytic products; comparison tables with other photocatalysts (PDF)

## Abbreviations

BQ: 1,4-Benzoquinone
bzIm: *N*-benzylimidazole
bzNH_2_: Benzylamine
Cp*: 1,2,3,4,5-Pentamethylcyclopentadienyl
CV: Cyclic voltammetry
DABCO: 1,4-Diazabicyclo­[2.2.2]­octane
DHR123: Dihydrorhodamine 123
DMB: 1,4-Dimethoxybenzene
DMPO: 5,5-Dimethyl-1-pyrroline-*N*-oxide
EPR: Electron-paramagnetic resonance
GC: Glassy carbon
HO^•^: Hydroxyl radical
HOMO: Highest occupied molecular orbital
H_2_O_2_: Hydrogen peroxide
ISC: Intersystem crossing
LUMO: Lowest occupied molecular orbital
^1^O_2_: Singlet oxygen
O_2_^•–^: Superoxide radical anion
pbpn: 4,9,16-Triazadibenzo­[*a*,*c*]­naphthacene
pbpz: 4,9,14-Triazadibenzo­[*a*,*c*]­anthracene
PC: Photocatalyst
PDT: Photodynamic therapy
PS: Photosensitizer
Q: Quencher
ROS: Reactive oxygen species
R_2_S^•+^: Thioether radical cation
*t*BuOH: *tert*-Butanol
TD-DFT: Time-dependent density functional theory
TEMP: 2,2,6,6-Tetramethylpyridine
TEMPO: 2,2,6,6-Tetramethylpiperidine-*N*-oxide
TEMPO: 2,2,6,6-Tetramethylpiperidine 1-oxyl
THQ: 1,2,3,4-Tetrahydroquinoline
