# Supplementary material for: Multifunctional Photocatalysts Based on Half-Sandwich Cyclometalated Ir(III) Complexes with π‑Extended Ligands
Source: Inorg Chem. 2026 Apr 3;65(15):8611–27. doi: 10.1021/acs.inorgchem.6c00584 (PMC13100946; doi:10.1021/acs.inorgchem.6c00584)
Supplement: Supplementary file 1 [file ic6c00584_si_001.pdf]

Supporting Information

Multifunctional Photocatalysts Based on Half-Sandwich Cyclometalated Ir(III) Complexes with π-Extended Ligands

Carlos Gonzalo-Navarro,[1,2] Melania Sánchez,[1] Gregorio Castañeda,[3] Pedro Tavares,[4,5] Blanca R. Manzano,[1,2] Gema Durá,[1,2]*

1 Universidad de Castilla-La Mancha, Departamento de Q. Inorgánica, Orgánica y Bioquímica, Facultad de Ciencias y Tecnologías Químicas, Avda. Camilo José Cela, 10, 13071 Ciudad Real, Spain.

2 Instituto Regional de Investigación Científica Aplicada (IRICA), UCLM, Avda. Camilo José Cela, Ciudad Real, 13071, Spain

3 Departamento de Q. Analítica y Tecnología de los Alimentos, Facultad de Ciencias y Tecnologías Químicas, UCLM, Avda. Camilo José Cela, 10, 13071 Ciudad Real, Spain.

4 Associate Laboratory i4HB - Institute for Health and Bioeconomy, NOVA School of Science and Technology, Universidade NOVA de Lisboa, 2829-516 Caparica, Portugal.

5 UCIBIO – Applied Molecular Biosciences Unit, Department of Chemistry, NOVA School of Science and Technology, Universidade NOVA de Lisboa, 2829-516 Caparica, Portugal

Email: gema.dura@uclm.es

Irradiation system. Light irradiation was performed in a 16-compartment 'Medusa' photo-multireactor built by Microbeam, equipped with high-power LEDs (2.3 W, 700 mA, Luxeon Rebel, Philips Lumileds). The LEDs are situated below each compartment (Figure S1). Every reactor position is composed of 40 mL cylindrical glass flask provided by Scharlau. All reaction positions were irradiated from the bottom using monochromatic light provided by LEDs of blue light (470 ± 20 nm, 51.4 mW cm^{-2}). All reactions in the system could be stirred using an orbital stirrer, which was set at 50 rpm.

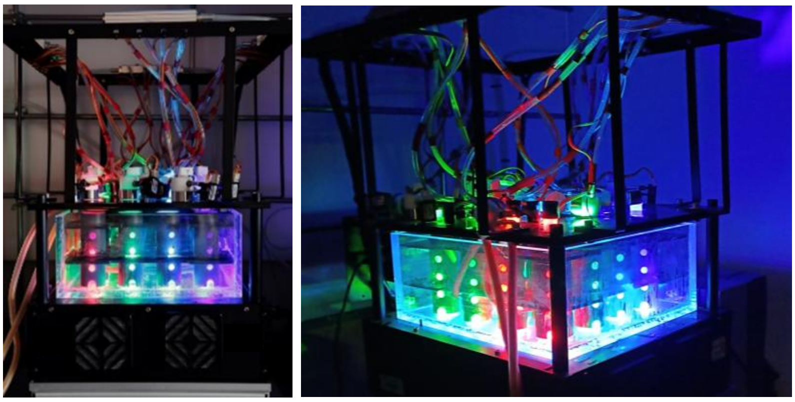

Figure S1. 'Medusa' photoreactor used for the photostability and photocatalysis studies.

Photostability experiments.

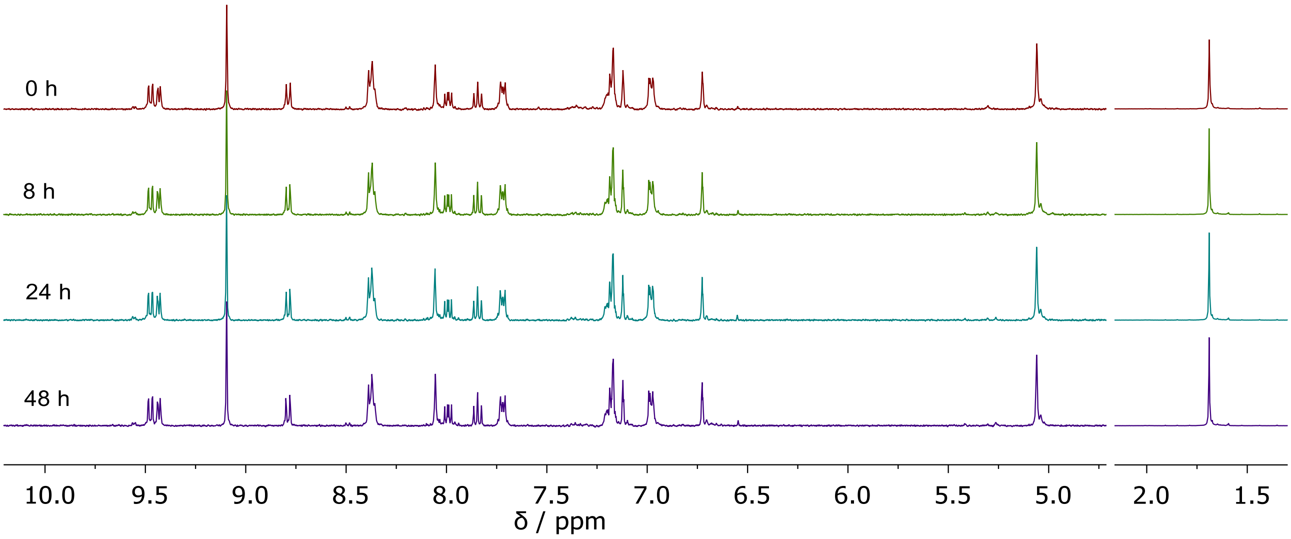

Figure S2. ^{1}H NMR of complex **1** in DMSO-d_6 at different times in the dark.

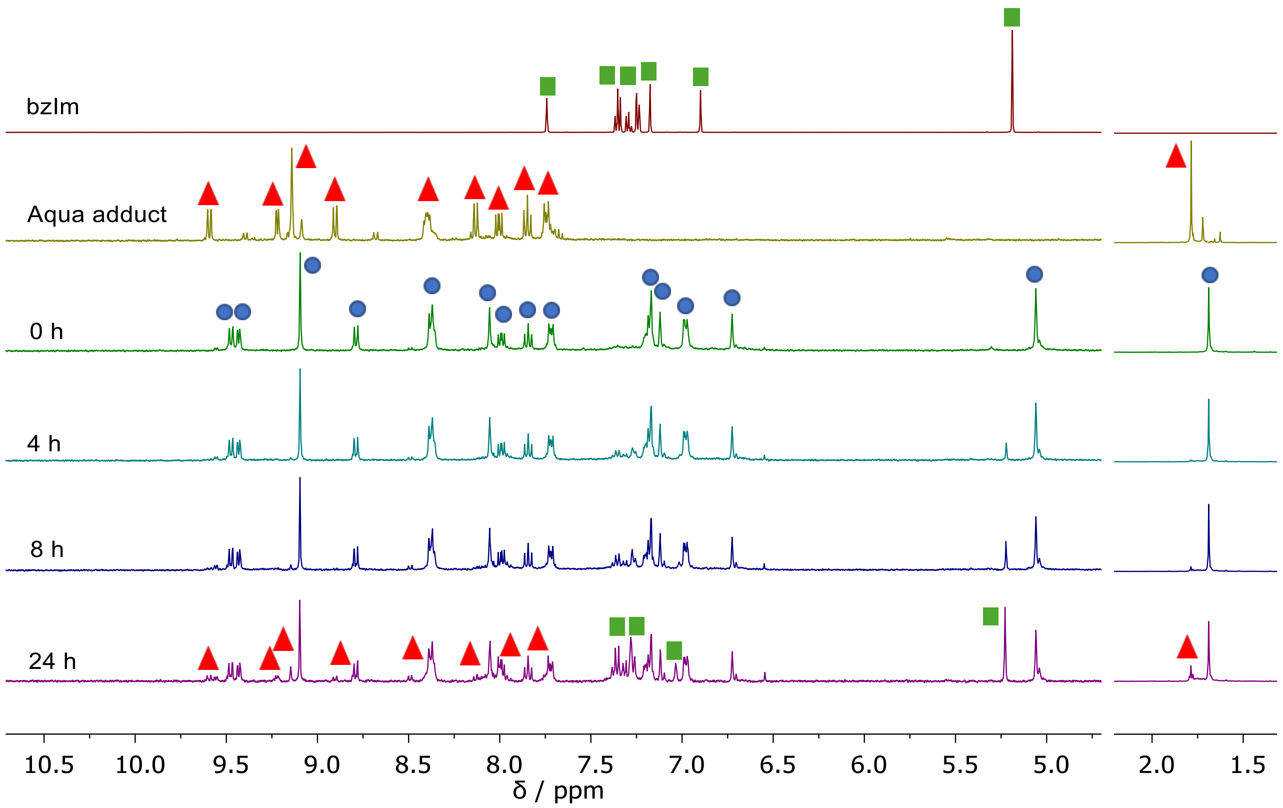

Figure S3. ^{1}H NMR of complex **1** in DMSO-d_6 at different times under blue light irradiation (447 nm, 59.2 mW cm^{-2}). Blue circles (•) correspond to complex **1**, red triangles (▲) correspond to the aqua adduct **1w**, and green squares (■) correspond to the free bzIm ligand.

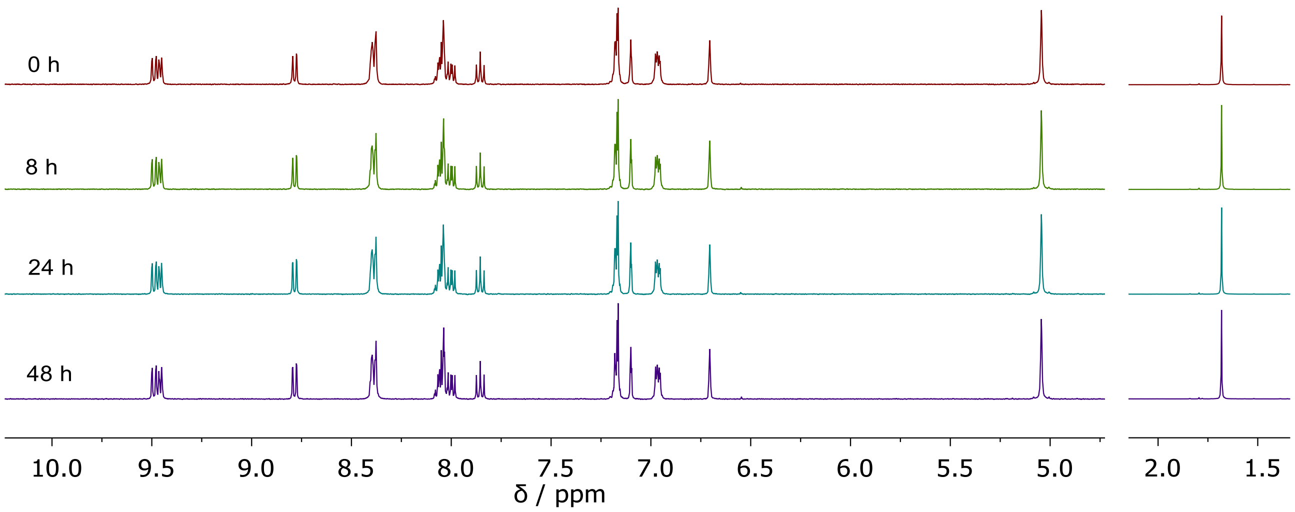

Figure S4. ^{1}H NMR of complex **2** in DMSO-d_6 at different times in the dark.

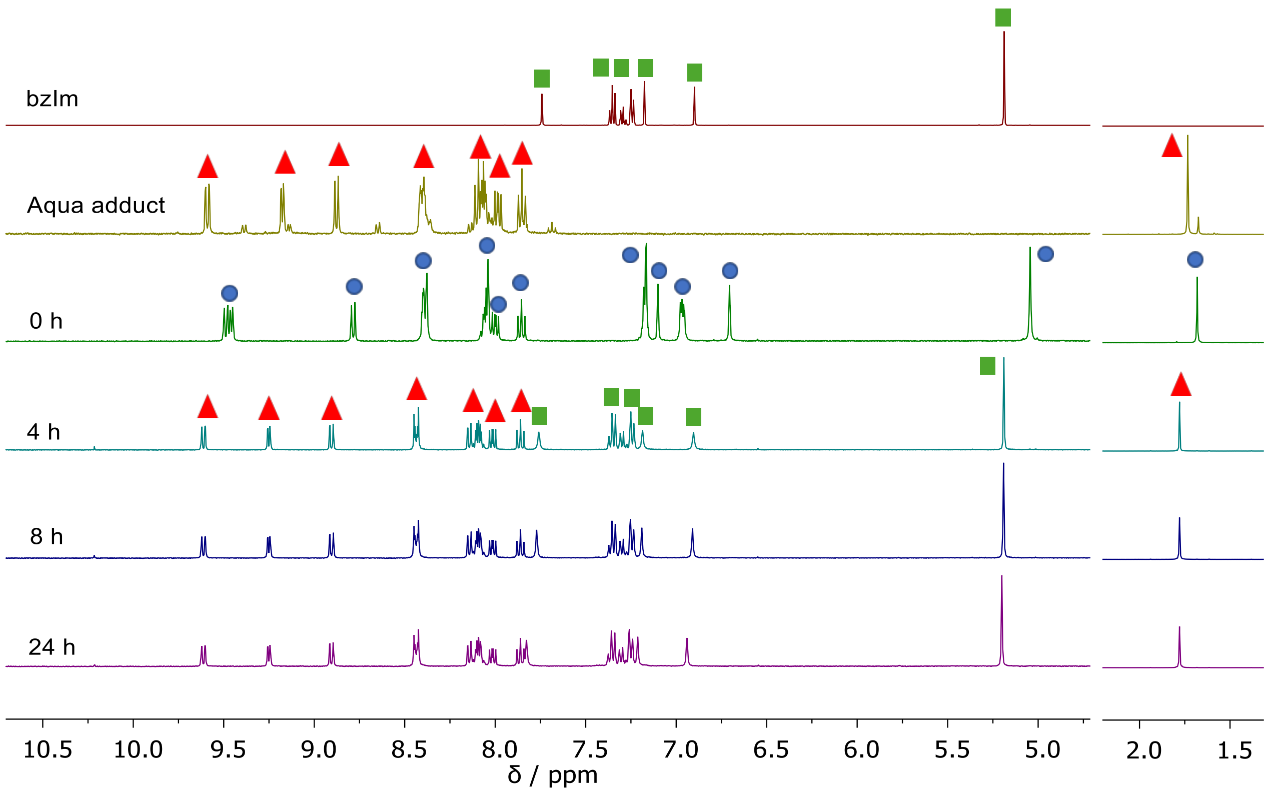

Figure S5. ^{1}H NMR of complex **2** in DMSO-d_6 at different times under blue light irradiation (447 nm, 59.2 mW cm^{-2}). Blue circles (•) correspond to complex **2**, red triangles (▲) correspond to the aqua adduct **2w**, and green squares (■) correspond to the free bzIm ligand.

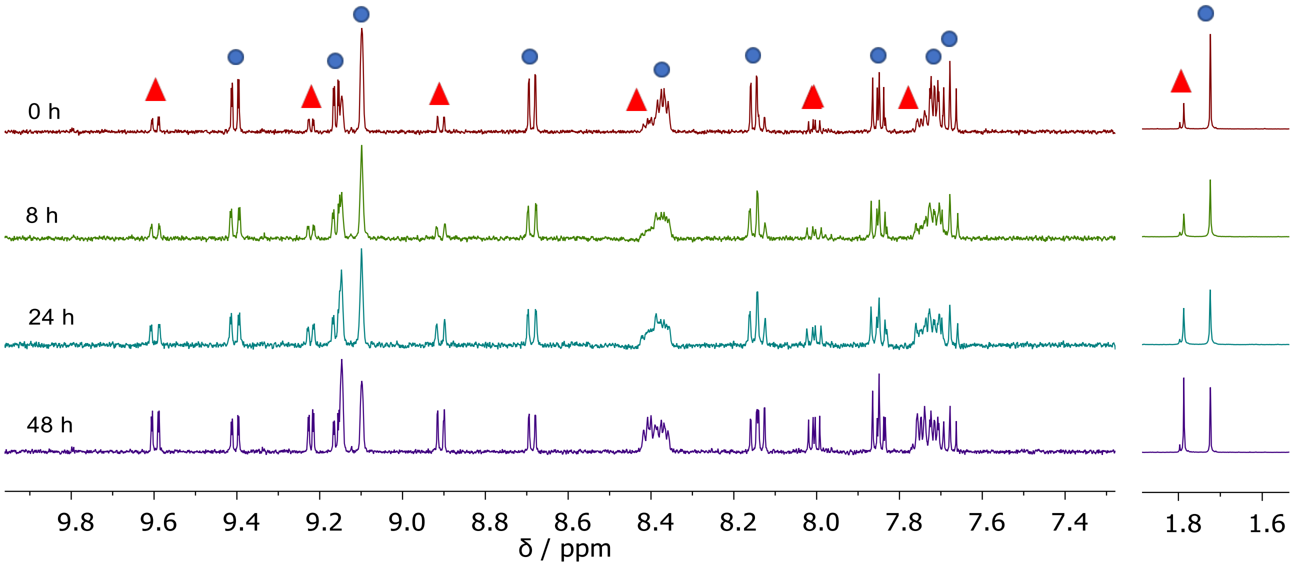

Figure S6. ^{1}H NMR of complex **3** in DMSO-d_6 at different times in the dark. Blue signals (•) correspond to chlorido complex **3**, red triangles (▲) correspond to the aqua adduct **1w**.

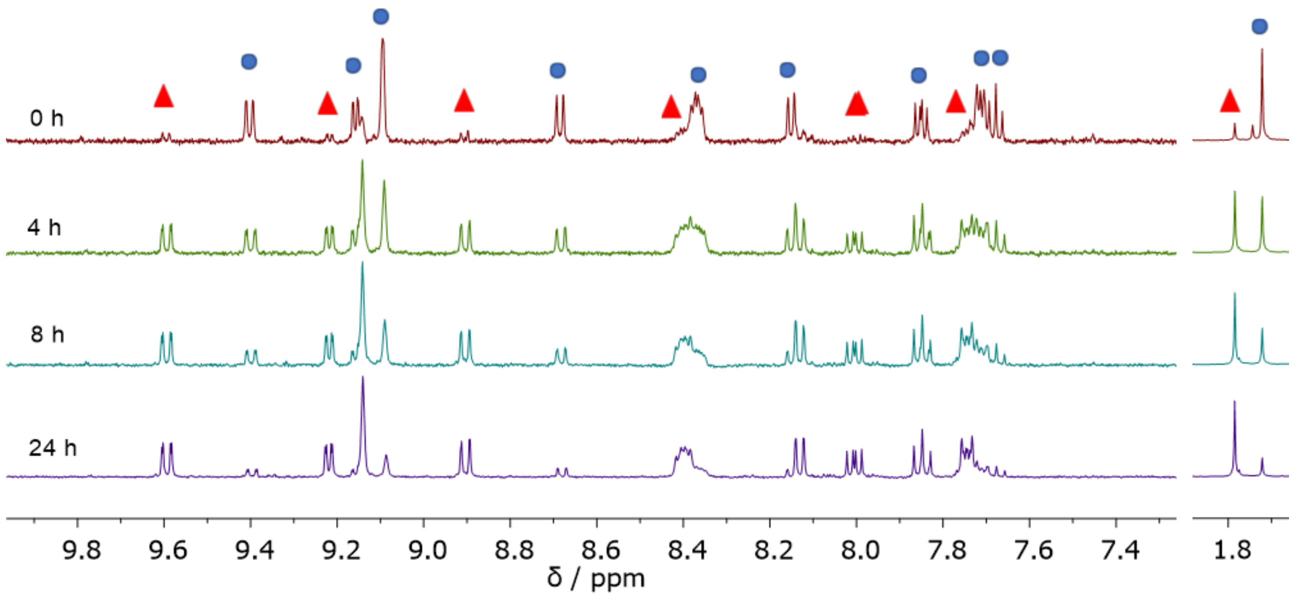

Figure S7. ^{1}H NMR of complex **3** in DMSO-d_6 at different times under blue light irradiation (447 nm, 59.2 mW cm^{-2}). Blue signals (•) correspond to chlorido complex **3**, red triangles (▲) correspond to the aqua adduct **1w**.

Superoxide anion radical ($O_2^{\bullet-}$) generation.

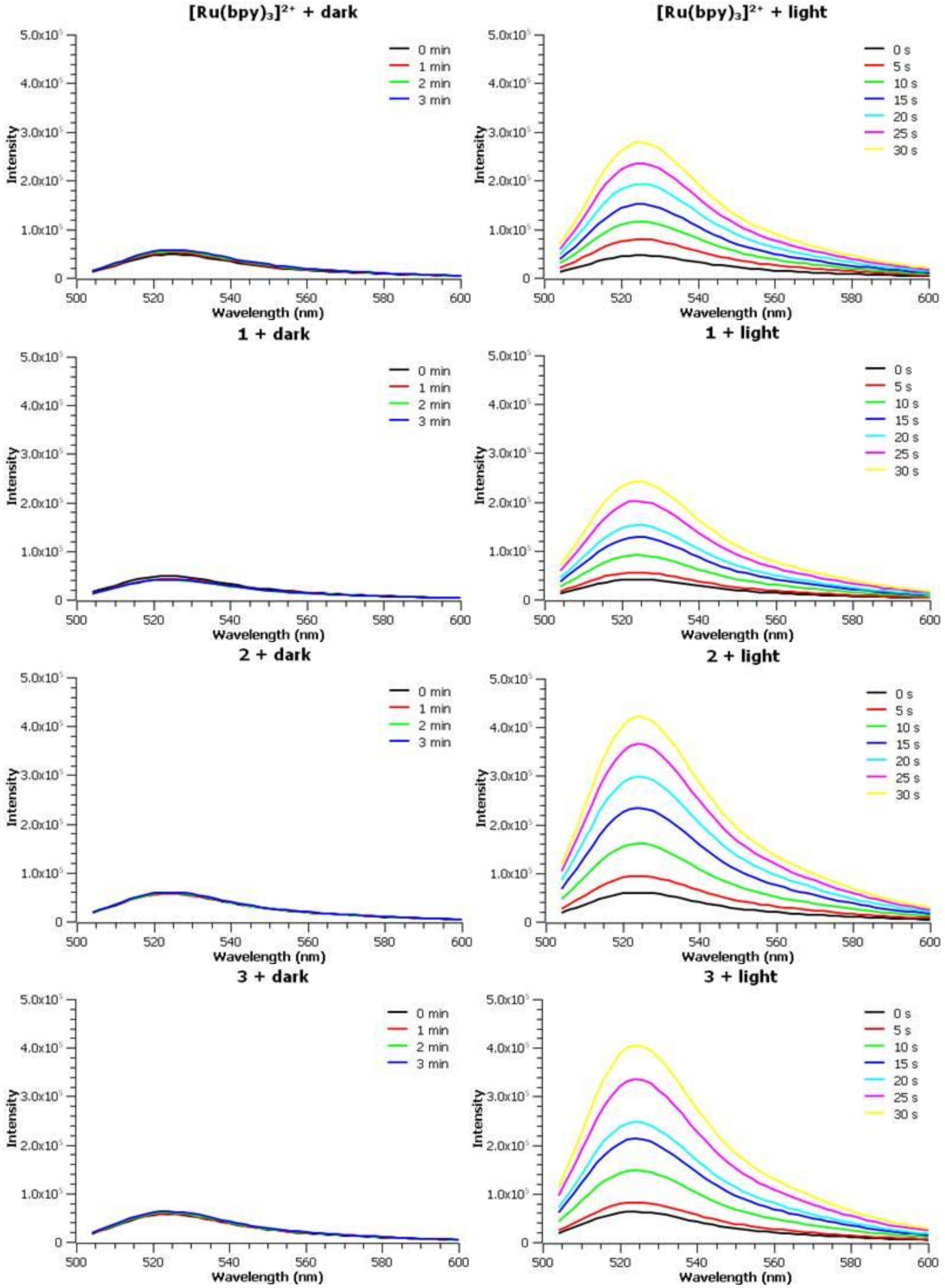

Figure S8. Evolution of the fluorescence spectra emission of DHR123 in the presence of the photosensitizers in dark and under blue light irradiation (470 nm, 51.4 mW cm^{-2}) in water (0.2% DMSO).

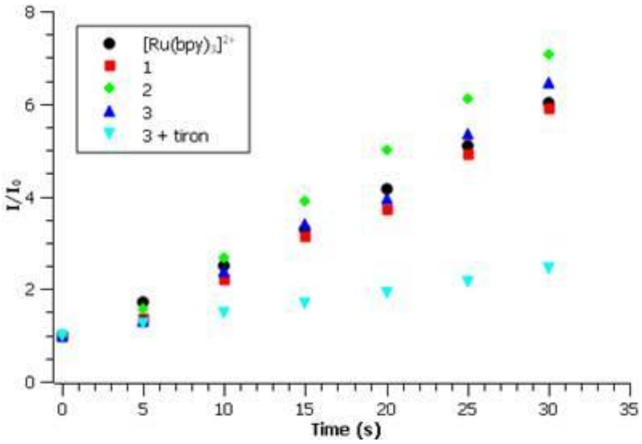

Figure S9. Comparative plots of I/I_0 as a function of time for the superoxide photogeneration in presence of the photosensitizers upon blue light irradiation (470 nm, 51.4 mW cm^{-2}) in water (0.2% DMSO).

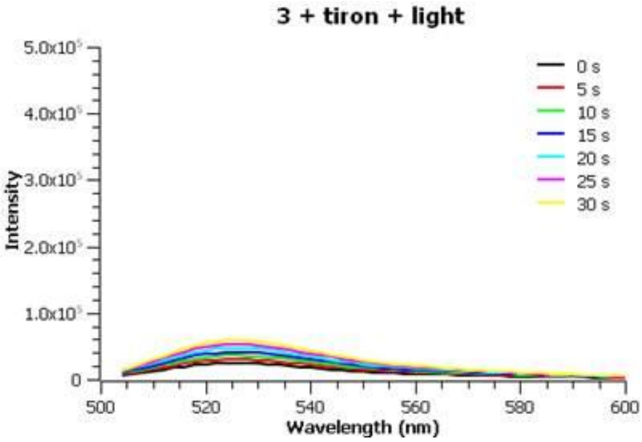

Figure S10. Evolution of the fluorescence spectra emission of DHR123 in the presence of complex **3** with tiron as superoxide scavenger under blue light irradiation (470 nm, 51.4 mW cm^{-2}) in water (0.2% DMSO).

EPR measurements.

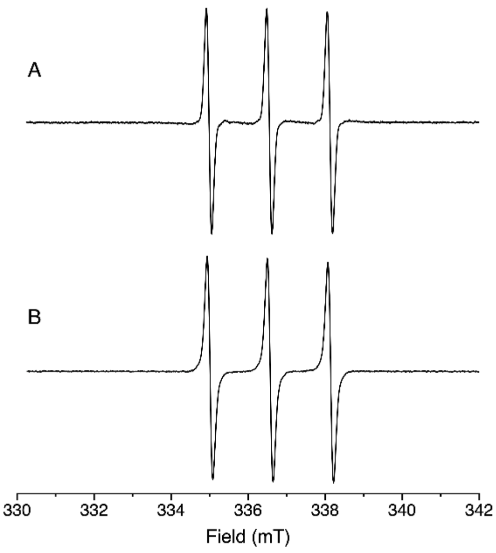

Figure S11. EPR spectra of irradiated samples of complexes **2** (A) and **1** (B) in acetonitrile in the presence of TEMP. Acquisition parameters were as follows: single scan (60 s); gain, 100; modulation amplitude, 0.1 mT; microwave power, 10 mW; microwave frequency, 9.45 GHz. Spectra can be described by a calculated *g* value of 2.0055 and A_N value of 1.57 mT.

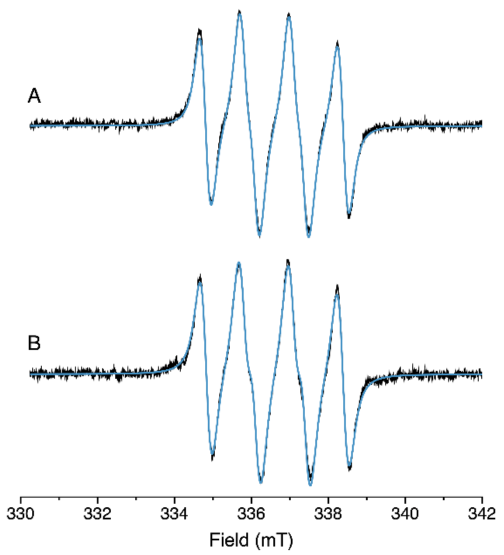

Figure S12. EPR spectra of irradiated samples of complexes **2** (A) and **1** (B) in acetonitrile in the presence of DMPO. Acquisition parameters were as follows: four scans (60 s); gain, 500; modulation amplitude, 0.2 mT; microwave power, 50 mW; microwave frequency, 9.45 GHz. Blue lines below the spectra represent simulations obtained using EasySpin (easyspin.org) [1] assuming isotropic spin systems with the following parameters: g = 2.0054, A_H = 1.00 mT, A_N = 1.25 mT.

UV-vis absorption and emission measurements.

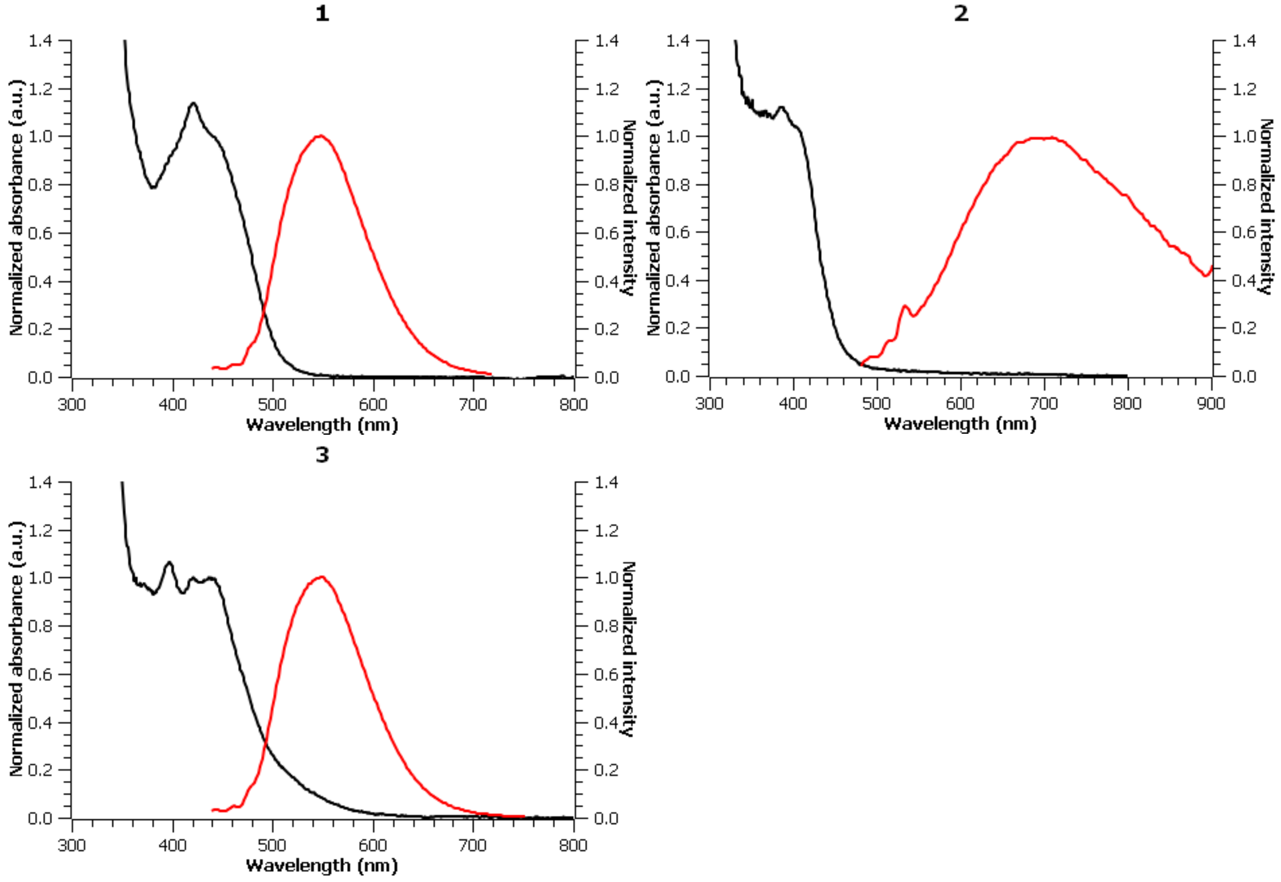

Figure S13. Normalized UV-vis absorption and emission spectra of complexes **1** and **3** in acetonitrile (10^{-5} M). Absorption spectra were normalized to the lowest-energy band. The intersection points were used to estimate the excited state potentials according to the Weller approximation.[2,3]

Photocatalysis.

Photocatalytic oxidation of benzylamine and other amines.

Table S1. Comparison of representative photocatalysts with PC **1** for the oxidative coupling of primary amines.

PC	Substrate	PC loading	Irradiation system	Reaction conditions	Conv. (%)	Ref.
MIL-125-NH–CH_2OH@Ag-0.5@COF-2	$bzNH_2$	10 mg / 0.25 mmol substrate	Visible light	Air, 60 °C, 12 h, MeCN	100	a
$[Ru(bpy)_2(N\^N)]^+$ N^N = 2-pyridylbenzimidazole derivative	$bzNH_2$	1.0 mol %	Blue LED (460 nm)	O_2, RT, 30 min, MeCN	>99	b

4-NA-Cu_2O RDs<br>NA = 4-nitrophenylacetylene<br>RDs = rhombic dodecahedra	$bzNH_2$	3 mg / 1 mmol substrate	40 W UV LED (370 nm)	O_2, RT, 30 min, MeCN	98	c
$[Ru(N^\wedge N)_3]_4$–⊂N+-COF<br>N^N = 2,2'-bipyridine-4,4'-dicarboxylate	$bzNH_2$	1 mg / 0.2 mmol substrate	450 nm	O_2, RT, 3 h, MeCN	100	d
$[Ru(N^\wedge N^\wedge N)_2]^{2+}$<br>N^N^N = terpy-R motif within porous poycarbazole	$bzNH_2$	0.37 mol %	23 W white LED	O_2, RT, 8 h, MeCN	99	e
$[(C^\wedge N)_2Ir(Se^\wedge Se)]^+$<br>C^N = 2-(2′-benzothienyl)pyridinato<br>Se^Se = bis(imidazoline selone)	$bzNH_2$	0.25 mol %	Blue LED (460 nm, 0.9 mW cm^{-2})	O_2, RT, 5 h, DCM	94	f
$[(C^\wedge N)Ir(N^\wedge N^\wedge N)Cl]^+$<br>C^N = 2-(4-methylphenyl)pyridine<br>N^N^N = 2,6-bis(imidazo[1,5-*a*]pyridin-3-yl)pyridine	$bzNH_2$	0.15 mol %	1.1 W blue LED (465 nm)	O_2, RT, 3 h, MeCN, 3Å MS	97	g
$[Ru(bpy)_2(N^\wedge N)]^+$<br>N^N = N-(arylsulfonyl)-8-amidoquinolate derivative	$bzNH_2$	0.1 mol %	24 W blue LED (460 nm)	O_2, RT, 14 h, MeCN	>99	h
$K_6H[\{Ru_2Cl(H_2O)(CH_3COO)_2\}\{WO(H_2O)\}_2(PW_9O_{34})2]\cdot 14H_2O$	$bzNH_2$	0.1 mol %	10 W blue LED	O_2, RT, 24 h, neat	97	i
$[(ppy)_2Ir(N^\wedge N)]$<br>N^N = dipyrrinato derivative	$bzNH_2$	0.05 mol %	White light (96 mW cm^{-2})	O_2, RT, 2 h, MeCN	97	j
**1**	$bzNH_2$	0.005 mol %	2.3 W blue LED (447 nm, 59.2 mW cm^{-2})	Air, RT, 8 h, MeCN	99	This work
FJI-Y10	$bzNH_2$	0.002 mol %	300 W visible light	O_2, 40 °C, 6 h, DMF	100	k
$Ru(bpy)_3[Mo_6O_{19}]$	$bzNH_2$	0.0006 mol %	10 W blue LED (445 nm)	Air, RT, 25 min, MeCN	100	l

a. X. Li, K. Zhang, X. Huang, Z. Wu, D. Zhao, G. Wang, *Nanoscale* **2021**, *13*, 19671–19681.
b. I. Echevarría, M. Vaquero, R. Quesada, G. Espino, *Inorg. Chem. Front.* **2020**, *7*, 3092–3105.
c. E. Wu, M. H. Huang, *ACS Catal.* **2023**, *13*, 14746−14752.
d. Y. Wang, Y. Wang, J. Li, Y. Yu, S. Huang, G. Yang, *Dalton Trans.* **2023**, *52*, 14100–14109.
e. D. A. Anito, T. Wang, H. Liang, X. Ding, B. Han, *Polym. Chem.* **2021**, *12*, 4557–4564.
f. J. Jin, H. Shin, J. H. Park, J. H. Park, E. Kim, T. K. Ahn, D. H. Ryu, S. U. Son, *Organometallics* **2013**, *32*, 3954−3959.
g. F. Yagishita, T. Nagamori, S. Shimokawa, K. Hoshi, Y. Yoshida, Y. Imada, Y. Kawamura, *Tetrahedron Letters* **2020**, *61*, 151782.
h. C. Yagüe, I. Echevarría, M. Vaquero, J. Fidalgo, A. Carbayo, F. A. Jalón, J. C. Lima, A. J. Moro, B. R. Manzano, G. Espino, *Chem. Eur. J.* **2020**, *26*, 12219 – 12232.
i. W. Chen, H. Li, J. Song, Y. Zhao, P. Ma, J. Niu, J. Wang, *Inorg. Chem.* **2022**, *61*, 2076−2085.
j. S. Kumar, L. Devi, I. Gupta, *New J. Chem.* **2025**, *49*, 11080–11088.
k. F. Zhao, G. Zhang, Z. Ju, Y. Tan, D. Yuan, *Inorg. Chem.* **2020**, *59*, 3297−3303.
l. H. An, H. Luo, T. Xu, S. Chang, Y. Chen, Q. Zhu, Y. Huang, H. Tan, Y. Li, *Inorg. Chem.* **2022**, *61*, 10442−10453.

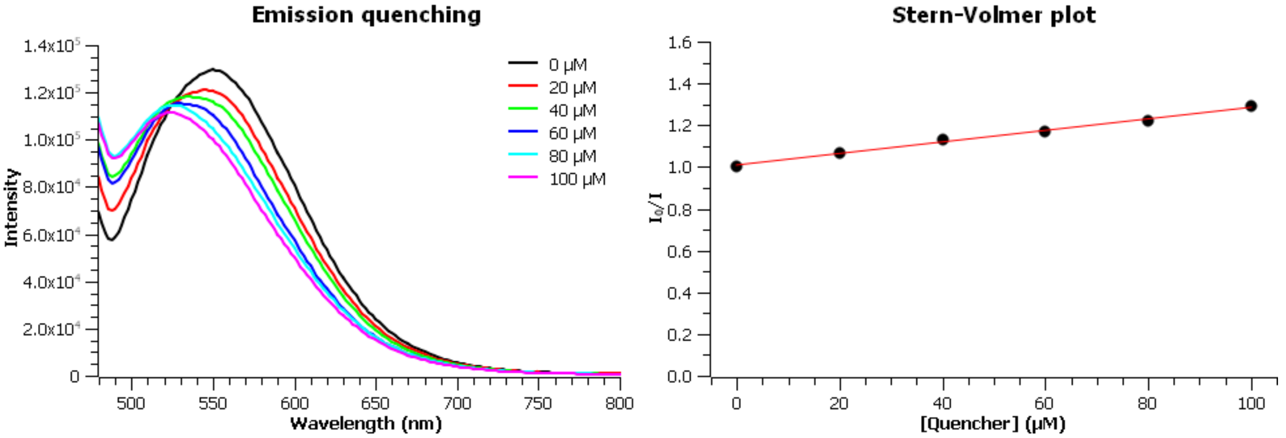

Figure S14. Emission quenching of PC **1** (10 µM) in degassed acetonitrile upon increasing concentrations of benzylamine **1a** (0-10 equiv.) and Stern-Volmer quenching plot. $I_0/I = 0.0028\ \mu M^{-1}\cdot[Q] + 1.0082$, $R^2 = 0.992$.

Photocatalytic oxidation of thioanisole and other thioether derivatives

Table S2. Comparison of representative photocatalysts with PC **1** for the oxidation of thioethers.

PC	Substrate	PC loading	Irradiation system	Reaction conditions	Conv. (%)	Ref.
Thiazole linked TTF-COF	Thioanisole	4 mg / 0.3 mmol substrate	Blue LED (460 nm)	O_2, RT, 30 min, EtOH	93 (99)	a
[(C^N)2Ir(N^N)]+ within a MOF	Thioanisole	4.0	100 W blue LED	O_2, RT, 6 h, H_2O	100	b
$[(C^N)_2Ir(N^N)]^+$<br>C^N = ppy linked to porous organic polymers<br>N^N = 4,4′-di(*t*Bu)-2,2′-bpy	Thioanisole	1.0	34 W blue LED (x 2)	O_2, RT, 10 h, MeOH	>99 (94)	c
[(C^N^N^N^N^C)Ir]+	Thioanisole	1.0	80 W blue LED	O_2, RT, 14 h, H_2O	99	d
$[(C^N^N^N^N^C)Ir]^+$<br>C^N^N^N^N^C = macrocycle	Thioanisole	1.0	80 W LED	O_2, RT, 8 h, H_2O	98	e
$[Ru(N^N)_3]_4$–⊂N⁺-COF<br>N^N = 2,2'-bipyridine-4,4'-dicarboxylate	Thioanisole	1 mg[a]	450 nm	O_2, RT, 3 h, MeCN/H_2O (1:1)	>99	f
$[(ppy)_2Ir(N^N)]$ within a COF<br>N^N = 1,10-phen motif	Thioanisole	0.5	12 W blue LED	O_2, RT, 7.5 h, MeOH	99	g
$[Ru(N^N^N)_2]$2+<br>N^N^N = terpy-R motif within porous poycarbazole	Thioanisole	0.25	23 W white LED	O_2, RT, 8 h, MeOH	97	h
$[(ppy)_2Ir(N^N)]$<br>N^N = 2,2′-dipyridylamine derivative	Thioanisole	0.1	24 W blue LED (460 nm)	Air, RT, 12 h, DMSO-*d*$_6$/D_2O (3:2)	>99	i
$[(ppy\text{-}F_2)_2Ir(N^O)]$<br>N^O = 2-(benzo-thiazolyl)phenolate	Thioanisole	0.1	24 W blue LED (460 nm)	O_2, RT, 18 h, DMSO-*d*$_6$/D_2O (3:2)	94	j
$[(C^N)_2Ir(N^N)]^+$<br>C^N = 2-phenylquinoline<br>N^N = 4,4′-dimethoxy-2,2′-bpy	Thioanisole	0.1	80 W blue LED	O_2, RT, 16 h, DMSO/H2O (3:2)	100	k
$[(ppy)_2Ir(N^N)]$<br>N^N = dipyrrinato derivative	Thioanisole	0.05	24 W white light (96 mW cm^{-2})	O_2, RT, 2 h, MeOH	98	l
**1**	Thioanisole	0.05	2.3 W blue LED (447 nm, 59.2 mW cm^{-2})	Air, RT, 24 h, MeCN	99 (100)	This work

Values in parentheses correspond to the selectivity toward sulfoxide.

a. Y. Wang, F. Huang, W. Sheng, X. Miao, X. Li, X. Gu, X. Lang, *Appl. Catal. B: Environ.* **2023**, *338*, 123070.

b. L. Wei, B. Ye, *ACS Appl. Mater. Interfaces* **2019**, *11*, 41448–41457.
c. Z. Xu, D. Zhang, H. Wang, X. Sun, Z. Li, *Green Chem.* **2020**, *22*, 136–143.
d. J. Fan, S. Yao, B. Ye, *Inorganics* **2024**, *12*, 73.
e. X. Huang, H. Zhou, B. Ye, *ACS Omega* **2024**, *9* (23), 24654–24664.
f. Y. Wang, Y. Wang, J. Li, Y. Yu, S. Huang, G. Yang, *Dalton Trans.* **2023**, *52*, 14100–14109.
g. Y. Lin, H. Cai, Q. Li, Y. Shao, L. Ran, H. Teng, *Materials Today Energy* **2026**, *56*, 102189.
h. D. A. Anito, T. Wang, H. Liang, X. Ding, B. Han, *Polym. Chem.* **2021**, *12*, 4557–4564.
i. M. Vaquero, A. Ruiz-Riaguas, M. Martínez-Alonso, F. A. Jalón, B. R. Manzano, A. M. Rodríguez, G. García-Herbosa, A. Carbayo, B. García, G. Espino, *Chem. Eur. J.* **2018**, *24*, 10662 – 10671.
j. M. Martínez-Alonso, N. Busto, L. D. Aguirre, L. Berlanga, M. C. Carrión, J. V. Cuevas, A. M. Rodríguez, A. Carbayo, B. R. Manzano, E. Ortí, F. A. Jalón, B. García, G. Espino, *Chem. Eur. J.* **2018**, *24*, 17523 – 17537.
k. L. Li, B. Ye, *Inorg. Chem.* **2019**, *58*, 7775–7784.
l. S. Kumar, L. Devi, I. Gupta, *New J. Chem.* **2025**, *49*, 11080–11088.

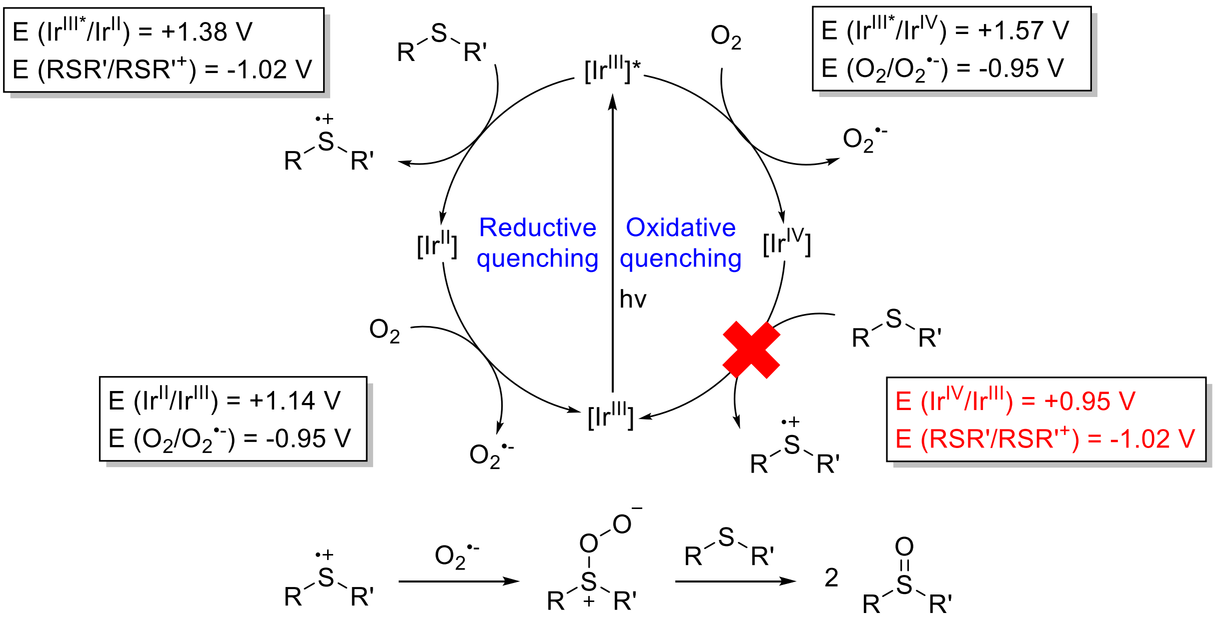

Figure S15. Schematic representation of the electron transfer pathways for the photocatalytic oxidation of thioethers with complex **1**. The oxidative quenching pathway is discarded, as Ir(IV) cannot be reduced back to Ir(III). Redox potentials of the thioether correspond to those reported for thioanisole. [4]

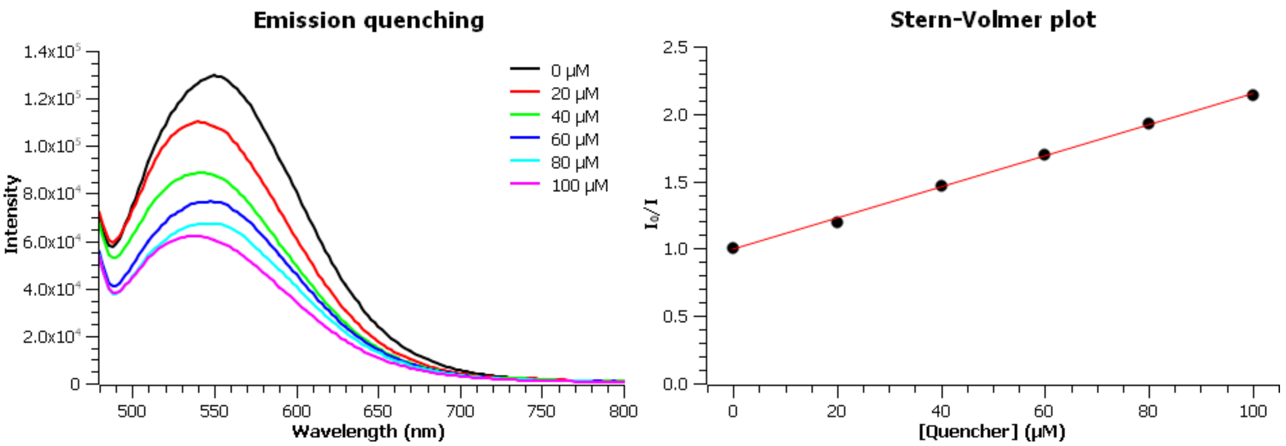

Figure S16. Emission quenching of PC **1** (10 μM) in degassed acetonitrile upon increasing concentrations of thioanisole **3a** (0-10 equiv.) and Stern-Volmer quenching plot. $I_0/I = 0.0116\ \mu M^{-1} \cdot [Q] + 0.9985$, $R^2 = 0.9990$.

Photocatalytic dehydrogenation of 1,2,3,4-tetrahydroquinoline and other N-heterocycles

Table S3. Comparison of representative photocatalysts with PC **1** for the dehydrogenation of N-heterocycles.

PC	Subst.	PC loading	Irradiation system	Reaction conditions	Conv. (%)	Ref.
h-BCN	THQ	10 mg / 0.30 mmol substrate	Blue LED	Air, RT, 12 h, H_2O	79	a
[Cp*Ir(C^N)(MeCN)]$^+$ C^N = mesoionic triazolylidene	THQ	2.0	50 W blue LEDs (x 2, 455 nm, 96 mW cm^{-2})	N_2, RT, 18 h, MeOH	97	b
Rose Bengal	THQ	1.0	32 W visible light	Air, RT, 24 h, DMA	95	c
$[Ru(bpy)_3]^{+2}$	THQ	1.0	36 W blue LED	Ar, RT, 12 h, $[Co(dmgH)_2$(4-$MeCO_2Py)Br]$ (2.5 mol %), iPrOH	76	d
$[Ru(bpy)_3]^{+2}$	THQ	1.0	35 W blue LED	Ar, RT, 8 h, $Co(dmgH)_2PyC$ (2 mol %), EtOH	96	e
Rh/TiO_2	THQ	1.0 wt %	Blue LED (453 nm)	Air, RT, 24 h, iPrOH	99	f
$[(ppy\text{-}F_2)_2Ir(N^\wedge N)]^+$ N^N = 2-pyridylbenzimidazole derivative	THQ	0.7	24 W blue LED (460 nm)	O_2, RT, 24 h, MeCN	>99	g
**1**	THQ	0.25	2.3 W blue LED (447 nm, 59.2 mW cm^{-2})	O_2, RT, 24 h, MeCN	99	This work
$[(ppy)_2Ir(N^\wedge N)]^+$ N^N = β-carboline	Indoline	0.1	24 W blue LED (460 nm)	O_2, RT, 24 h, THF	92	h
Ni/TiO_2	THQ	0.1	Blue LED (453 nm, 4 mW cm^{-2})	O_2, RT, 24 h, 4-aminoTEMPO (20 mol %), iPrOH	91	i

h-BCN = hexagonal boron carbon nitride nanosheets. DMA = *N*,*N*-dimethylacetamide.

a. M. Zheng, J. Shi, T. Yuan, X. Wang, *Angew. Chem.* **2018**, *130*, 5585 –5589.
b. C. Mejuto, L. Ibáñez-Ibáñez, G. Guisado-Barrios, J. A. Mata, *ACS Catal.* **2022**, *12*, 6238–6245.
c. M. K. Sahoo, G. Jaiswal, J. Rana, E. Balaraman, *Chem. Eur. J.* **2017**, *23*, 14167 – 14172.
d. M. K. Sahoo, E. Balaraman, *Green Chem.* **2019**, *21*, 2119–2128.

e. K. He, F. Tan, C. Zhou, G. Zhou, X. Yang, Y. Li, *Angew. Chem. Int. Ed.* **2017**, *56*, 3080 –3084.
f. N. O. Balayeva, Z. Mamiyev, R. Dillert, N. Zheng, D. W. Bahnemann, *ACS Catal.* **2020**, *10*, 5542–5553.
g. I. Echevarría, M. Vaquero, B. R. Manzano, F. A. Jalón, R. Quesada, G. Espino, *Inorg. Chem.* **2022**, *61*, 6193–6208.
h. J. Sanz-Villafruela, C. Martínez-Alonso, I. Echevarría, M. Vaquero, A. Carbayo, J. Fidalgo, A. M. Rodríguez, J. V. Cuevas-Vicario, J. C. Lima, A. J. Moro, B. R. Manzano, F. A. Jalón, G. Espino, *Inorg. Chem. Front.* **2021**, *8*, 1253–1270.
i. N. O. Balayeva, N. Zheng, R. Dillert, D. W. Bahnemann, *ACS Catal.* **2019**, *9*, 10694–10704.

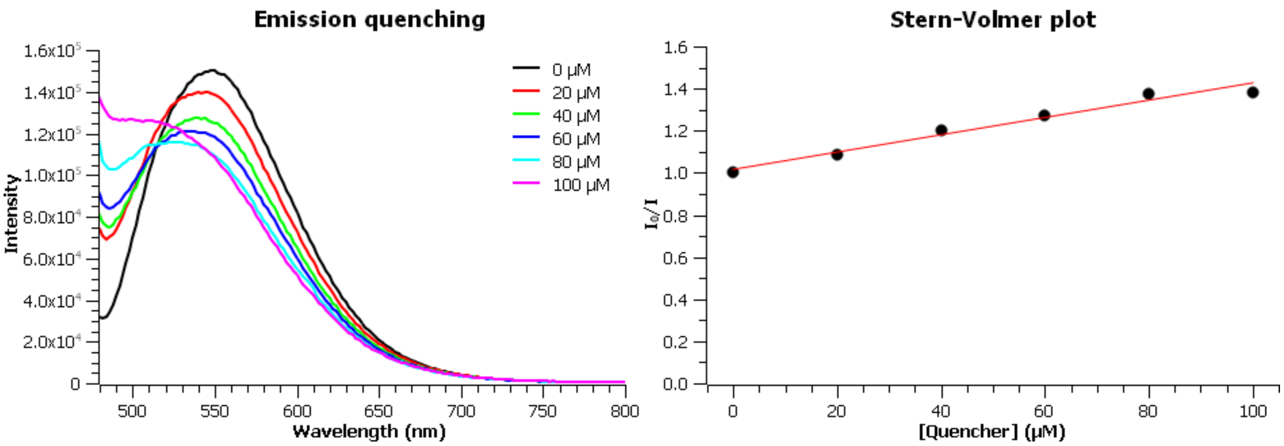

Figure S17. Emission quenching of PC **1** (10 µM) in degassed acetonitrile upon increasing concentrations of 1,2,3,4-tetrahydroquinoline **6a** (0-10 equiv.) and Stern-Volmer quenching plot. $I_0/I = 0.0041\ \mu M^{-1}\cdot[Q] + 1.0139$, $R^2 = 0.97$.

Characterization of the photocatalytic products.

N-benzyl-1-phenylmethanimine (**2a**)

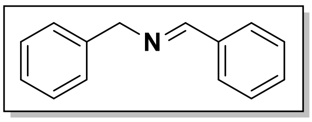

The product was purified by column chromatography in neutral alumina with EtOAc:hexane (1:4). The product was obtained as a white solid. ^1H NMR (400 MHz, acetonitrile-d_3): δ 8.47 (m, 1H), 7.79 – 7.76 (m, 2H), 7.47 – 7.42 (m, 3H), 7.35 (d, *J* = 4.4 Hz, 4H), 7.27 (ddd, *J* = 8.1, 4.9, 3.9 Hz, 1H), 4.77 (d, *J* = 1.4 Hz, 2H). ^{13}C NMR (101 MHz, acetonitrile-d_3): δ 126.78, 131.70, 129.69, 129.44, 129.02, 128.99, 127.87, 65.56.

1-(pyridin-2-yl)-*N*-(pyridin-2-ylmethyl)methanimine (**2b**)

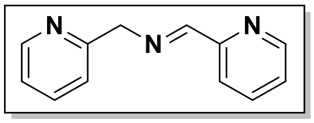

The product could not be isolated. The crude of the reaction was analysed. ^1H NMR (400 MHz, acetonitrile-d_3): δ 8.62 (dd, *J* = 3.8, 2.3 Hz, 1H), 8.54 – 8.47 (m, 2H), 8.00 (dt, *J* = 7.9, 1.1 Hz, 1H), 7.84 – 7.76 (td, *J* = 7.8, 1.6 Hz, 1H), 7.72 (td, *J* = 7.7, 1.8 Hz, 1H), 7.44 – 7.34 (m, 2H), 7.22 (dd, *J* = 7.6, 4.9 Hz, 1H), 4.91 (s, 2H). ^{13}C NMR (101 MHz, acetonitrile-d_3): δ 164.95, 159.90, 150.52, 150.25, 137.75, 126.08, 123.36, 123.22, 121.63, 67.09.

1-(pyridin-4-yl)-N-(pyridin-4-ylmethyl)methanimine (**2c**)

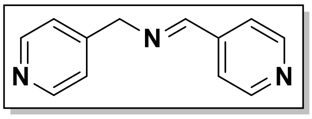

The product could not be isolated. The crude of the reaction was analysed. ^{1}H NMR (400 MHz, acetonitrile-d_3): δ 8.65 (m, 2H), 8.50 (m, 2H), 8.47 (t, *J* = 1.5 Hz, 1H), 7.66 (m, 2H), 7.32 (m, 2H), 4.82 (s, 2H). ^{13}C NMR (101 MHz, acetonitrile-d_3): δ 162.83, 151.46, 151.40, 150.76, 123.85, 122.86, 122.27, 63.83.

N-(4-chlorobenzyl)-1-(4-chlorophenyl)methanimine (**2d**)

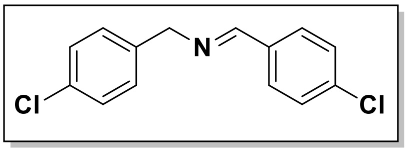

The product was crystallized by slow evaporation of an acetonitrile solution as a white solid. ^{1}H NMR (500 MHz, acetonitrile-d_3): δ 8.44 – 8.42 (t, *J* = 1.6 Hz, 1H), 7.78 – 7.73 (m, 2H), 7.48 – 7.43 (m, 2H), 7.38 – 7.30 (m, 4H), 4.75 (d, *J* = 1.4 Hz, 2H). ^{13}C NMR (126 MHz, acetonitrile-d_3): δ 161.89, 139.59, 136.99, 136.03, 133.01, 130.52, 130.42, 129.75, 129.30, 64.40.

4-(((4-cyanobenzyl)imino)methyl)benzonitrile (**2e**)

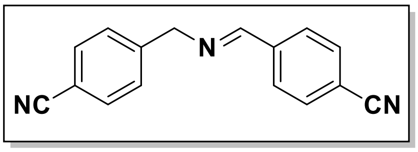

The product could not be isolated. The crude of the reaction was analysed. ^{1}H NMR (500 MHz, acetonitrile-d_3): δ 8.51 (t, *J* = 1.6 Hz, 1H), 7.91 (m, 2H), 7.78 (m, 2H), 7.69 (m, 2H), 7.51 (d, *J* = 8.1 Hz, 2H), 4.86 (d, *J* = 1.4 Hz, 2H).

4-(((4-(dimethylamino)benzyl)imino)methyl)-N,N-dimethylaniline (**2g**)

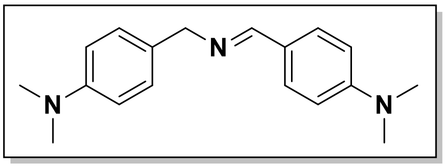

The product could not be isolated. The crude of the reaction was analysed. ^{1}H NMR (500 MHz, acetonitrile-d_3): δ 8.22 (s, 1H), 7.55 (m, 2H), 7.05 (m, 2H), 6.71 (m, 4H), 4.54 (s, 2H), 2.95 (s, 12H).

N-(4-methylbenzyl)-1-(p-tolyl)methanimine (**2h**)

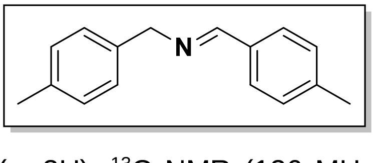

The product was crystallized by slow evaporation of an acetonitrile solution as a white solid. ^{1}H NMR (500 MHz, acetonitrile-d_3): δ 8.39 (m, 1H), 7.67 – 7.63 (m, 2H), 7.25 (d, *J* = 7.8 Hz, 2H), 7.22 (d, *J* = 8.1 Hz, 2H), 7.16 (d, *J* = 7.9 Hz, 2H), 4.70 (s, 2H), 2.37 (s, 3H), 2.31 (s, 3H). ^{13}C NMR (126 MHz, acetonitrile-d_3): δ 162.31, 142.05, 137.95, 137.47, 134.98, 130.29, 130.00, 128.97, 65.33, 21.49, 21.11.

4-(((4-aminobenzyl)imino)methyl)aniline (**2i**)

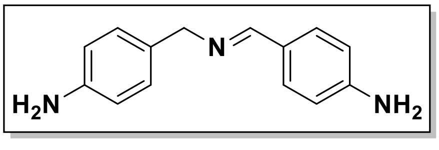

The product could not be isolated. The crude of the reaction was analysed. ^{1}H NMR (500 MHz, acetonitrile-d_3): δ 8.20 (s, 1H), 7.50 – 7.43 (m, 2H), 7.04 – 6.99 (m, 2H), 6.65 – 6.62 (m, 2H), 6.61 – 6.57 (m, 2H), 4.52 (s, 2H), 4.48 (s, 2H), 4.09 (s, 2H).

N-(4-methoxybenzyl)-1-(4-methoxyphenyl)methanimine (**2j**)

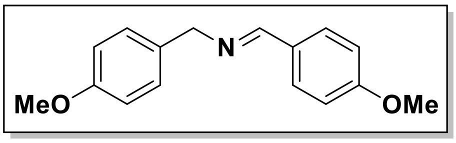

The product could not be isolated. The crude of the reaction was analysed. ^{1}H NMR (500 MHz, acetonitrile-d_3): δ 8.33 (d, *J* = 1.6 Hz, 1H), 7.70 – 7.64 (m, 2H), 7.24 – 7.20 (m, 2H), 6.97 – 6.92 (m, 2H), 6.89 – 6.85 (m, 2H), 4.63 (d, *J* = 1.3 Hz, 2H), 3.80 (s, 3H), 3.74 (s, 3H).

N-(2,4-dimethoxybenzyl)-1-(2,4-dimethoxyphenyl)methanimine (**2k**)

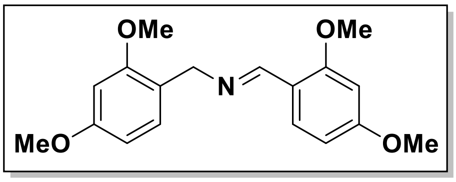

The product could not be isolated. The crude of the reaction was analysed. ^{1}H NMR (500 MHz, acetonitrile-d_3): δ 8.62 (s, 1H), 7.79 (d, *J* = 8.6 Hz, 1H), 7.11 (dd, *J* = 8.3, 3.0 Hz, 1H), 6.54 – 6.49 (m, 1H), 6.49 – 6.45 (m, 1H), 4.59 (s, 2H), 3.83 (s, 3H), 3.79 (s, 3H), 3.78 (s, 3H), 3.74 (s, 3H).

1-phenyl-*N*-(1-phenylethyl)ethan-1-imine (**2l**)

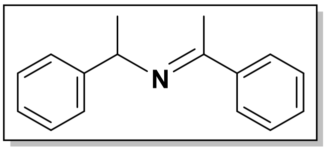

The product was purified by column chromatography in neutral alumina with EtOAc:hexane (1:4). The product was obtained as a white solid. ^{1}H NMR (400 MHz, acetonitrile-d_3): δ 7.83 – 7.80 (m, 2H), 7.55 – 7.50 (m, 1H), 7.48 – 7.43 (m, 2H), 7.41 – 7.39 (m, 2H), 7.36 – 7.32 (m, 2H), 7.26 – 7.22 (m, 1H), 5.19 (q, J = 7.1 Hz, 1H), 1.93 (s, 3H), 1.53 (d, J = 7.1 Hz, 3H). ^{13}C NMR (101 MHz, acetonitrile-d_3): δ 167.24, 145.86, 135.80, 132.23, 129.41, 129.35, 128.12, 127.82, 127.01, 50.31, 22.65.

1-(4-methoxyphenyl)-N-(1-(4-methoxyphenyl)ethyl)ethan-1-imine (**2m**)

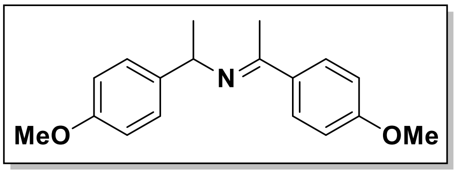

The product was purified by column chromatography in neutral alumina with EtOAc:hexane (1:4). The product was obtained as a white solid. ^{1}H NMR (500 MHz, acetonitrile-d$_3$): δ 7.94 (m, 4H), 7.00 (m, 4H), 3.86 (s, 6H), 2.51 (s, 6H).

1-(naphthalen-2-yl)-N-(1-(naphthalen-2-yl)ethyl)ethan-1-imine (**2n**)

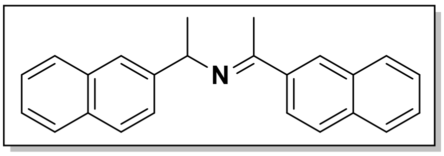

The product was purified by column chromatography in neutral alumina with EtOAc:hexane (1:4). The product was obtained as a white solid. ^{1}H NMR (500 MHz, acetonitrile-d_3): δ 8.34 (d, *J* = 8.5 Hz, 1H), 8.23 – 8.17 (m, 1H), 7.98 – 7.94 (m, 2H), 7.94 – 7.90 (m, 1H), 7.85 (d, *J* = 8.2 Hz, 1H), 7.68 (dt, *J* = 7.5, 1.1 Hz, 1H), 7.67 – 7.63 (m, 1H), 7.61 (dd, *J* = 7.0, 1.2 Hz, 1H), 7.59 – 7.55 (m, 1H), 7.55 – 7.48 (m, 4H), 6.12 (q, *J* = 7.1 Hz, 1H), 2.16 (s, 3H), 1.72 (d, *J* = 7.0 Hz, 3H). ^{13}C NMR (126 MHz, acetonitrile-d_3): δ 169.14, 140.76, 135.67, 134.81, 134.47, 131.00, 130.91, 129.73, 129.13, 128.61, 127.69, 127.28, 127.24, 126.71, 126.49, 126.24, 125.98, 125.82, 124.12, 123.48, 46.15, 21.55.

N-benzhydryl-1,1-diphenylmethanimine (**2o**)

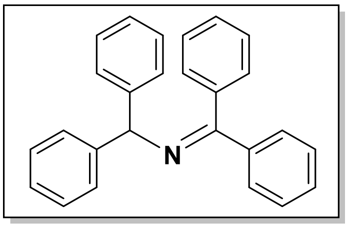

The product was crystallized by slow evaporation of an acetonitrile solution as a white solid. ^{1}H NMR (500 MHz, acetonitrile-d_3): δ 7.52 – 7.48 (m, 4H), 7.33 – 7.22 (m, 8H), 7.21 – 7.17 (m, 4H), 7.16 – 7.11 (m, 8H), 7.11 – 7.07 (m, 8H), 7.03 – 6.98 (m, 4H), 6.88 – 6.84 (m, 4H), 5.35 (s, 2H). ^{13}C NMR (126 MHz, acetonitrile-d_3): δ 168.21, 145.98, 140.70, 137.44, 131.25, 129.65, 129.51, 129.38, 129.32, 129.14, 128.43, 128.23, 127.77, 70.71.

N-propylpropan-1-imine (**2q**)

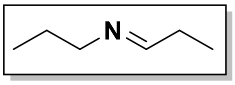

The product could not be isolated. The crude of the reaction was analysed. ^{1}H NMR (500 MHz, acetonitrile-d_3): δ 7.57 (t, J = 1.5 Hz, 1H), 3.57 (td, J = 6.9, 1.5 Hz, 2H), 1.80 (dt, J = 4.9, 2.5 Hz, 2H), 1.67 (m, 2H), 0.96 (m, 3H), 0.92 (m, 3H).

N-butylbutan-1-imine (**2r**)

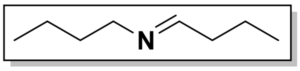

The product could not be isolated. The crude of the reaction was analysed. ^{1}H NMR (400 MHz, acetonitrile-d_3): δ 7.59 (t, J = 1.5 Hz, 1H), 2.78 (q, J = 7.4 Hz, 2H), 1.03 (m, 4H), 0.92 (m, 10H). ^{13}C NMR (101 MHz, acetonitrile-d_3): δ 160.83, 61.54, 56.41, 30.89, 21.04, 14.05.

2-ethyl-*N*-(2-ethylhexyl)hexan-1-imine (**2s**)

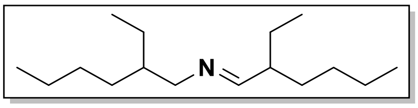

The product could not be isolated. The crude of the reaction was analysed. ^{1}H NMR (400 MHz, CD_3OD): δ 7.67 (s, 1H), 1.79 – 1.54 (m, 4H), 1.51 – 1.26 (m, 16H), 0.95 – 0.84 (m, 12H). ^{13}C NMR (101 MHz, CD_3OD): δ 163.89, 91.17, 82.74, 42.81, 41.67, 40.58, 36.52, 31.97, 29.97, 27.11, 26.63, 25.14, 24.16, 24.07, 23.38, 20.16, 17.52, 14.41, 14.25, 14.22, 11.17, 8.08, 8.05.

N-(prop-2-yn-1-yl)prop-2-yn-1-imine (**2t**)

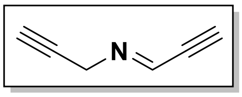

The product could not be isolated. The crude of the reaction was analysed. ^{1}H NMR (400 MHz, acetonitrile-d_3): δ 8.04 (m, 1H), 3.94 – 3.91 (m, 1H), 3.53 (s, 2H), 2.42 (t, J = 2.5 Hz, 1H). ^{13}C NMR (101 MHz, acetonitrile-d_3): δ 161.87, 71.79, 71.09, 30.90.

N-cyclohexylcyclohexanimine (**2u**)

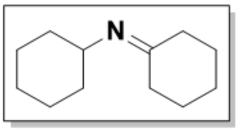

The product could not be isolated. The crude of the reaction was analysed. ^{1}H NMR (400 MHz, CD_3OD): δ 3.72 – 3.55 (m, 1H), 2.32 – 2.11 (m, 2H), 1.86 – 1.57 (m, 10H), 1.40 – 1.14 (m, 8H). ^{13}C NMR (101 MHz, CD_3OD): δ 162.74, 51.49, 33.82, 33.76, 33.69, 33.29, 31.96, 31.04, 26.57, 26.12, 25.88, 25.35.

(Methylsulfinyl)benzene (**4a**)

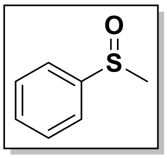

The product was purified by column chromatography in silica with EtOAc:hexane (1:3). The product was obtained as a white solid. ^{1}H NMR (400 MHz, acetonitrile-d_3): δ 7.67 – 7.64 (m, 2H), 7.59 – 7.51 (m, 3H), 2.66 (s, 3H). ^{13}C NMR (101 MHz, acetonitrile-d_3): δ 147.58, 131.71, 130.20, 124.33, 44.23.

1-methyl-4-(methylsulfinyl)benzene (**4b**)

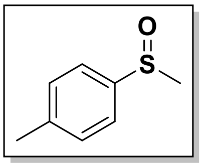

The product was purified by column chromatography in silica with EtOAc:hexane (1:4). The product was obtained as a white solid. ^{1}H NMR (500 MHz, acetonitrile-d_3): δ 7.55 – 7.52 (m, 2H), 7.39 – 7.36 (m, 2H), 2.64 (s, 3H), 2.40 (s, 3H). ^{13}C NMR (101 MHz, acetonitrile-d_3): δ 144.52, 142.38, 130.88, 124.50, 44.34, 21.36.

1-methoxy-4-(methylsulfinyl)benzene (**4c**)

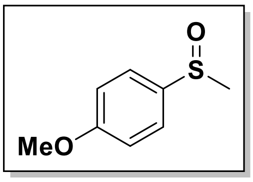

The product was purified by column chromatography in silica with EtOAc:hexane (1:4). The product was obtained as a yellow liquid. ^{1}H NMR (500 MHz, acetonitrile-d_3): δ 7.59 (m, 2H), 7.09 (m, 2H), 3.84 (s, 3H), 2.63 (s, 3H). ^{13}C NMR (126 MHz, acetonitrile-d_3): δ 162.85, 138.55, 126.44, 115.73, 56.30, 44.32.

4-(methylsulfinyl)aniline (**4d**)

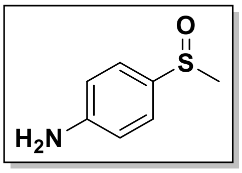

The product was purified by column chromatography in silica with EtOAc:hexane (1:4). The product was obtained as a yellow solid. ^{1}H NMR (500 MHz, acetonitrile-d_3): δ 7.39 – 7.36 (m, 2H), 6.76 – 6.73 (m, 2H), 2.59 (s, 3H). ^{13}C NMR (126 MHz, acetonitrile-d_3): δ 151.96, 133.61, 130.07, 126.41, 115.23, 114.25, 44.06.

4-(methylsulfinyl)benzaldehyde (**4e**)

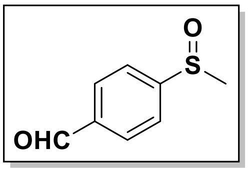

The product could not be isolated. The crude of the reaction was analysed. ^{1}H NMR (400 MHz, acetonitrile-d_3): δ 10.04 (s, 1H), 8.04 – 8.02 (m, 2H), 7.82 – 7.80 (m, 2H), 2.69 (s, 3H).

1-(methylsulfinyl)-4-nitrobenzene (**4f**)

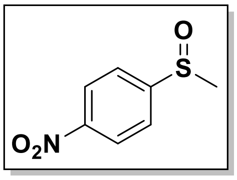

The product was purified by column chromatography in silica with EtOAc:hexane (1:3). The product was obtained as a white solid. ^{1}H NMR (500 MHz, acetonitrile-d_3): δ 8.37 – 8.35 (m, 2H), 7.87 – 7.85 (m, 2H), 2.75 (s, 3H). ^{13}C NMR (126 MHz, acetonitrile-d_3): δ 155.17, 150.49, 125.86, 125.27, 44.12.

1-chloro-4-(methylsulfinyl)benzene (**4g**)

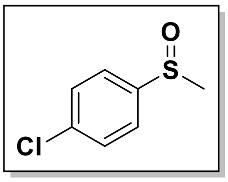

The product was purified by column chromatography in silica with EtOAc:hexane (1:3). The product was obtained as a yellow liquid. ^{1}H NMR (500 MHz, acetonitrile-d_3) δ 7.63(m, 2H), 7.57 (m, 2H), 2.67 (s, 3H). ^{13}C NMR (126 MHz, acetonitrile-d_3): δ 146.60, 137.21, 130.37, 126.31, 44.33.

Diphenyl sulfoxide (**4i**)

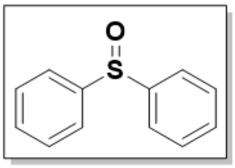

The product was purified by column chromatography in silica with EtOAc:hexane (1:1). The product was obtained as a white solid. ^{1}H NMR (400 MHz, acetonitrile-d_3): δ 7.69 – 7.67 (m, 4H), 7.53 – 7.47 (m, 6H). ^{13}C NMR (101 MHz, acetonitrile-d_3): δ 147.40, 132.09, 130.45, 125.15.

Dibenzo[*b*,*d*]thiophene 5-oxide (**4j**)

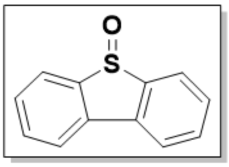

The product was purified by column chromatography in silica with EtOAc:hexane (1:3). The product was obtained as a white solid. ^{1}H NMR (400 MHz, acetonitrile-d_3) δ 8.01 – 7.97 (m, 4H), 7.68 (td, *J* = 7.6, 1.1 Hz, 2H), 7.57 (td, *J* = 7.6, 1.1 Hz, 2H). ^{13}C NMR (101 MHz, acetonitrile-d_3): δ 146.38, 137.78, 133.65, 130.67, 128.15, 123.36.

1-(propylsulfinyl)propane (**4l**)

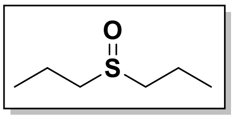

The product was purified by column chromatography in silica with EtOAc:hexane (1:4). The product was obtained as a colourless liquid. ^{1}H NMR (500 MHz, acetonitrile-d_3): δ 2.63 – 2.59 (m, 4H), 1.76 – 1.69 (m, 4H), 1.04 (t, *J* = 7.4 Hz, 6H). ^{13}C NMR (126 MHz, acetonitrile-d_3): δ 54.68, 17.01, 13.57.

Tetrahydrothiophene 1-oxide (**4n**)

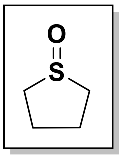

The product could not be isolated. The crude of the reaction was analysed. ^{1}H NMR (500 MHz, acetonitrile-d_3): δ 2.70 (m, 4H), 2.52 (td, *J* = 7.2, 1.4 Hz, 2H), 2.27 (m, 2H).

Quinoline (**8a**)

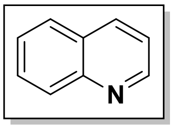

The product was purified by column chromatography in silica with EtOAc:hexane (2:3). The product was obtained as a colourless liquid. ^{1}H NMR (400 MHz, acetone-d_6): δ 8.90 (dd, *J* = 4.2, 1.8 Hz, 1H), 8.32 (ddd, *J* = 8.3, 1.8, 0.8 Hz, 1H), 8.05 (dq, *J* = 8.5, 1.0 Hz, 1H), 7.95 (dd, *J* = 8.1, 1.5 Hz, 1H), 7.76 (ddd, *J* = 8.4, 6.9, 1.5 Hz, 1H), 7.60 (ddd, *J* = 8.1, 6.8, 1.2 Hz, 1H), 7.50 (dd, *J* = 8.3, 4.2 Hz, 1H). ^{13}C NMR (101 MHz, acetone-d_6): δ 151.36, 149.39, 136.61, 130.29, 130.10, 129.25, 128.25, 127.30, 122.16.

6-bromoquinoline (**8b**)

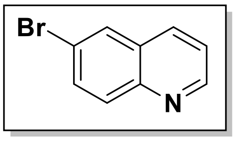

The product was purified by column chromatography in neutral alumina with EtOAc:hexane (1:2). The product was obtained as a pale brown liquid. ^{1}H NMR (500 MHz, acetone-d_6): δ 8.94 (dd, J = 4.2, 1.7 Hz, 1H), 8.32 (dd, J = 8.7, 1.7 Hz, 1H), 8.20 (d, J = 2.2 Hz, 1H), 7.99 (d, J = 8.9 Hz, 1H), 7.86 (dd, J = 9.0, 2.3 Hz, 1H), 7.56 (dd, J = 8.4, 4.2 Hz, 1H). ^{13}C NMR (126 MHz, acetone-d_6): δ 151.10, 147.00, 135.02, 132.49, 131.48, 130.05, 129.56, 122.24, 119.65.

6-nitroquinoline (**8c**)

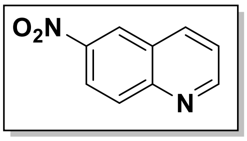

The product was purified by column chromatography in silica with EtOAc:hexane (1:2). The product was obtained as a yellow solid. ^{1}H NMR (500 MHz, acetone-d_6): δ 9.14 (dd, J = 4.2, 1.8 Hz, 1H), 9.00 (d, J = 2.6 Hz, 1H), 8.69 (ddd, J = 8.4, 2.0, 0.8 Hz, 1H), 8.50 (dd, J = 9.2, 2.6 Hz, 1H), 8.26 (d, J = 9.2 Hz, 1H), 7.74 (dd, J = 8.3, 4.2 Hz, 1H). ^{13}C NMR (126 MHz, acetone-d_6): δ 155.01, 151.12, 146.47, 138.93, 132.15, 128.15, 125.80, 124.05, 123.45.

6-hydroxyquinoline (**8d**)

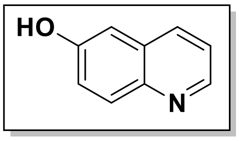

The product was purified by column chromatography in silica with EtOAc:hexane (1:2). The product was obtained as a pale brown solid. ^{1}H NMR (500 MHz, acetone-d_6): δ 9.01 (s, 1H), 8.69 (dd, J = 4.2, 1.7 Hz, 1H), 8.12 (d, J = 8.3 Hz, 1H), 7.93 (d, J = 9.1 Hz, 1H), 7.39 (m, 2H), 7.22 (d, J = 2.7 Hz, 1H). ^{13}C NMR (126 MHz, acetone-d_6): δ 156.49, 148.05, 144.60, 135.11, 131.58, 130.62, 122.63, 122.22, 109.37.

6-methoxyquinoline (**8e**)

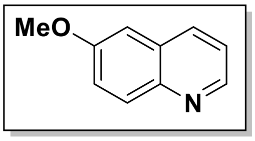

The product was purified by column chromatography in neutral alumina with EtOAc:hexane (1:2). The product was obtained as a pale brown solid. ^{1}H NMR (500 MHz, acetone-d_6): δ 8.73 (dd, J = 4.2, 1.7 Hz, 1H), 8.22 – 8.19 (m, 1H), 7.94 (dt, J = 9.2, 0.6 Hz, 1H), 7.42 (dd, J = 8.3, 4.2 Hz, 1H), 7.38 (dd, J = 9.2, 2.9 Hz, 1H), 7.31 (d, J = 2.9 Hz, 1H), 3.94 (s, 3H). ^{13}C NMR (126 MHz, acetone-d_6): δ 158.68, 148.75, 145.48, 135.41, 131.65, 130.35, 122.82, 122.36, 106.23, 55.93.

2-methylquinoline (**8f**)

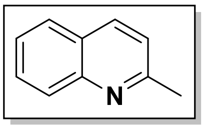

The product was purified by column chromatography in silica with EtOAc:hexane (2:3). The product was obtained as a yellow liquid. ^{1}H NMR (400 MHz, acetone-d_6): δ 8.18 (dd, J = 8.4, 0.8 Hz, 1H), 7.94 (dq, J = 8.5, 0.9 Hz, 1H), 7.88 (dd, J = 8.2, 1.4 Hz, 1H), 7.69 (ddd, J = 8.4, 6.9, 1.5 Hz, 1H), 7.51 (ddd, J = 8.1, 6.9, 1.2 Hz, 1H), 7.38 (d, J = 8.4 Hz, 1H), 2.67 (s, 3H). ^{13}C NMR (101 MHz, acetone-d_6): δ 159.70, 148.95, 136.72, 129.97, 129.59, 128.48, 127.46, 126.38, 122.76, 25.33.

3-methylquinoline (**8g**)

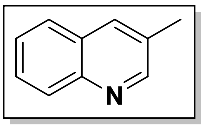

The product was purified by column chromatography in silica with EtOAc:hexane (1:2). The product was obtained as a colourless liquid. ^{1}H NMR (500 MHz, acetone-d_6): δ 8.78 (s, 1H), 8.08 (dt, J = 2.2, 1.2 Hz, 1H), 8.01 (d, J = 8.5 Hz, 1H), 7.86 (dd, J = 8.2, 1.4 Hz, 1H), 7.67 (ddd, J = 8.4, 6.8, 1.5 Hz, 1H), 7.56 (ddd, J = 8.1, 6.9, 1.2 Hz, 1H), 2.52 (s, 3H). ^{13}C NMR (126 MHz, acetone-d_6): δ 153.11, 129.87, 129.16, 128.19, 127.36, 18.63.

3,4-dihydroisoquinoline (**8h**)

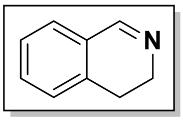

The product was purified by column chromatography in neutral alumina with EtOAc:hexane (1:4). The product was obtained as a white solid. ^{1}H NMR (400 MHz, acetonitrile-d_3): δ δ 7.92 (dd, J = 7.7, 1.5 Hz, 1H), 7.46 (td, J = 7.5, 1.5 Hz, 1H), 7.35 (d, J = 7.6 Hz, 1H), 7.28 (d, J = 6.8 Hz, 1H), 6.37 (s, 1H), 3.46 (td, J = 6.6, 2.9 Hz, 2H), 2.95 (t, J = 6.6 Hz, 2H). ^{13}C NMR (101 MHz, acetonitrile-d_3): δ 166.12, 140.65, 132.84, 130.35, 128.43, 128.24, 127.69, 40.61, 28.93.

1*H*-indole (**8i**)

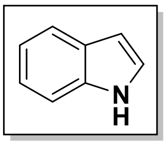

The product was purified by column chromatography in silica with EtOAc:hexane (1:4). The product was obtained as a yellow solid. ^{1}H NMR (400 MHz, acetone-d_6): δ 10.21 (s, 1H), 7.56 (dd, *J* = 7.8, 0.9 Hz, 2H), 7.42 (dd, *J* = 8.1, 0.9 Hz, 2H), 7.34 – 7.28 (m, 2H), 7.09 (ddd, *J* = 8.1, 7.0, 1.2 Hz, 2H), 7.00 (ddd, *J* = 8.0, 7.0, 1.1 Hz, 2H), 6.46 (ddd, *J* = 3.0, 1.9, 1.0 Hz, 2H). ^{13}C NMR (101 MHz, acetone-d_6): δ 137.23, 129.08, 125.52, 125.36, 122.01, 121.04, 119.87, 112.09, 102.31.

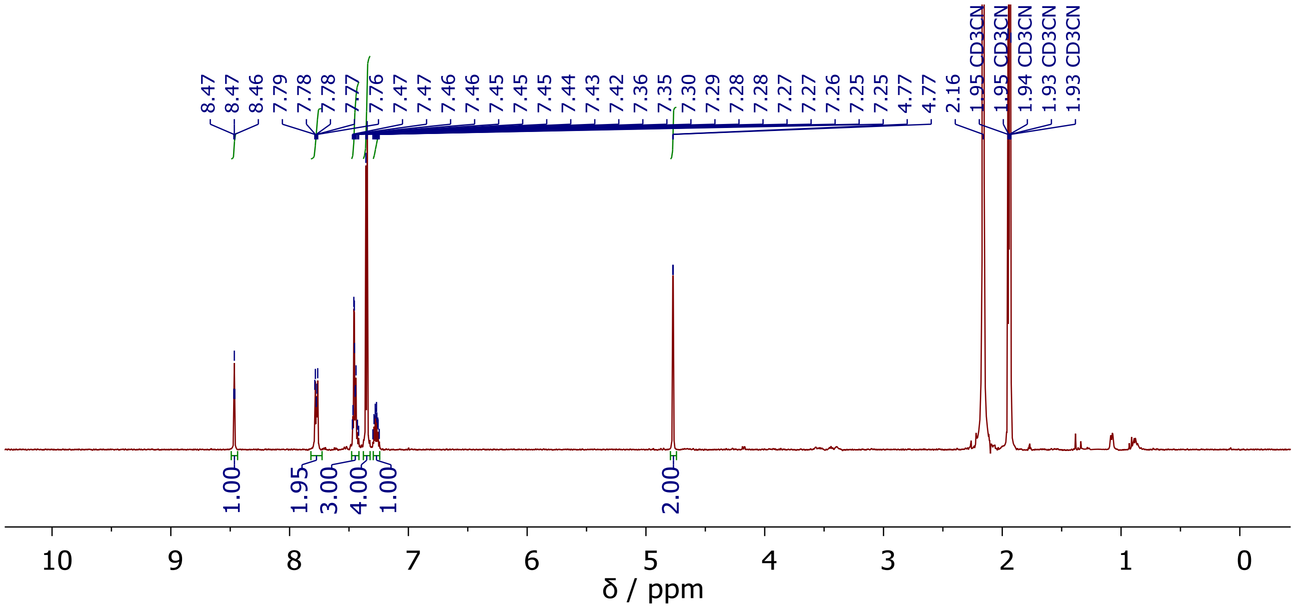

Figure S18. ^{1}H NMR (400 MHz, acetonitrile-d_3, 298 K) spectrum of *N*-benzyl-1-phenylmethanimine (**2a**).

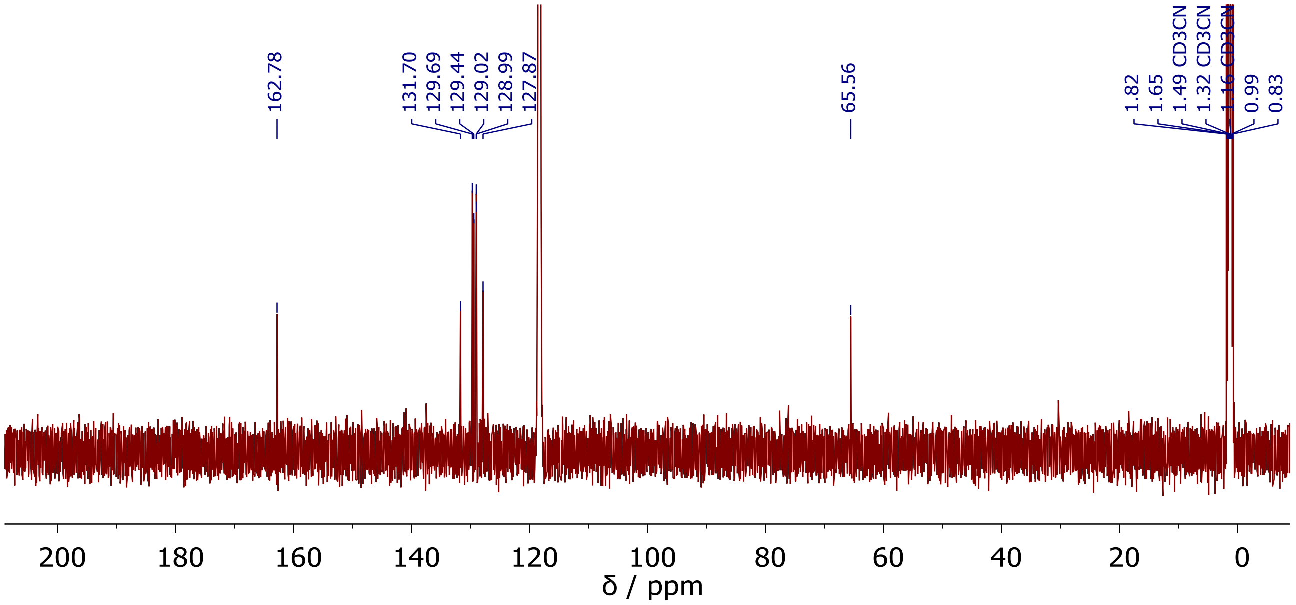

Figure S19. ^{13}C NMR (101 MHz, acetonitrile-d_3, 298 K) spectrum of *N*-benzyl-1-phenylmethanimine (**2a**).

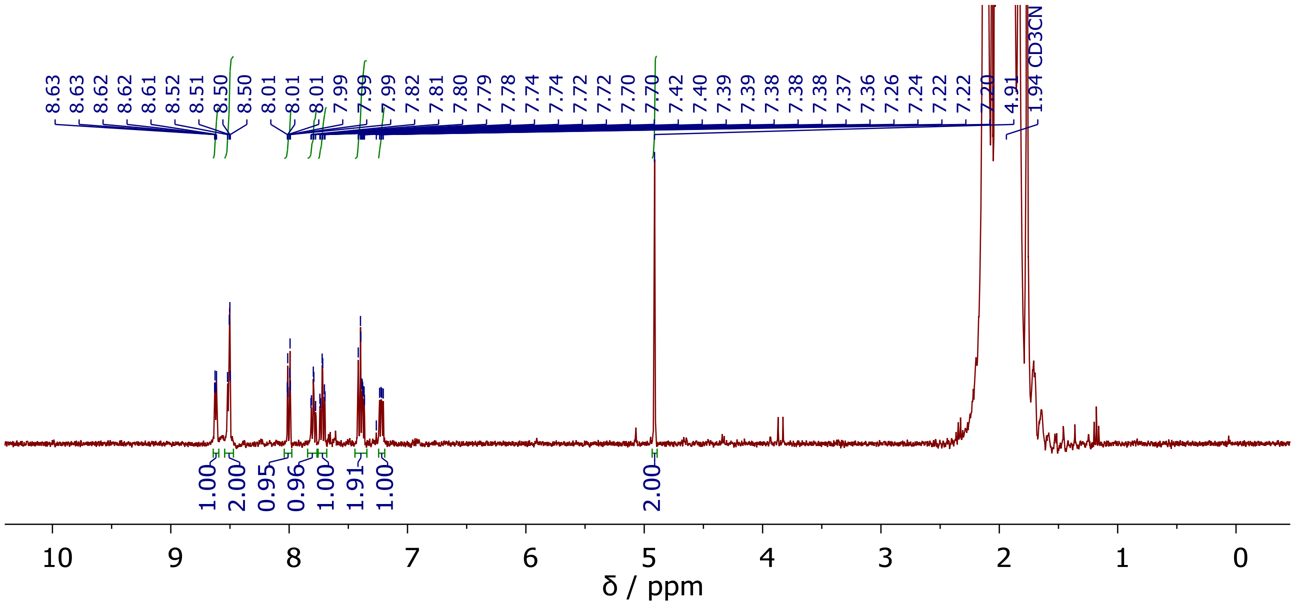

Figure S20. ^{1}H NMR (400 MHz, acetonitrile-d_3, 298 K) spectrum of 1-(pyridin-2-yl)-*N*-(pyridin-2-ylmethyl)methanimine (**2b**).

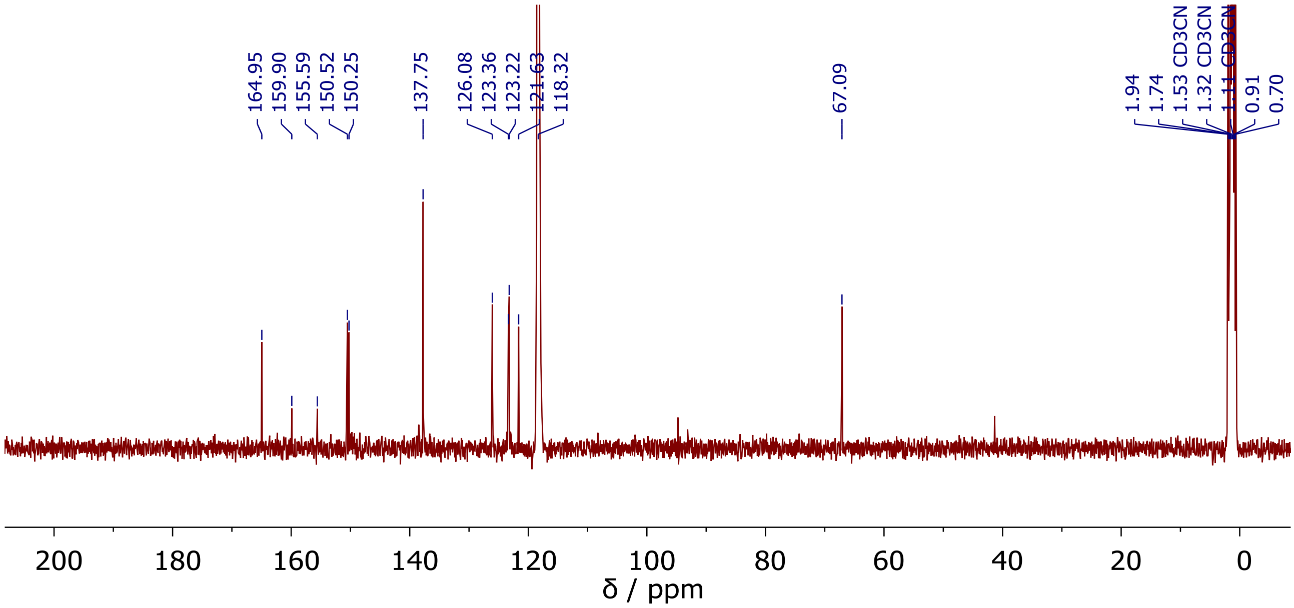

Figure S21. ^{13}C NMR (101 MHz, acetonitrile-d_3, 298 K) spectrum of 1-(pyridin-2-yl)-*N*-(pyridin-2-ylmethyl)methanimine (**2b**).

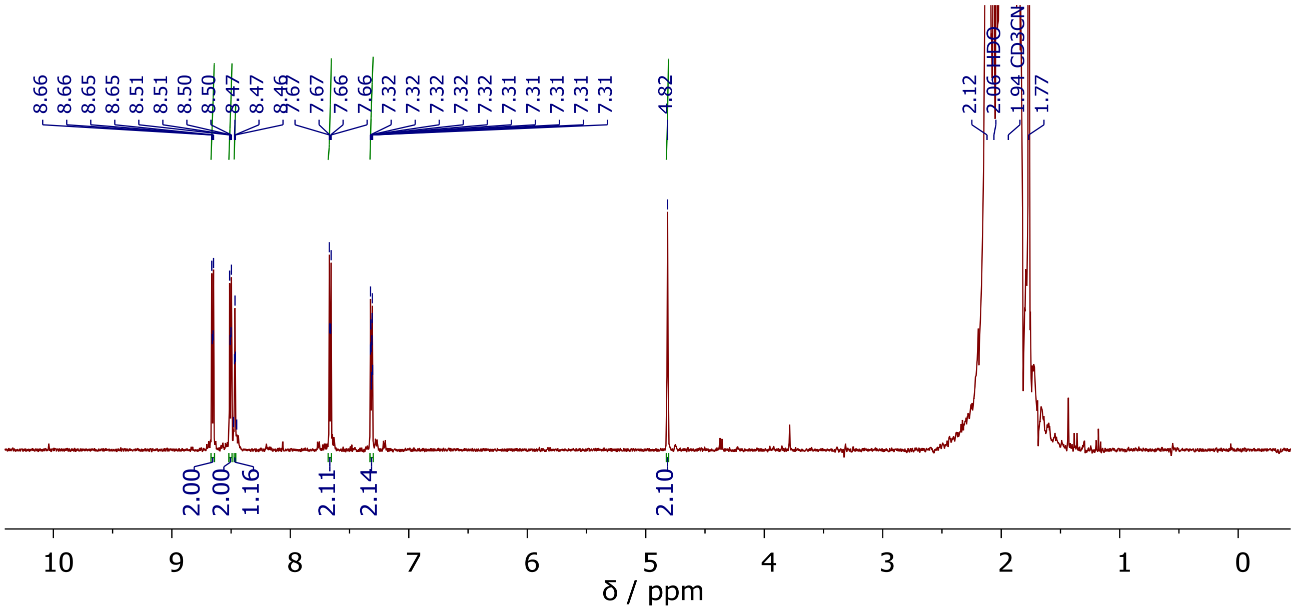

Figure S22. ^{1}H NMR (400 MHz, acetonitrile-d_3, 298 K) spectrum of 1-(pyridin-4-yl)-*N*-(pyridin-4-ylmethyl)methanimine (**2c**).

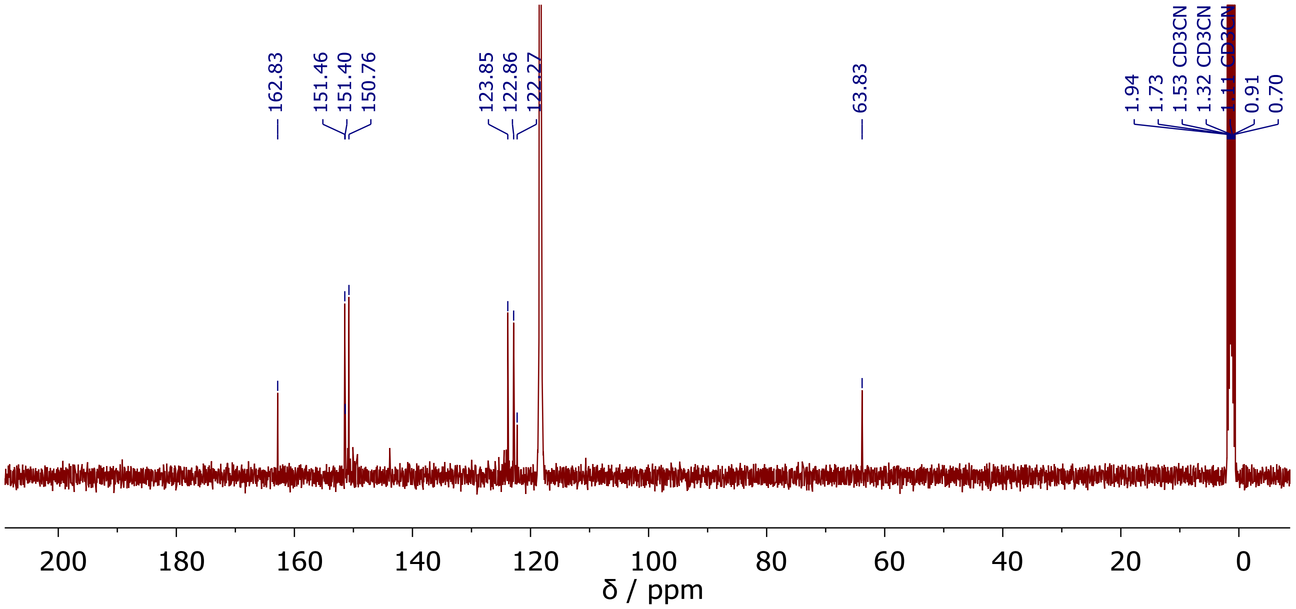

Figure S23. ^{13}C NMR (101 MHz, acetonitrile-d_3, 298 K) spectrum of 1-(pyridin-4-yl)-*N*-(pyridin-4-ylmethyl)methanimine (**2c**).

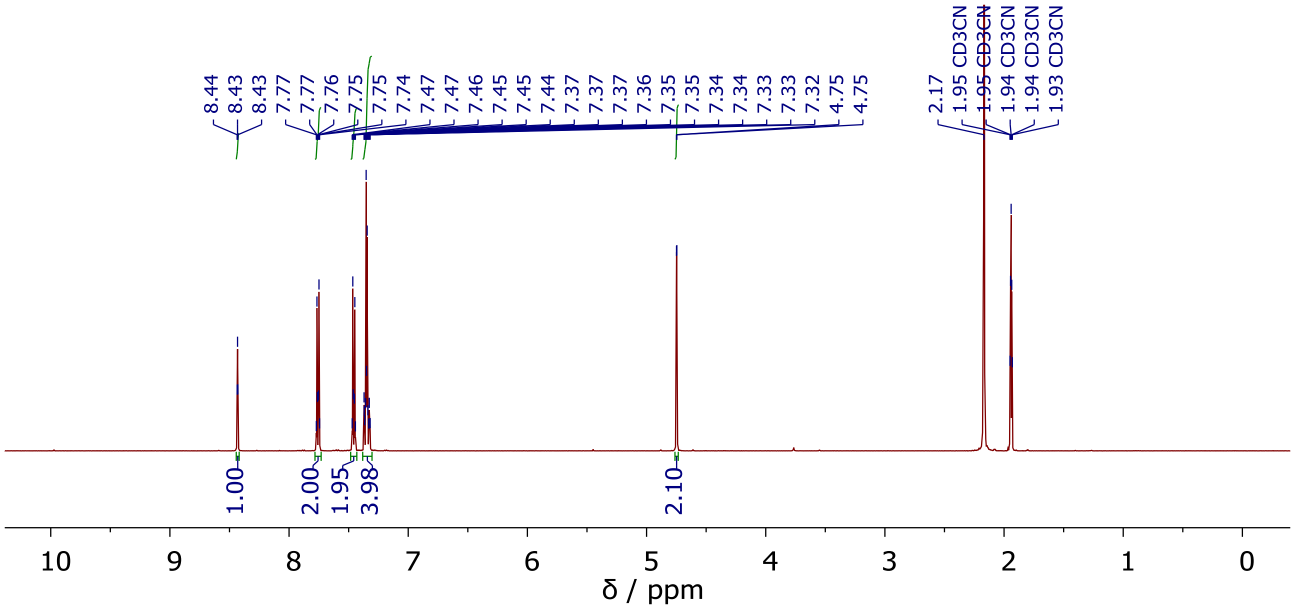

Figure S24. ^{1}H NMR (500 MHz, acetonitrile-d_3, 298 K) spectrum of N-(4-chlorobenzyl)-1-(4-chlorophenyl)methanimine (**2d**).

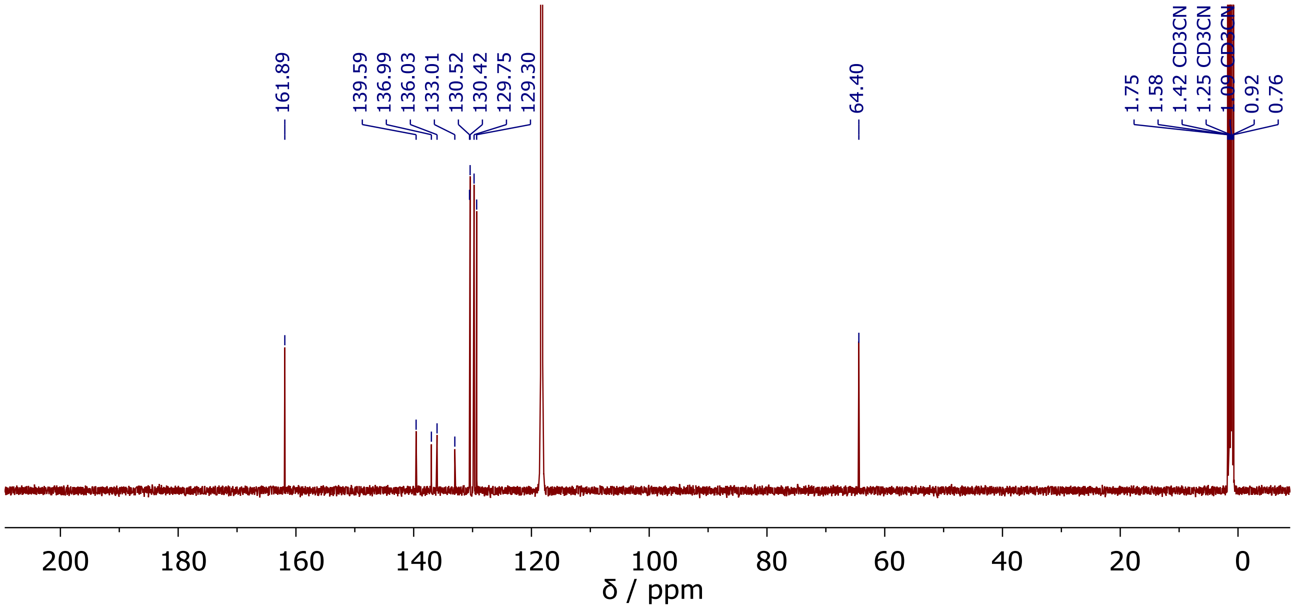

Figure S25. ^{13}C NMR (126 MHz, acetonitrile-d_3, 298 K) spectrum of N-(4-chlorobenzyl)-1-(4-chlorophenyl)methanimine (**2d**).

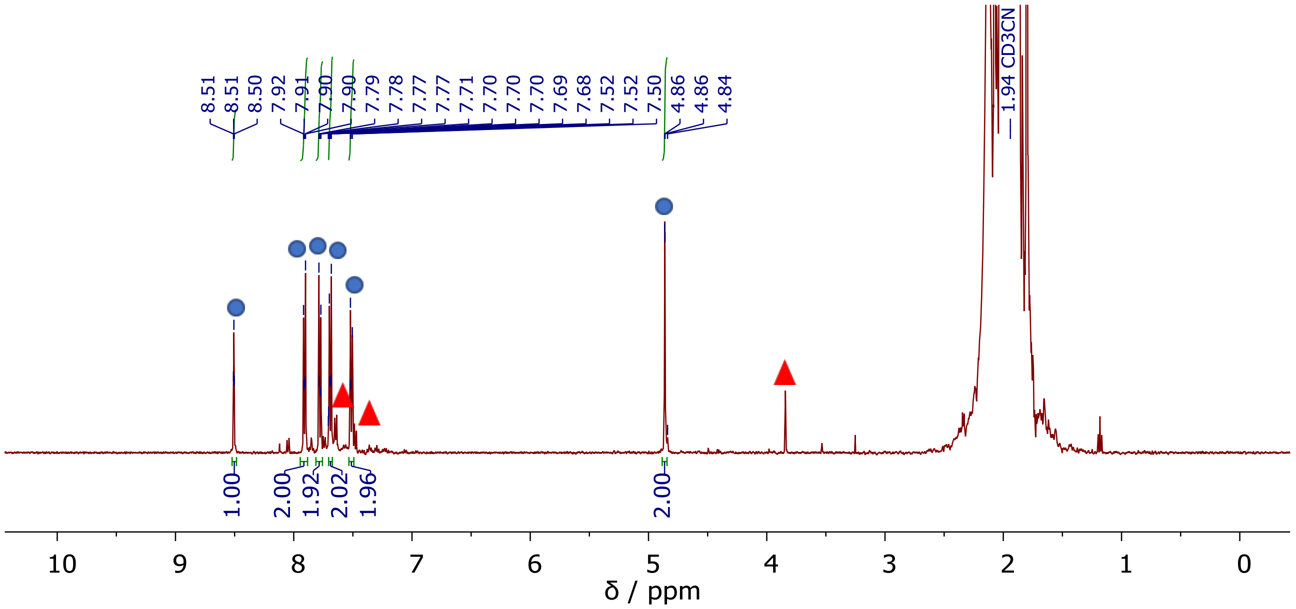

Figure S26. ^{1}H NMR (500 MHz, acetonitrile-d_3, 298 K) spectrum of 4-(((4-cyanobenzyl)imino)methyl)benzonitrile (**2e**). Blue circles (•) correspond to imine product **2e** and red triangles (▲) correspond to the remaining substrate **1e**.

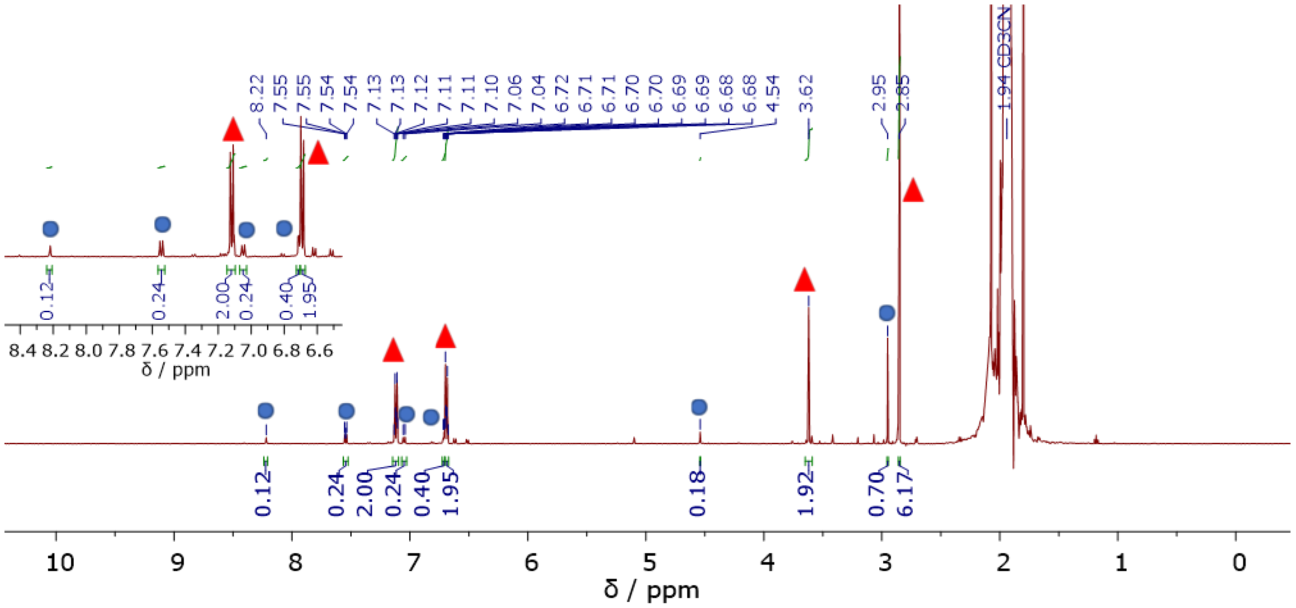

Figure S27. ^{1}H NMR (500 MHz, acetonitrile-d_3, 298 K) spectrum of 4-(((4-(dimethylamino)benzyl)imino)methyl)-N,N-dimethylaniline (**2g**). Blue circles (•) correspond to imine product **2g** and red triangles (▲) correspond to the remaining substrate **1g**.

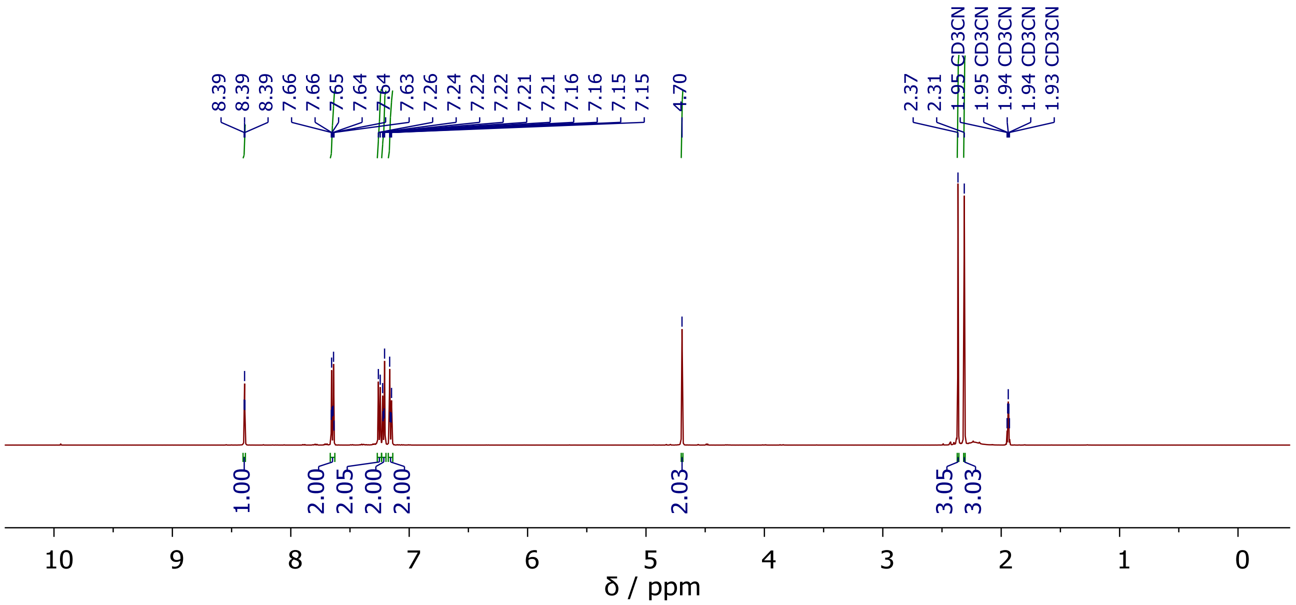

Figure S28. ^{1}H NMR (500 MHz, acetonitrile-d_3, 298 K) spectrum of N-(4-methylbenzyl)-1-(p-tolyl)methanimine (**2h**).

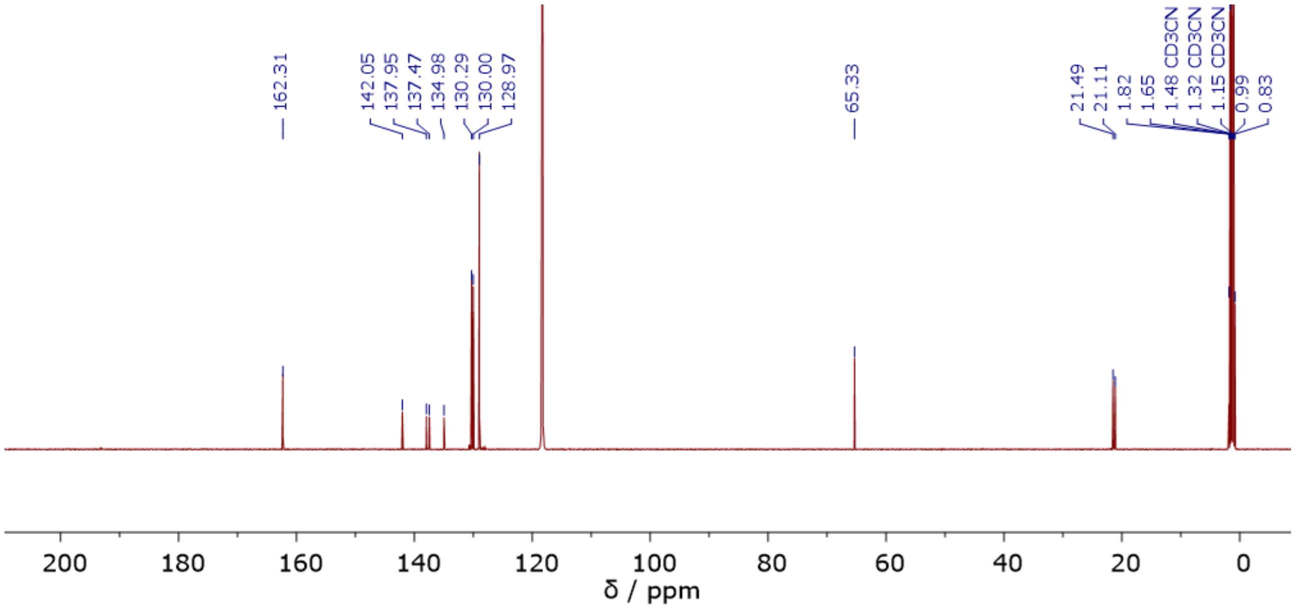

Figure S29. ^{13}C NMR (126 MHz, acetonitrile-d_3, 298 K) spectrum of N-(4-methylbenzyl)-1-(p-tolyl)methanimine (**2h**).

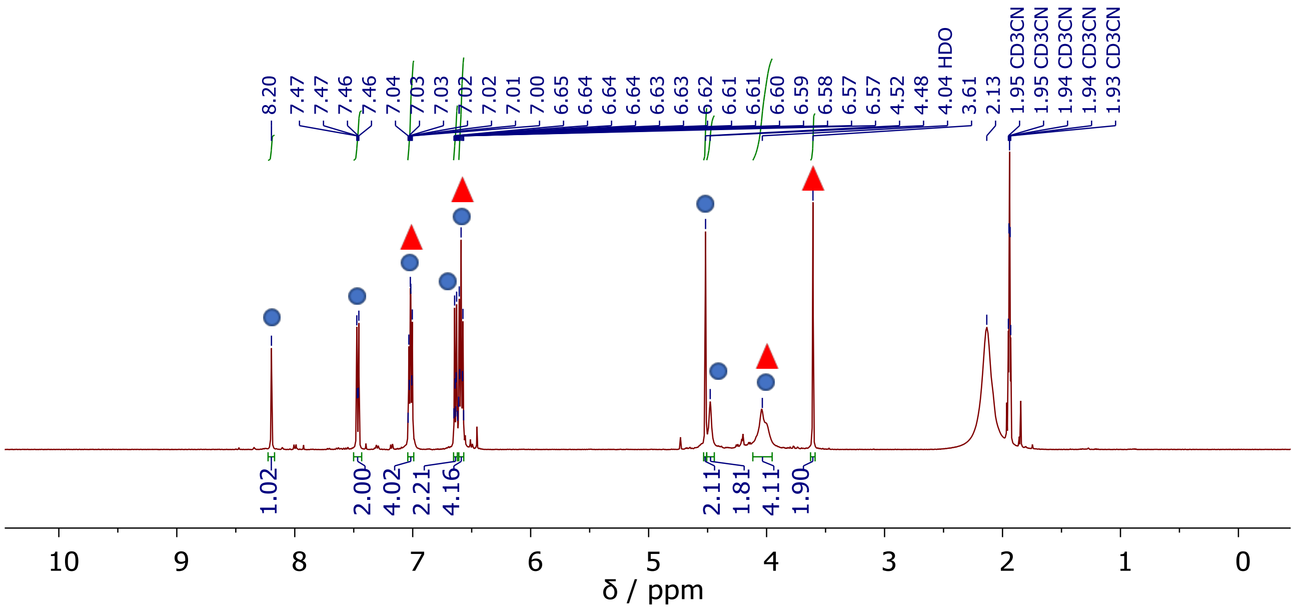

Figure S30. ^{1}H NMR (500 MHz, acetonitrile-d_3, 298 K) spectrum of 4-(((4-aminobenzyl)imino)methyl)aniline (**2i**). Blue circles (•) correspond to imine product **2i** and red triangles (▲) correspond to the remaining substrate **1i**.

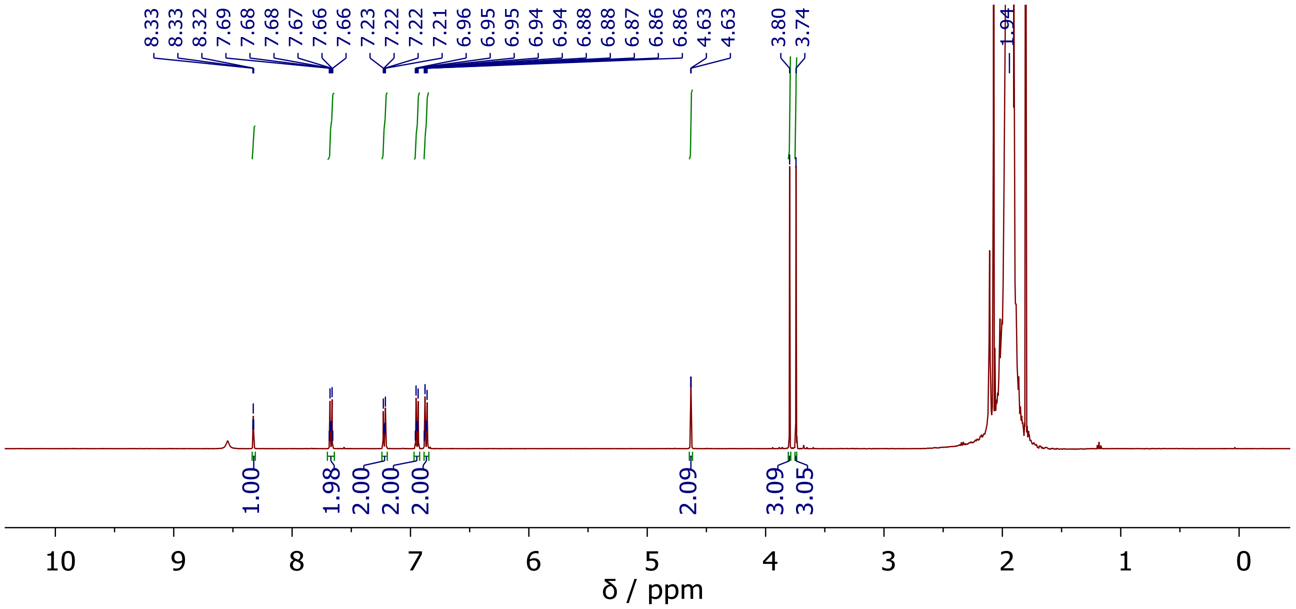

Figure S31. ^{1}H NMR (500 MHz, acetonitrile-d_3, 298 K) spectrum of N-(4-methoxybenzyl)-1-(4-methoxyphenyl)methanimine (**2j**).

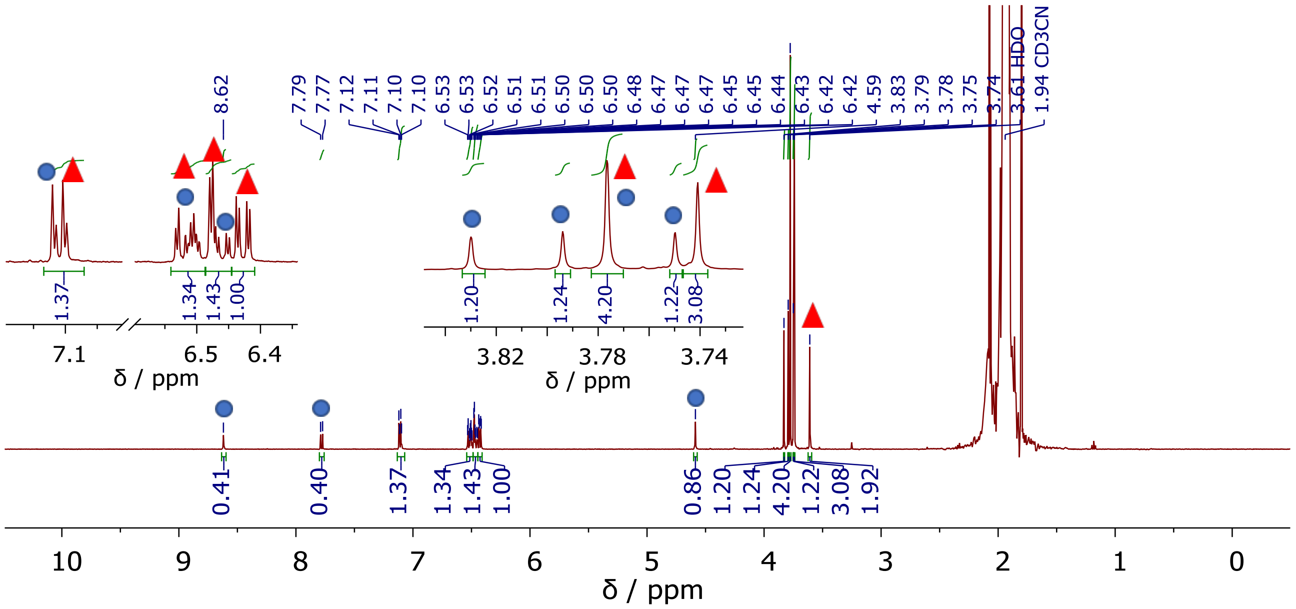

Figure S32. ^{1}H NMR (500 MHz, acetonitrile-d_3, 298 K) spectrum of N-(2,4-dimethoxybenzyl)-1-(2,4-dimethoxyphenyl)methanimine (**2k**). Blue circles (•) correspond to imine product **2k** and red triangles (▲) correspond to the remaining substrate **1k**.

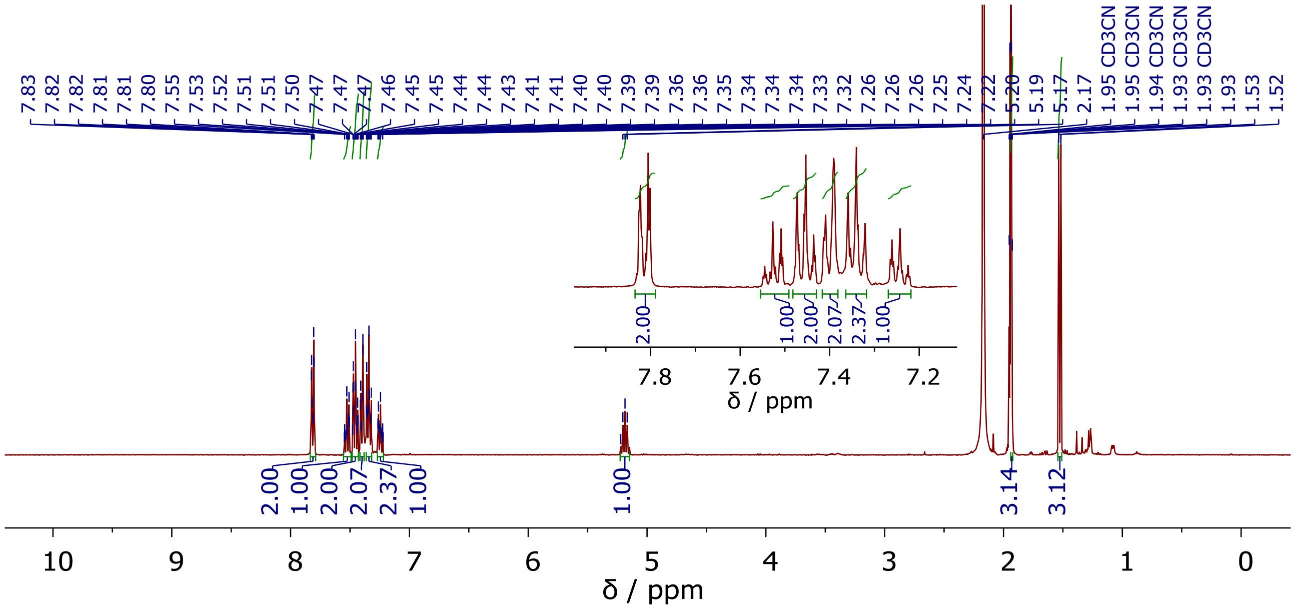

Figure S33. ^{1}H NMR (400 MHz, acetonitrile-d_3, 298 K) spectrum of 1-phenyl-*N*-(1-phenylethyl)ethan-1-imine (**2l**).

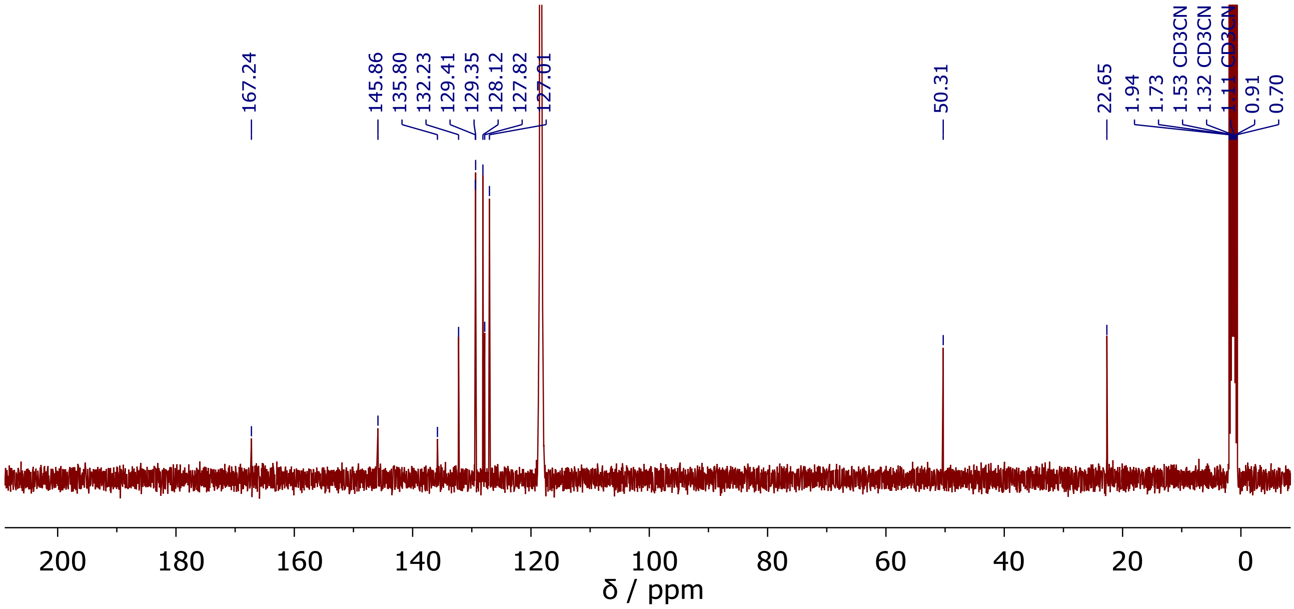

Figure S34. ^{13}C NMR (101 MHz, acetonitrile-d_3, 298 K) spectrum of 1-phenyl-*N*-(1-phenylethyl)ethan-1-imine (**2l**).

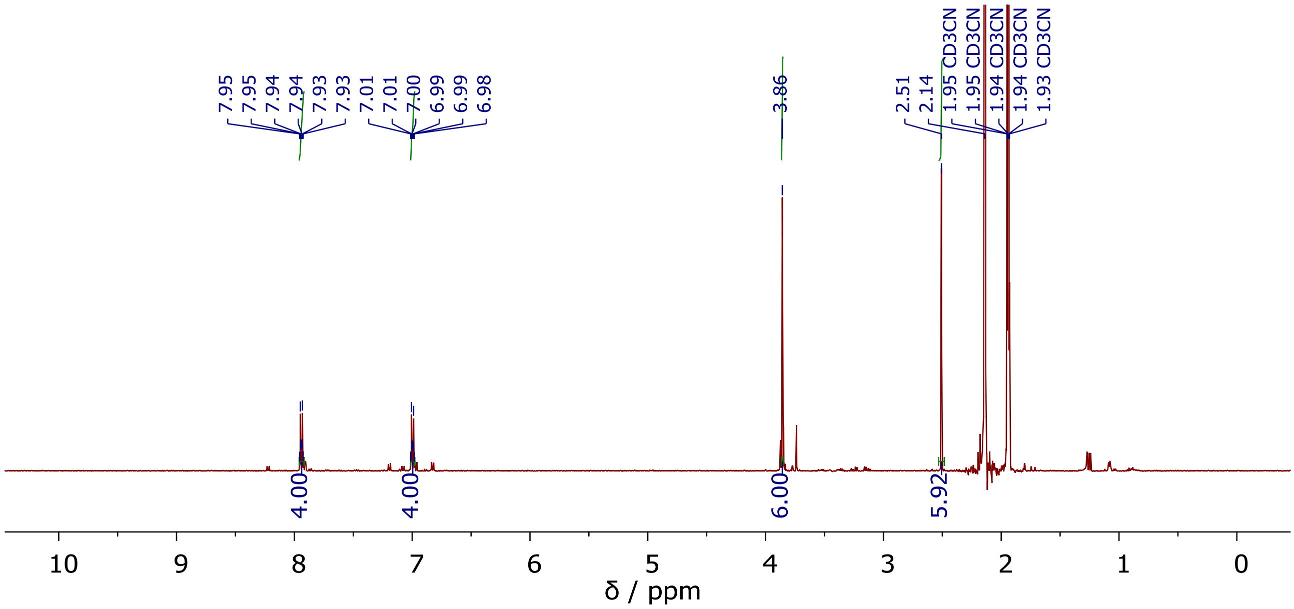

Figure S35. ^{1}H NMR (500 MHz, acetonitrile-d_3, 298 K) spectrum of 1-(4-methoxyphenyl)-N-(1-(4-methoxyphenyl)ethyl)ethan-1-imine (**2m**).

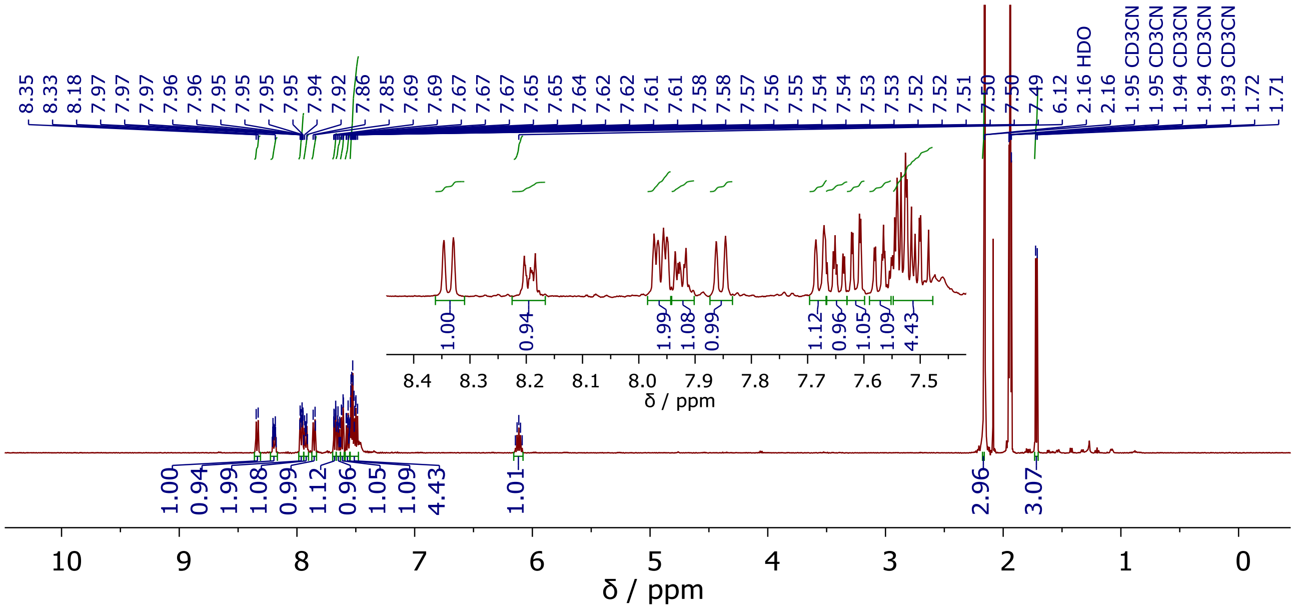

Figure S36. ^{1}H NMR (500 MHz, acetonitrile-d_3, 298 K) spectrum of 1-(naphthalen-2-yl)-N-(1-(naphthalen-2-yl)ethyl)ethan-1-imine (**2n**).

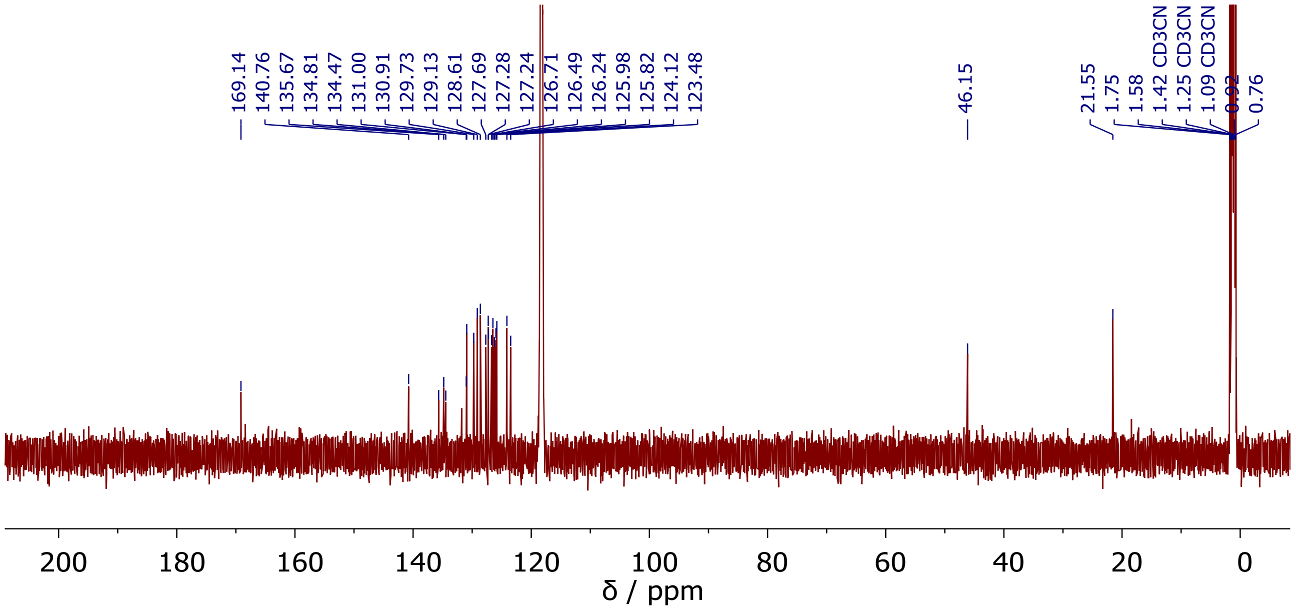

Figure S37. ^{13}C NMR (126 MHz, acetonitrile-*d*$_3$, 298 K) spectrum of 1-(naphthalen-2-yl)-N-(1-(naphthalen-2-yl)ethyl)ethan-1-imine (**2n**).

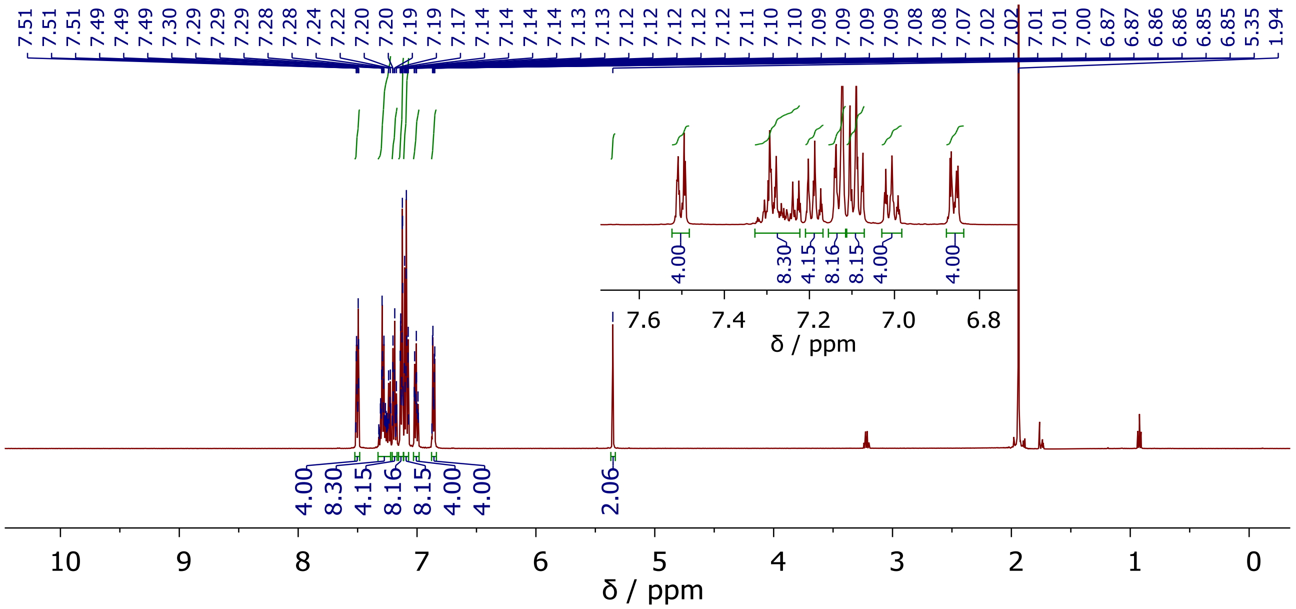

Figure S38. ^{1}H NMR (500 MHz, acetonitrile-*d*$_3$, 298 K) spectrum of N-benzhydryl-1,1-diphenylmethanimine (**2o**).

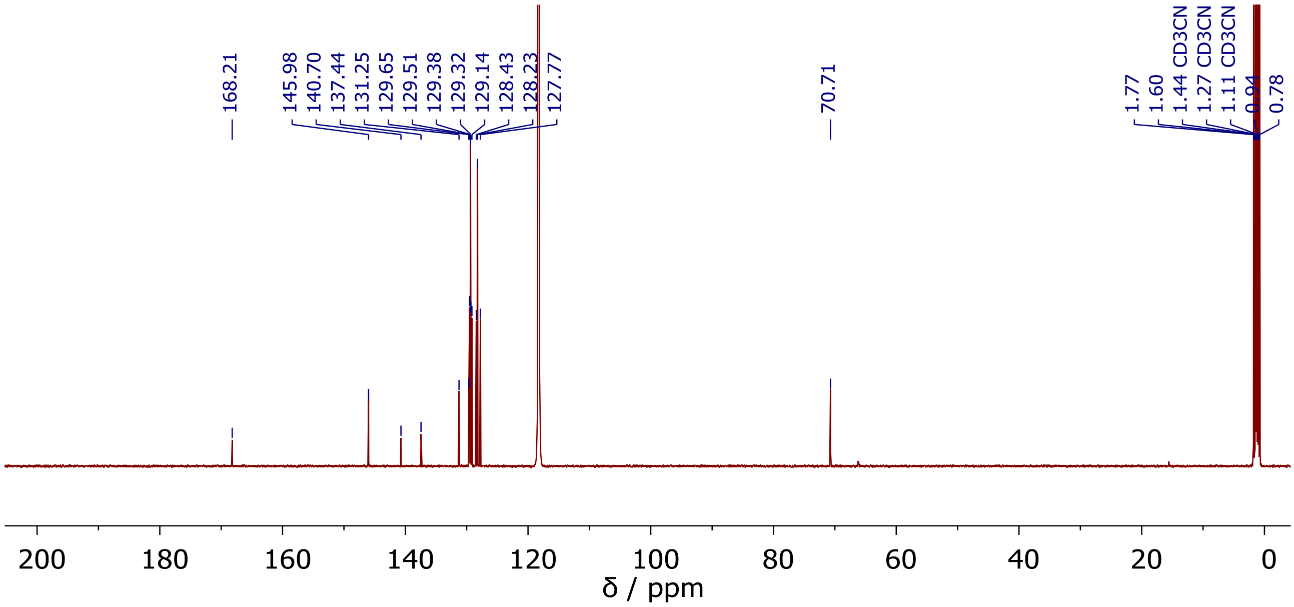

Figure S39. ^{13}C NMR (126 MHz, acetonitrile-d_3, 298 K) spectrum of N-benzhydryl-1,1-diphenylmethanimine (**2o**).

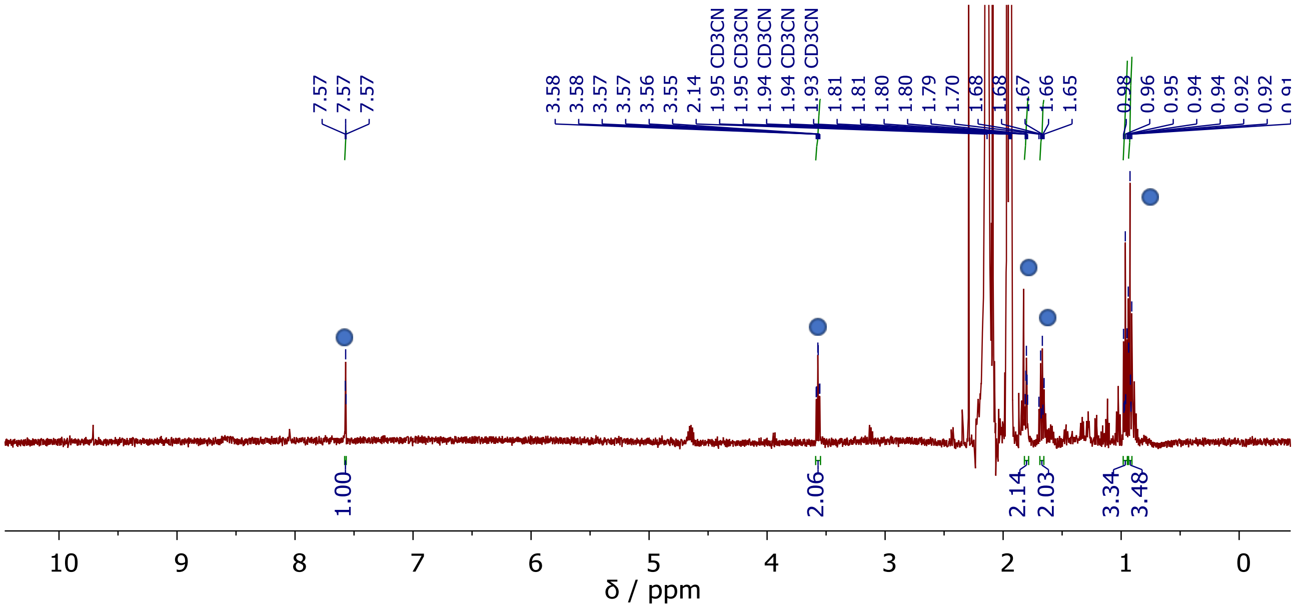

Figure S40. ^{1}H NMR (500 MHz, acetonitrile-d_3, 298 K) spectrum of *N*-propylpropan-1-imine (**2q**). Blue circles (•) correspond to the imine product.

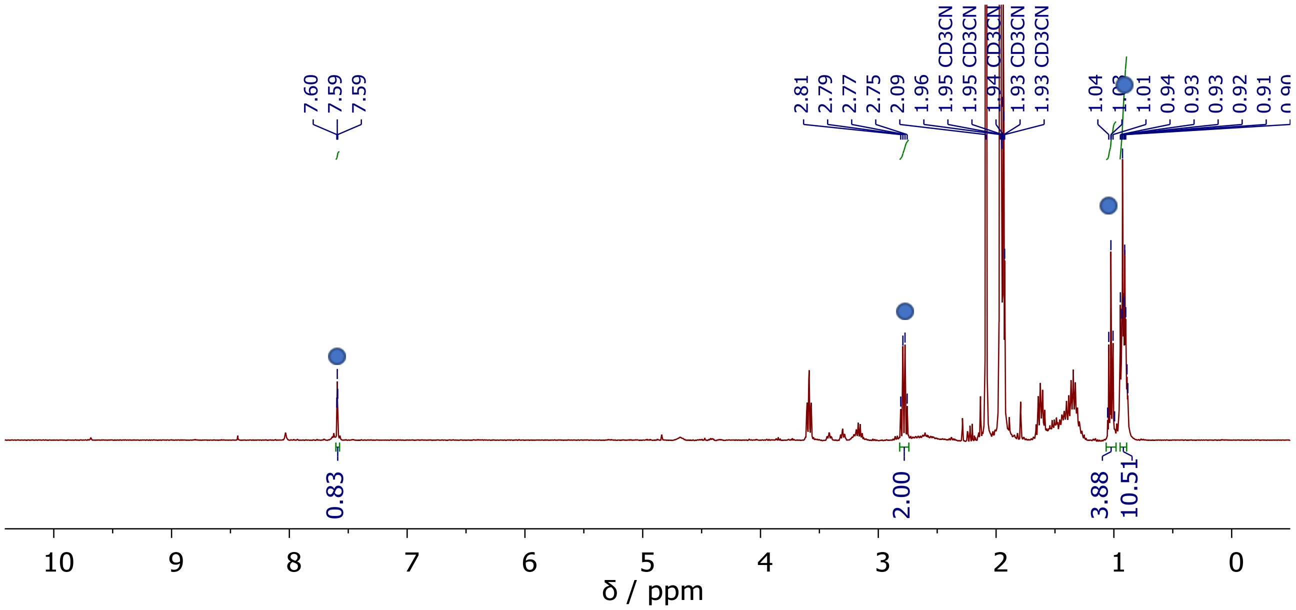

Figure S41. ^{1}H NMR (400 MHz, acetonitrile-d_3, 298 K) spectrum of *N*-butylbutan-1-imine (**2r**). Blue circles (•) correspond to the imine product.

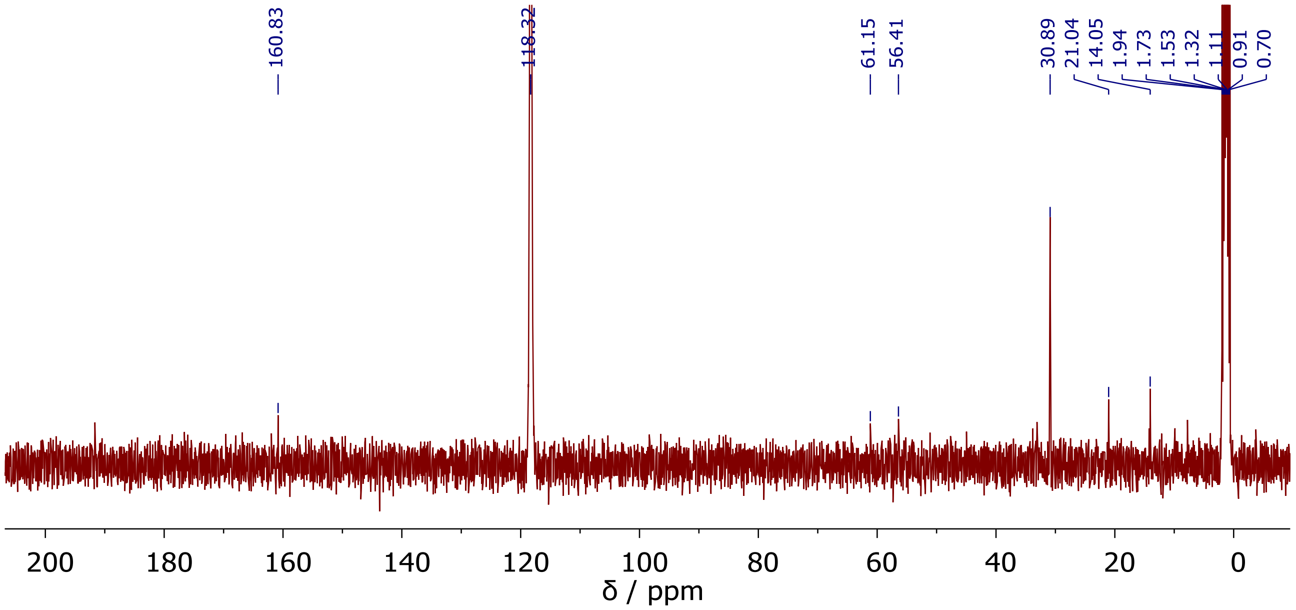

Figure S42. ^{13}C NMR (101 MHz, acetonitrile-d_3, 298 K) spectrum of *N*-butylbutan-1-imine (**2r**).

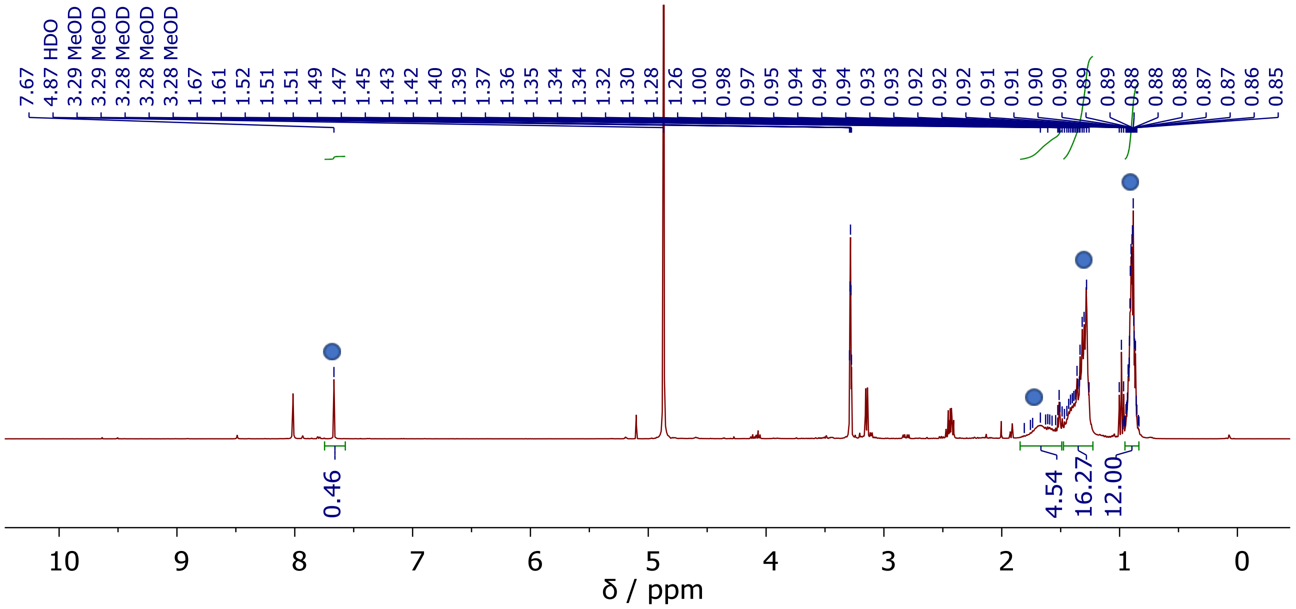

Figure S43. ^{1}H NMR (400 MHz, CD_3OD, 298 K) spectrum of 2-ethyl-*N*-(2-ethylhexyl)hexan-1-imine (**2s**). Blue circles (•) correspond to the imine product.

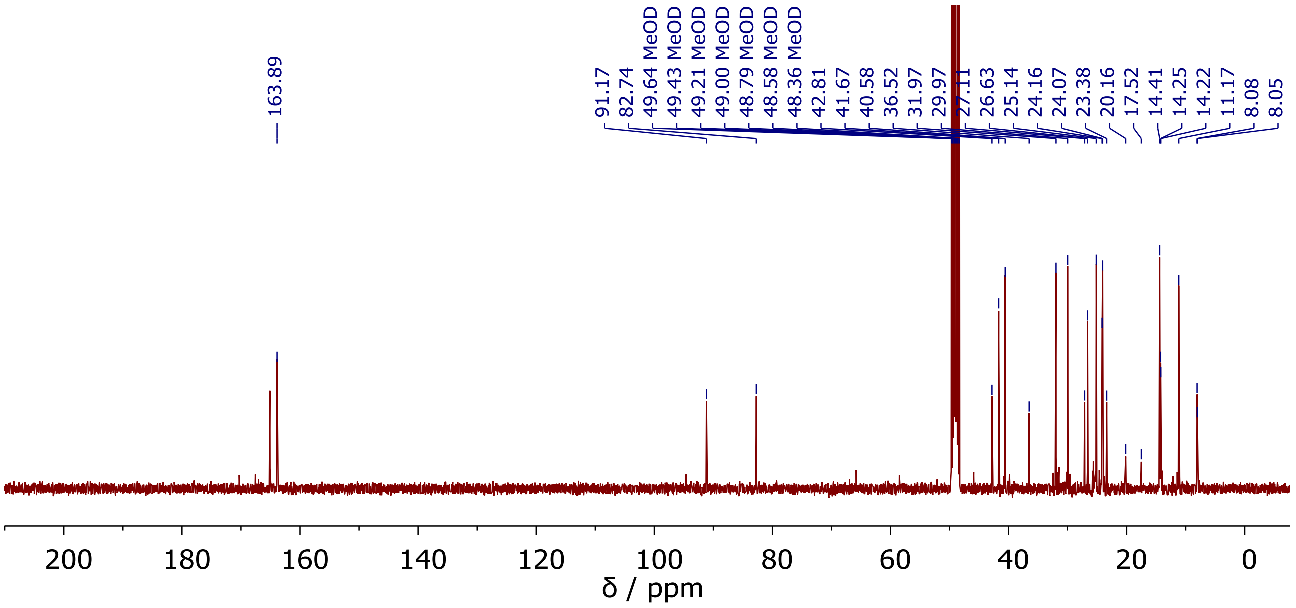

Figure S44. ^{13}C NMR (101 MHz, CD_3OD, 298 K) spectrum of 2-ethyl-*N*-(2-ethylhexyl)hexan-1-imine (**2s**).

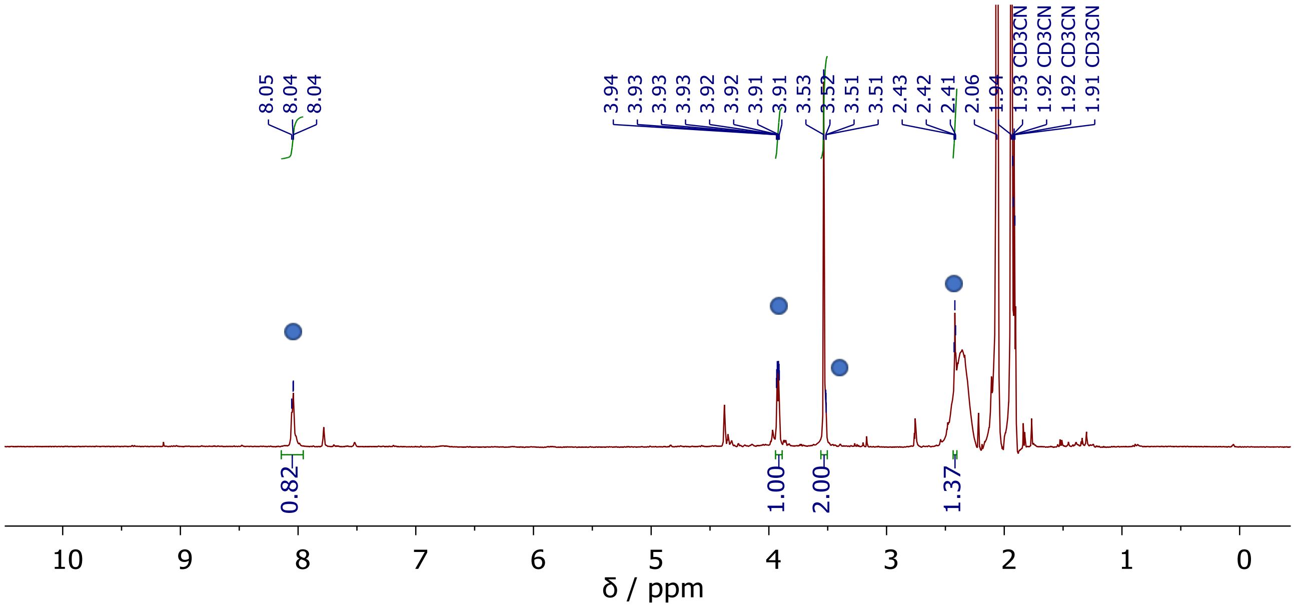

Figure S45. ^{1}H NMR (400 MHz, acetonitrile-d_3, 298 K) spectrum of *N*-(prop-2-yn-1-yl)prop-2-yn-1-imine (**2t**). Blue circles (•) correspond to the imine product **2t**.

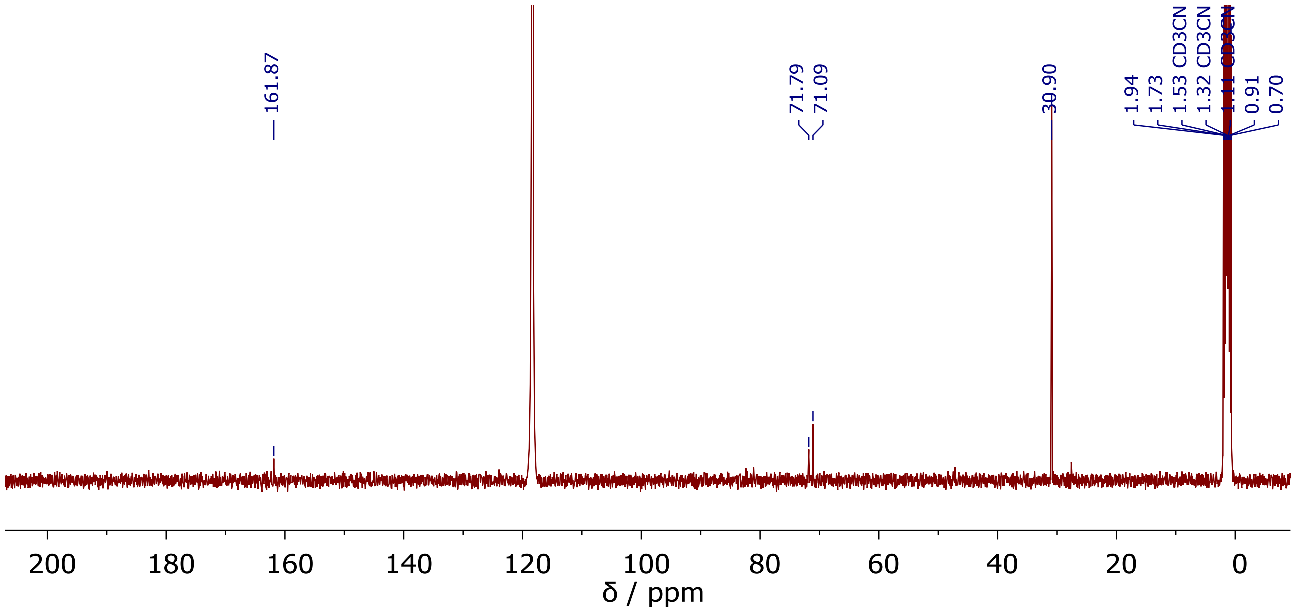

Figure S46. ^{13}C NMR (101 MHz, acetonitrile-d_3, 298 K) spectrum of *N*-(prop-2-yn-1-yl)prop-2-yn-1-imine (**2t**).

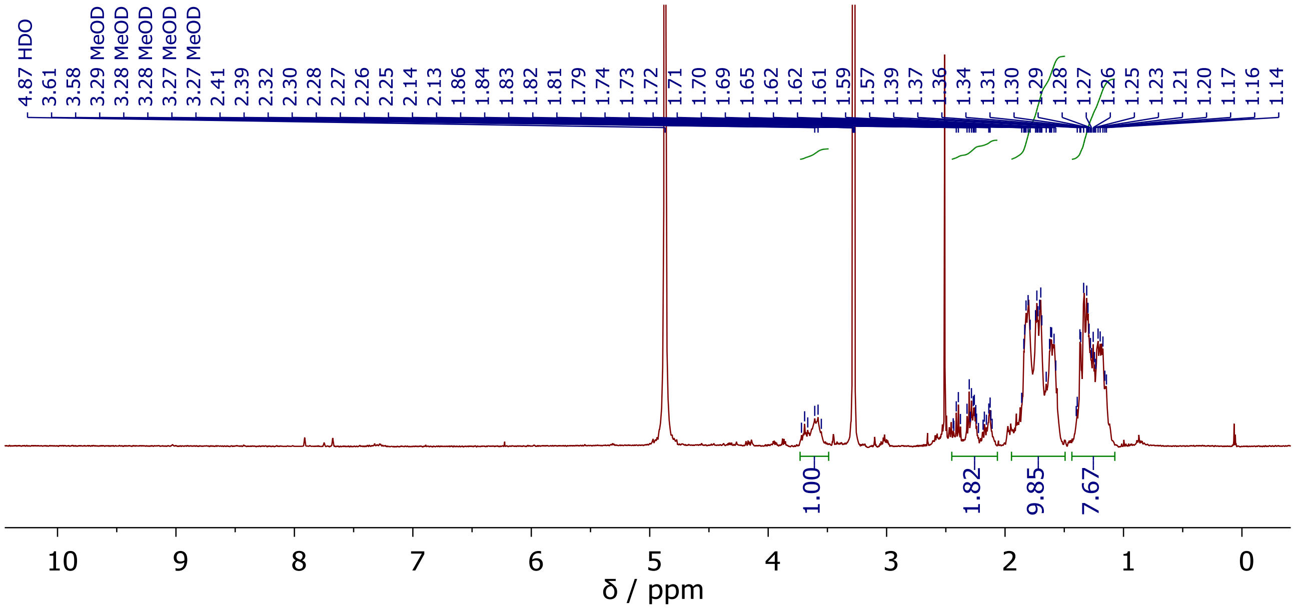

Figure S47. ^{1}H NMR (400 MHz, CD_3OD, 298 K) spectrum of N-cyclohexylcyclohexanimine (**2u**).

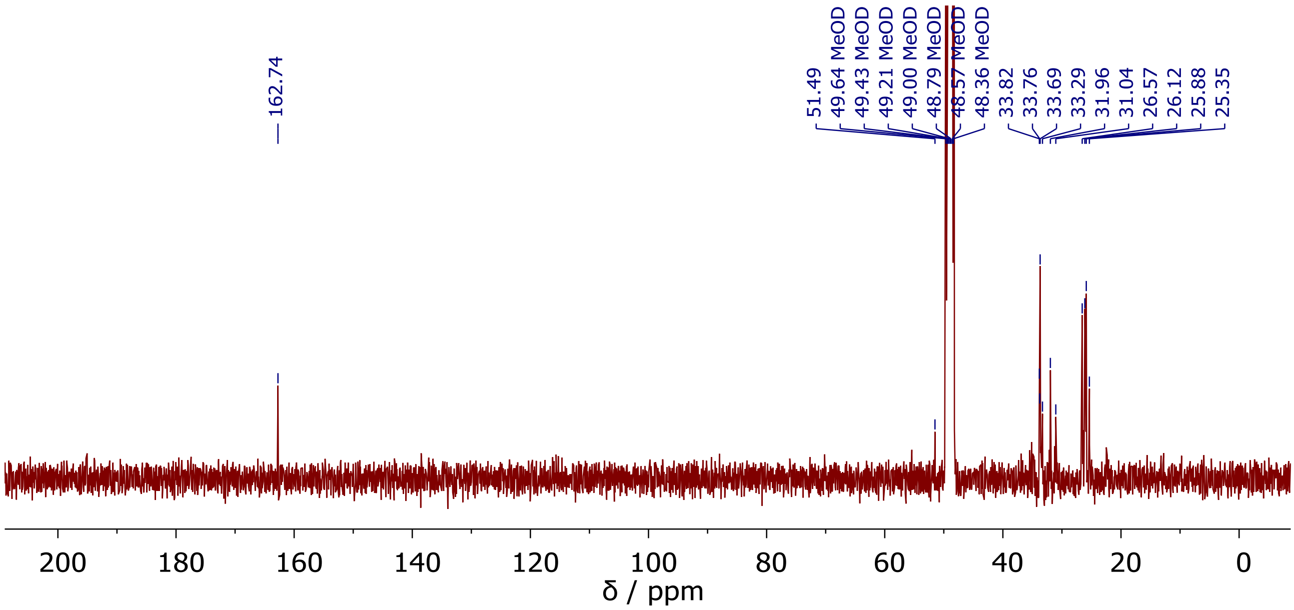

Figure S48. ^{13}C NMR (101 MHz, CD_3OD, 298 K) spectrum of N-cyclohexylcyclohexanimine (**2u**).

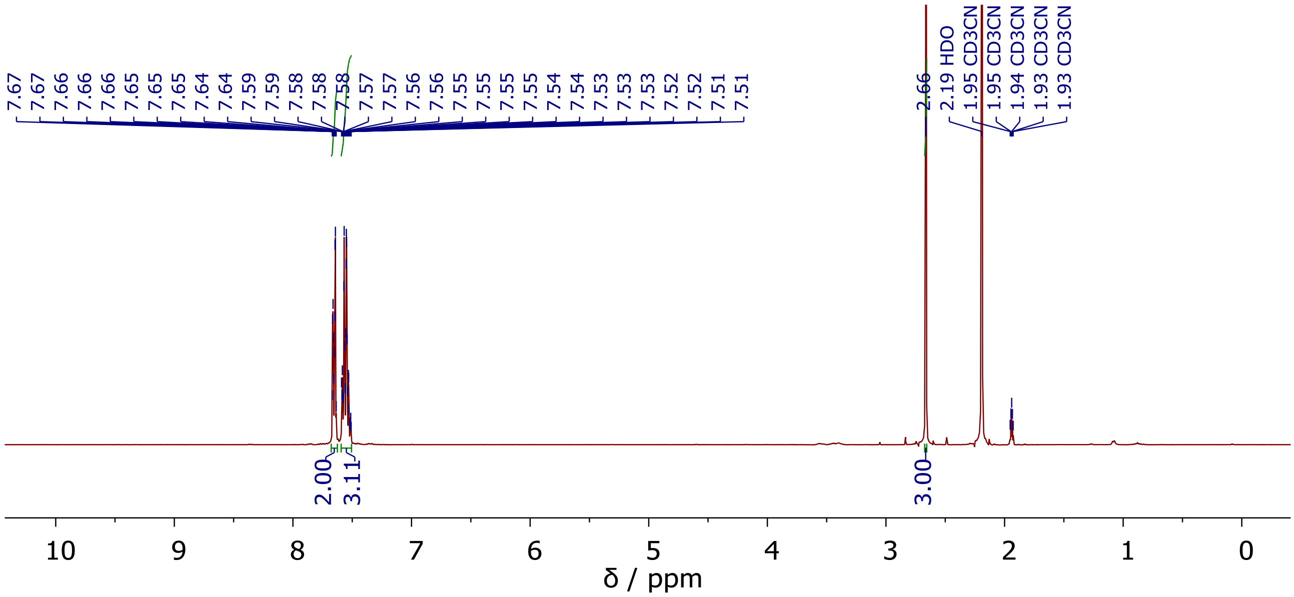

Figure S49. ^{1}H NMR (400 MHz, acetonitrile-d_3, 298 K) spectrum of (methylsulfinyl)benzene (**4a**).

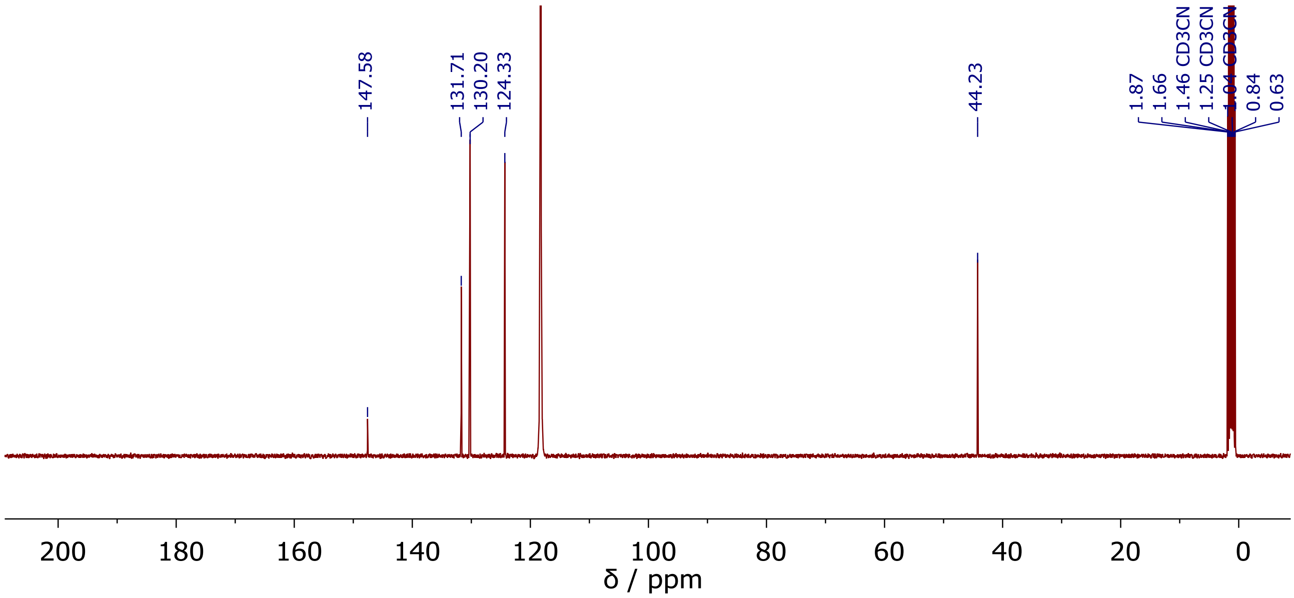

Figure S50. ^{13}C NMR (101 MHz, acetonitrile-d_3, 298 K) spectrum of (methylsulfinyl)benzene (**4a**).

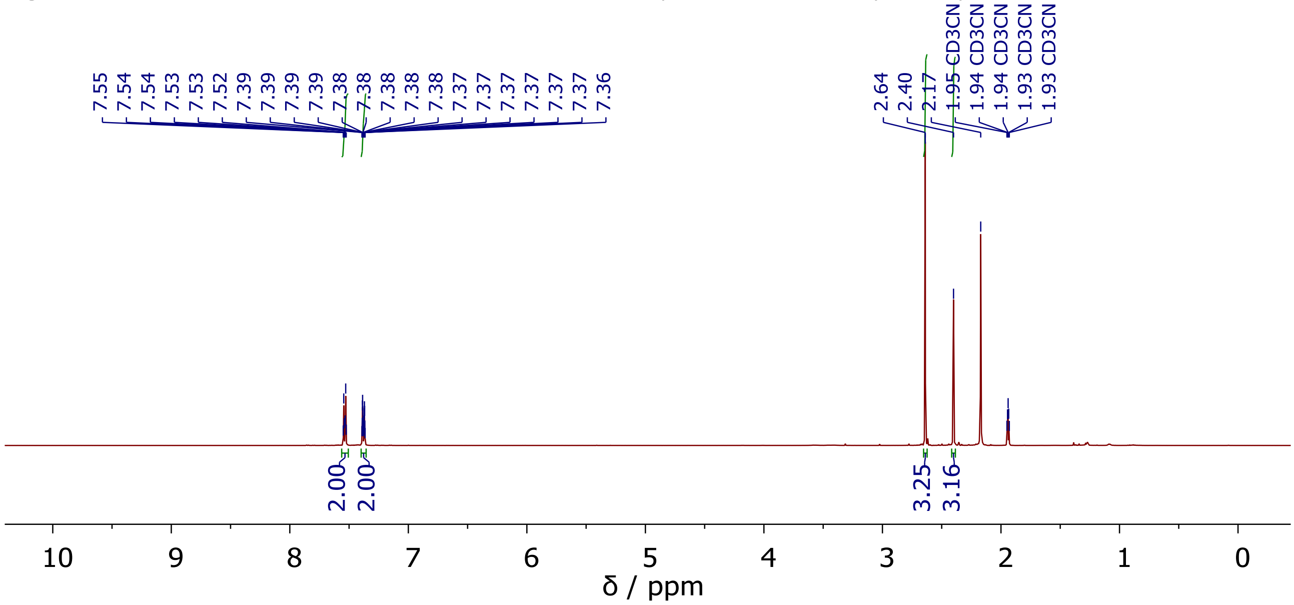

Figure S51. ^{1}H NMR (400 MHz, acetonitrile-d_3, 298 K) spectrum of 1-methyl-4-(methylsulfinyl)benzene (**4b**).

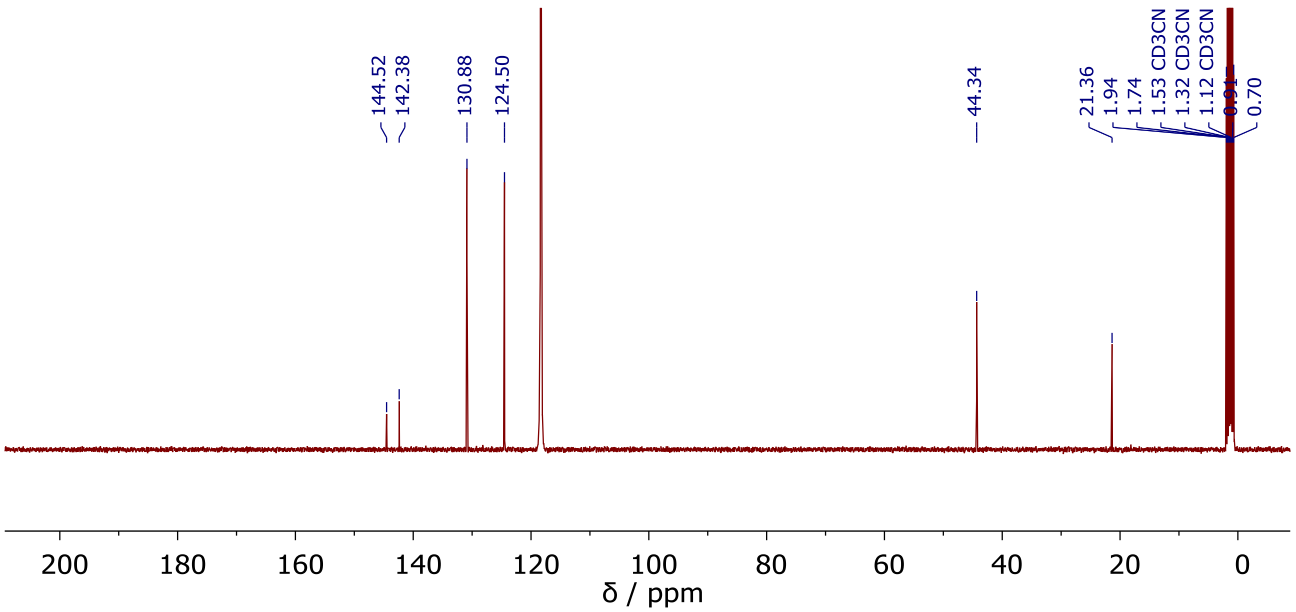

Figure S52. ^{13}C NMR (101 MHz, acetonitrile-d_3, 298 K) spectrum of 1-methyl-4-(methylsulfinyl)benzene (**4b**).

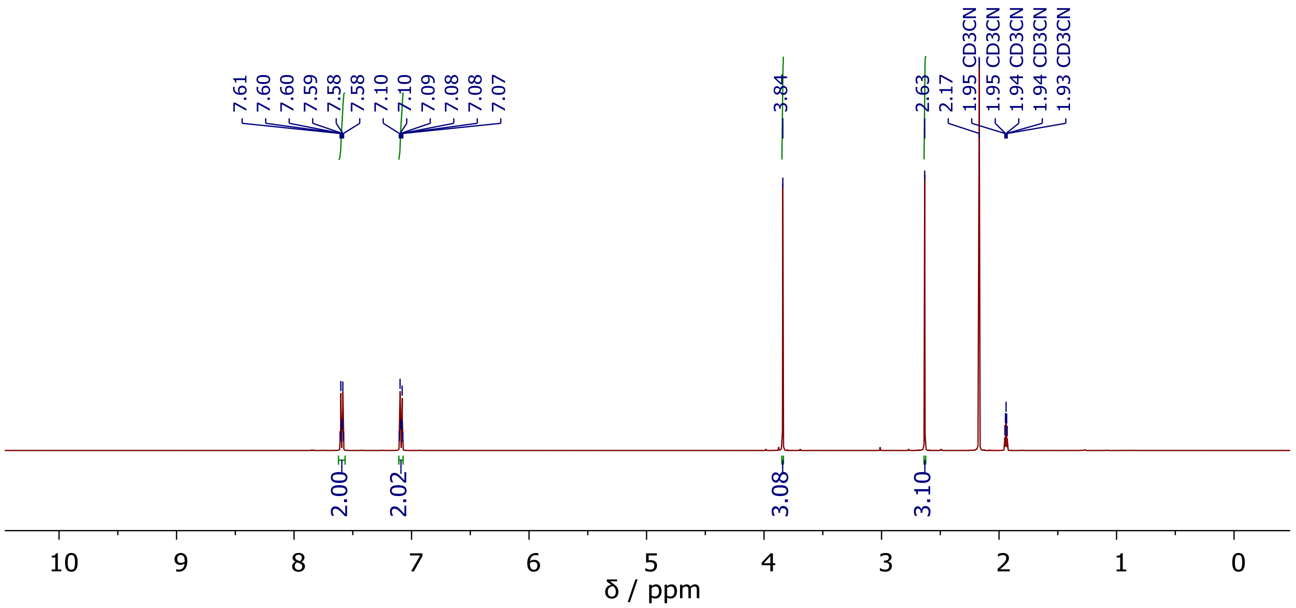

Figure S53. ^{1}H NMR (500 MHz, acetonitrile-d_3, 298 K) spectrum of 1-methoxy-4-(methylsulfinyl)benzene (**4c**).

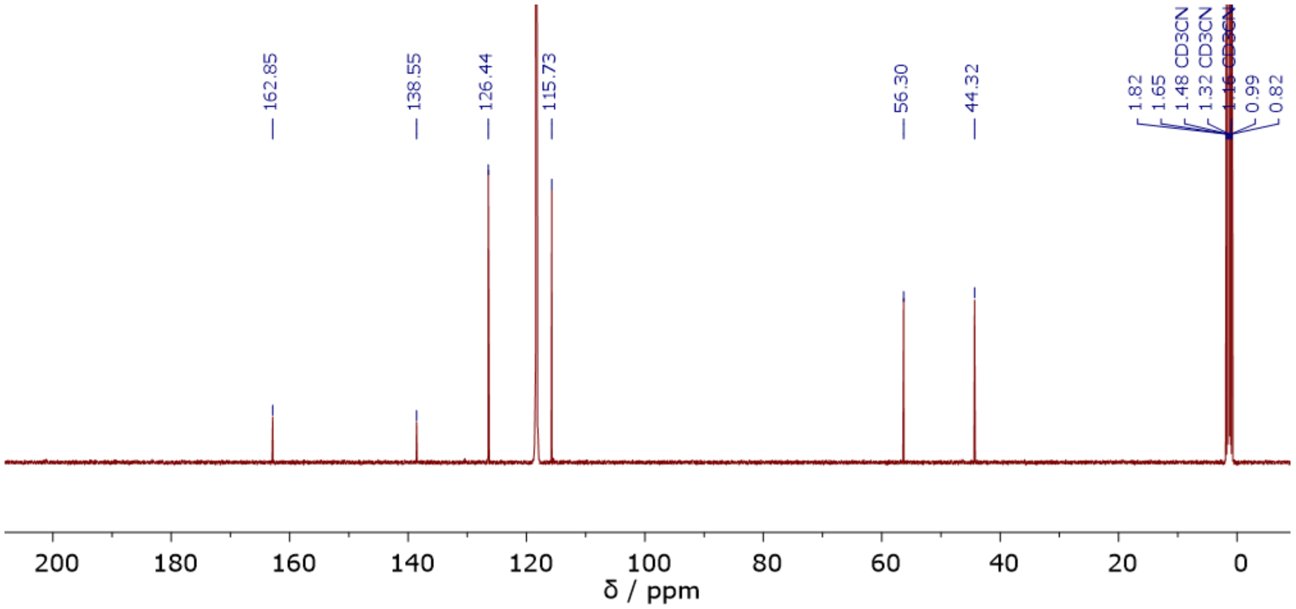

Figure S54. ^{13}C NMR (126 MHz, acetonitrile-d_3, 298 K) spectrum of 1- methoxy -4-(methylsulfinyl)benzene (**4c**).

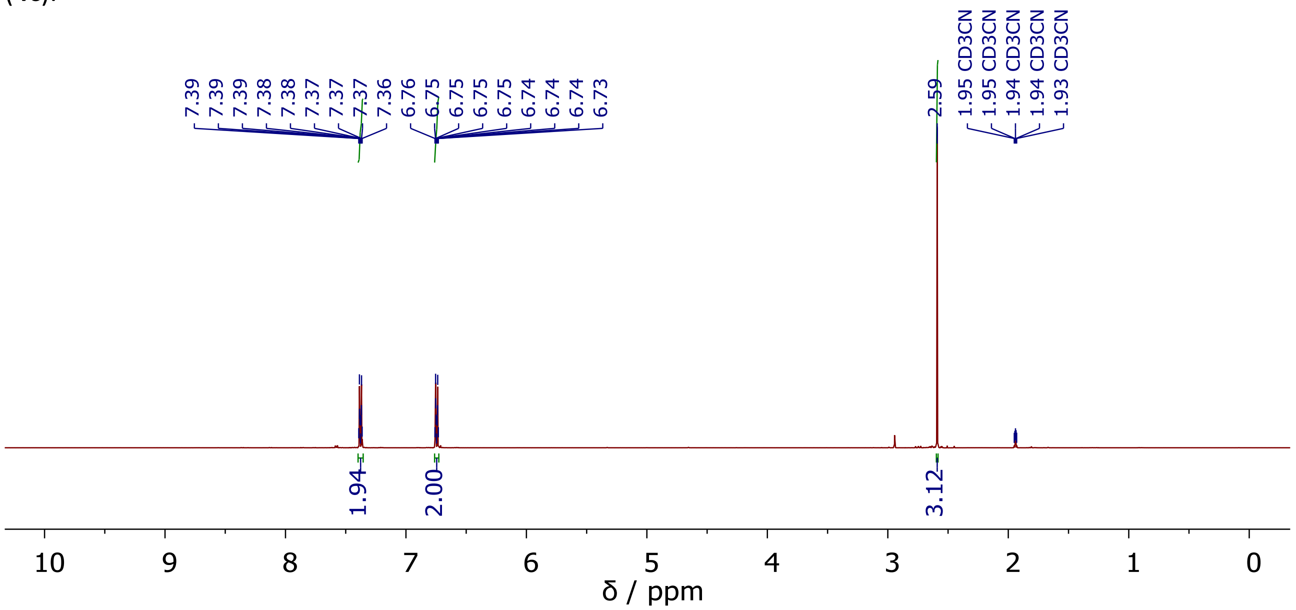

Figure S55. ^{1}H NMR (500 MHz, acetonitrile-d_3, 298 K) spectrum of 4-(methylsulfinyl)aniline (**4d**).

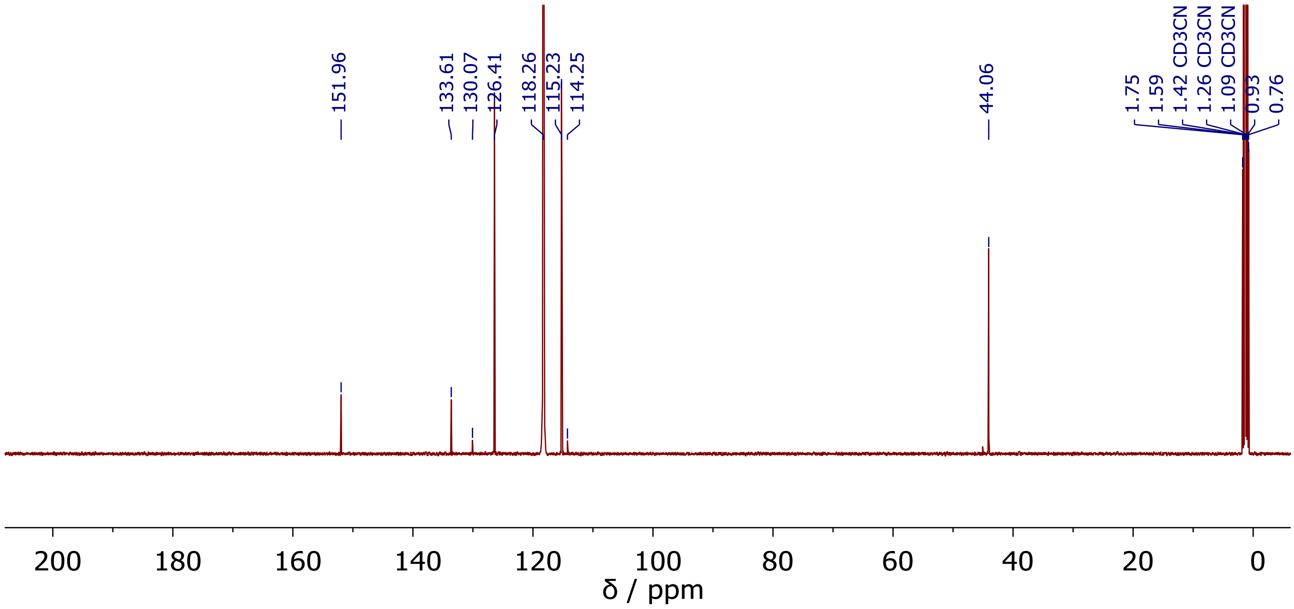

Figure S56. ^{13}C NMR (126 MHz, acetonitrile-d_3, 298 K) spectrum of 4-(methylsulfinyl)aniline (**4d**).

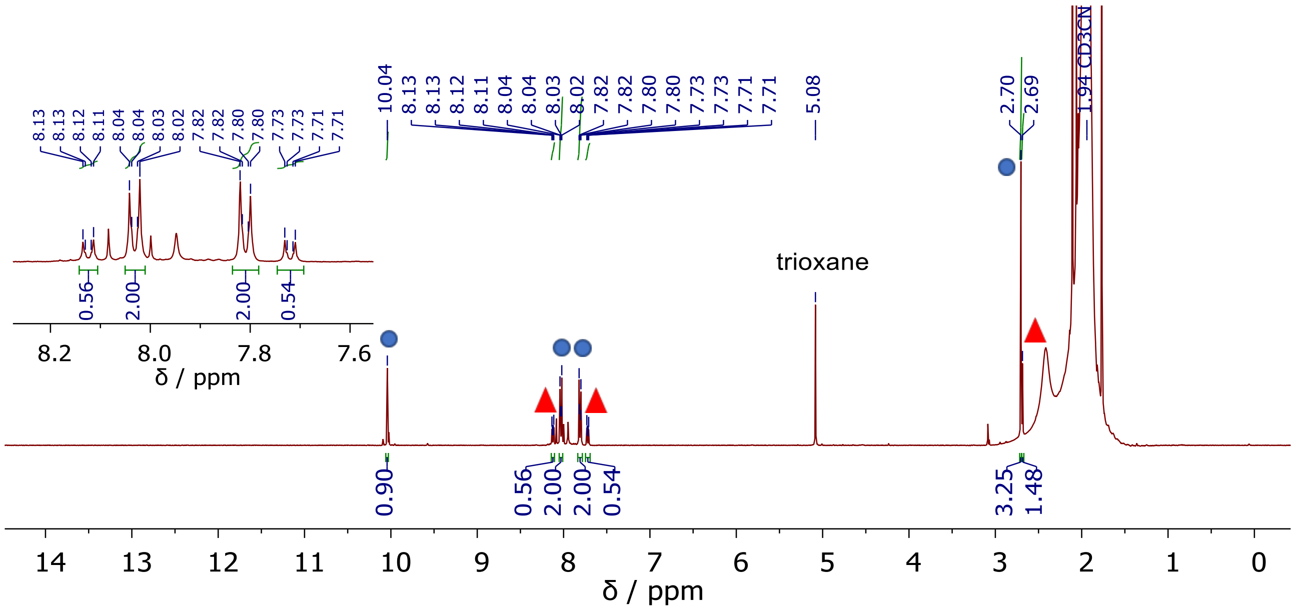

Figure S57. ^1H NMR (400 MHz, acetonitrile-d_3, 298 K) spectrum of 4-(methylsulfinyl)benzaldehyde (**4e**). Blue circles (•) correspond to sulfoxide **4e**. Red triangles (▲) correspond to the major over-oxidation product.

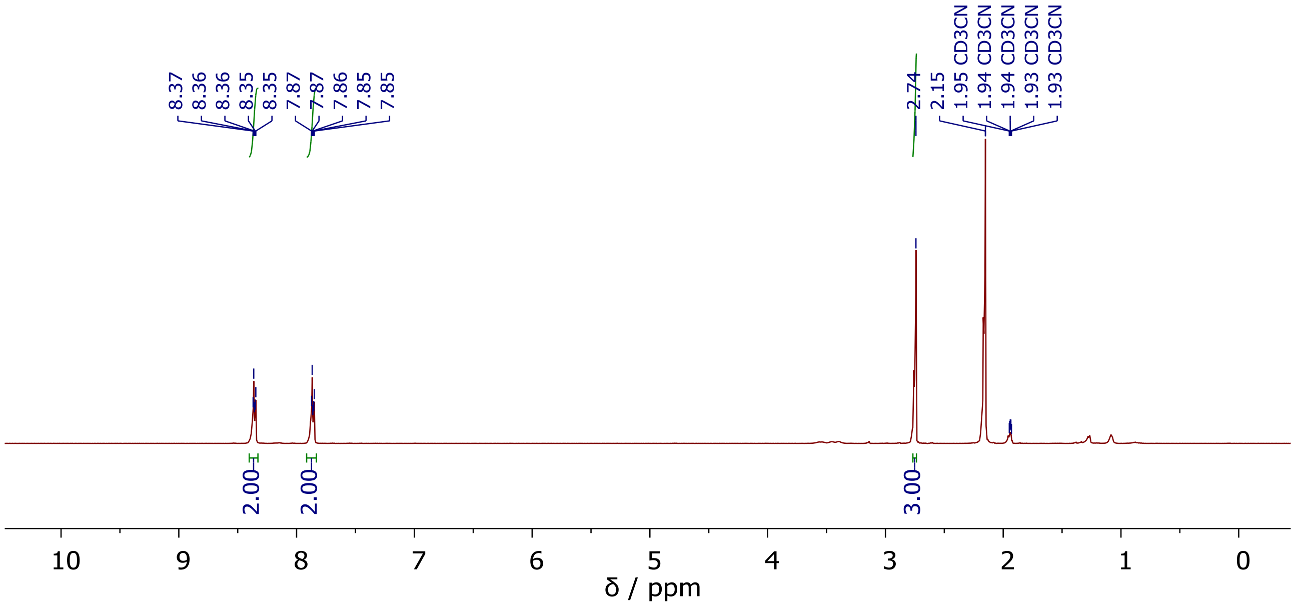

Figure S58. ^{1}H NMR (500 MHz, acetonitrile-d_3, 298 K) spectrum of 1-(methylsulfinyl)-4-nitrobenzene (**4f**).

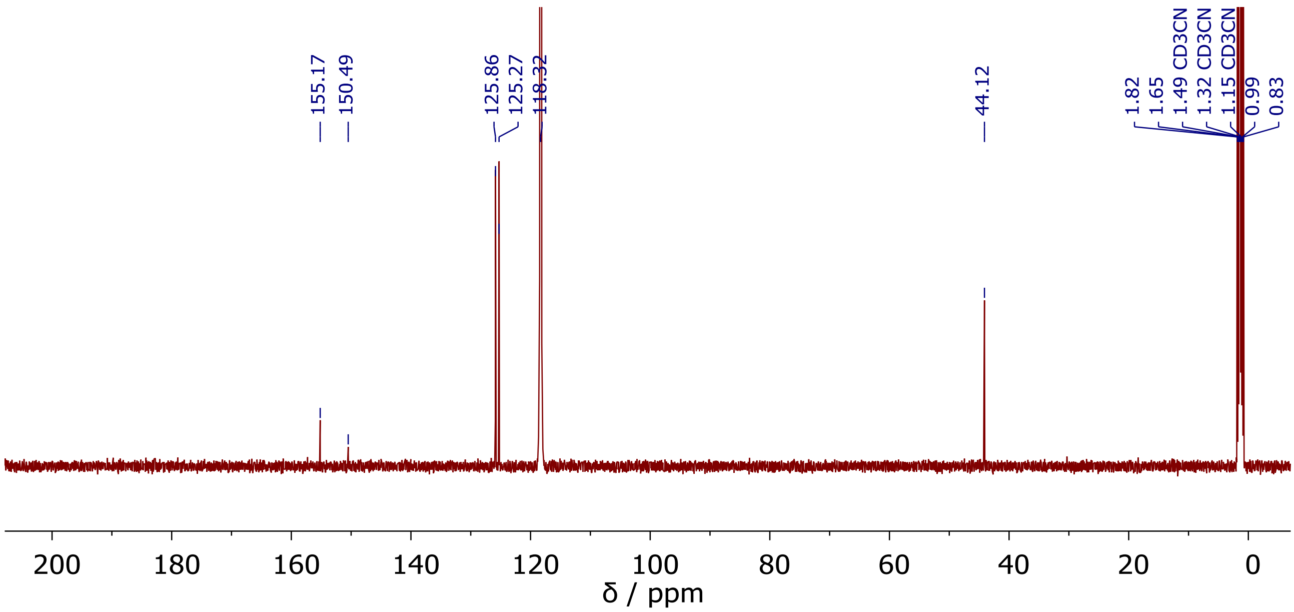

Figure S59. ^{13}C NMR (126 MHz, acetonitrile-d_3, 298 K) spectrum of 1-(methylsulfinyl)-4-nitrobenzene (**4f**).

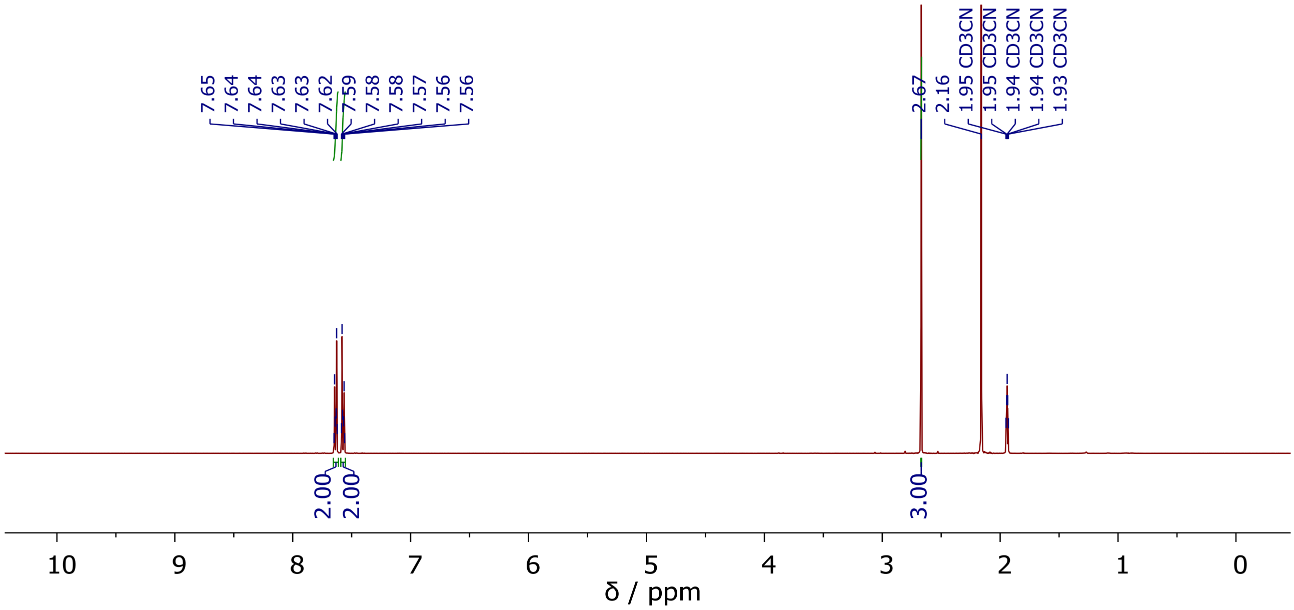

Figure S60. ^{1}H NMR (500 MHz, acetonitrile-d_3, 298 K) spectrum of 1-chloro-4-(methylsulfinyl)benzene (**4g**).

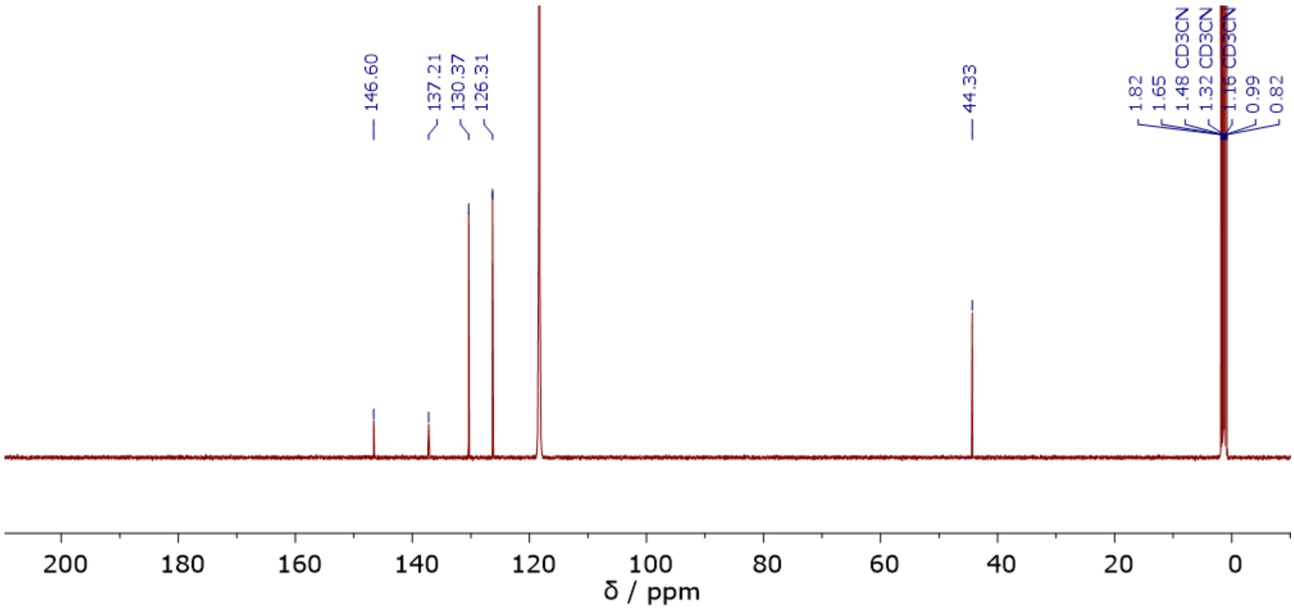

Figure S61. ^{13}C NMR (126 MHz, acetonitrile-d_3, 298 K) spectrum of 1-chloro-4-(methylsulfinyl)benzene (**4g**).

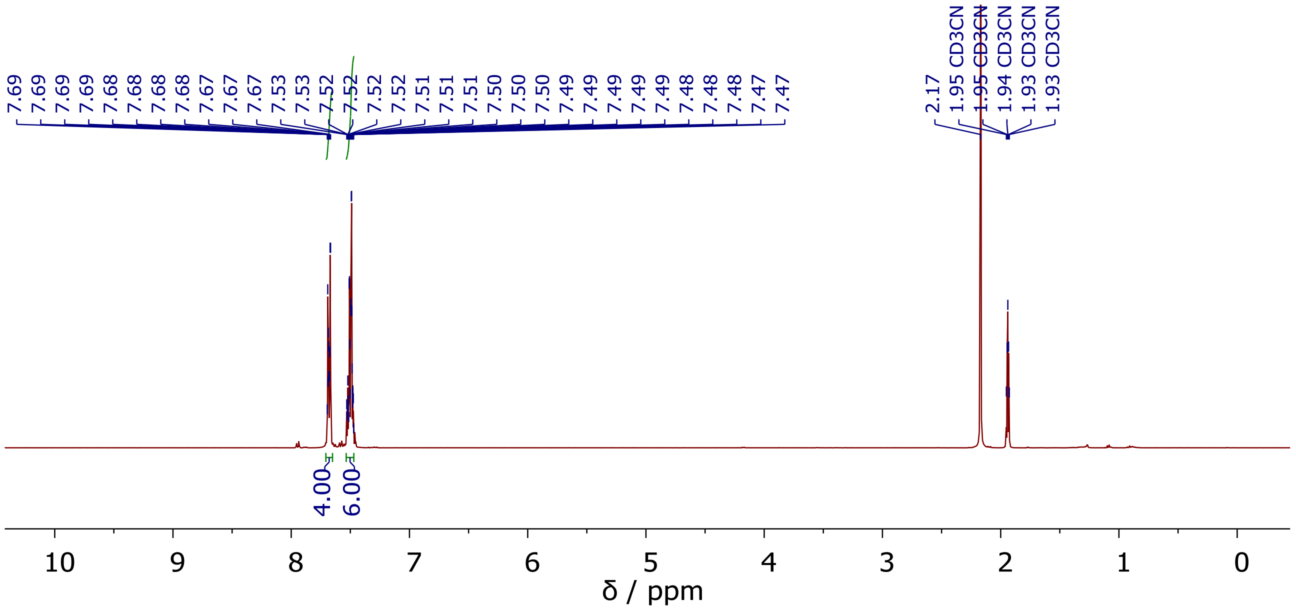

Figure S62. ^{1}H NMR (400 MHz, acetonitrile-d_3, 298 K) spectrum of diphenyl sulfoxide (**4i**).

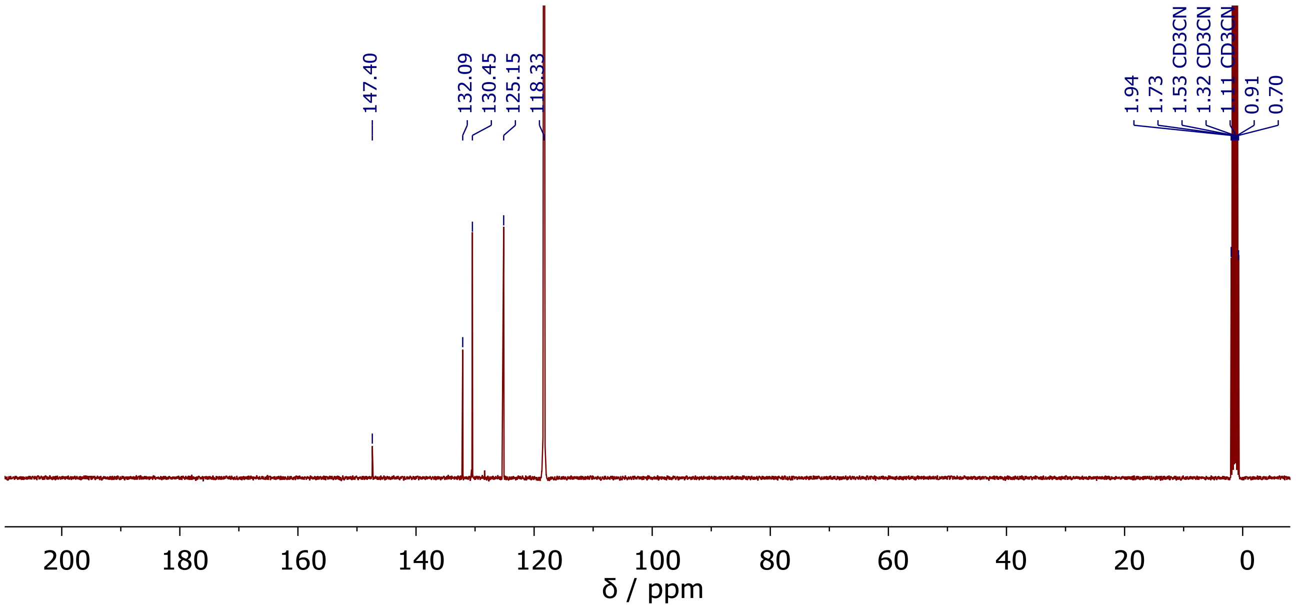

Figure S63. ^{13}C NMR (101 MHz, acetonitrile-d_3, 298 K) spectrum of diphenyl sulfoxide (**4i**).

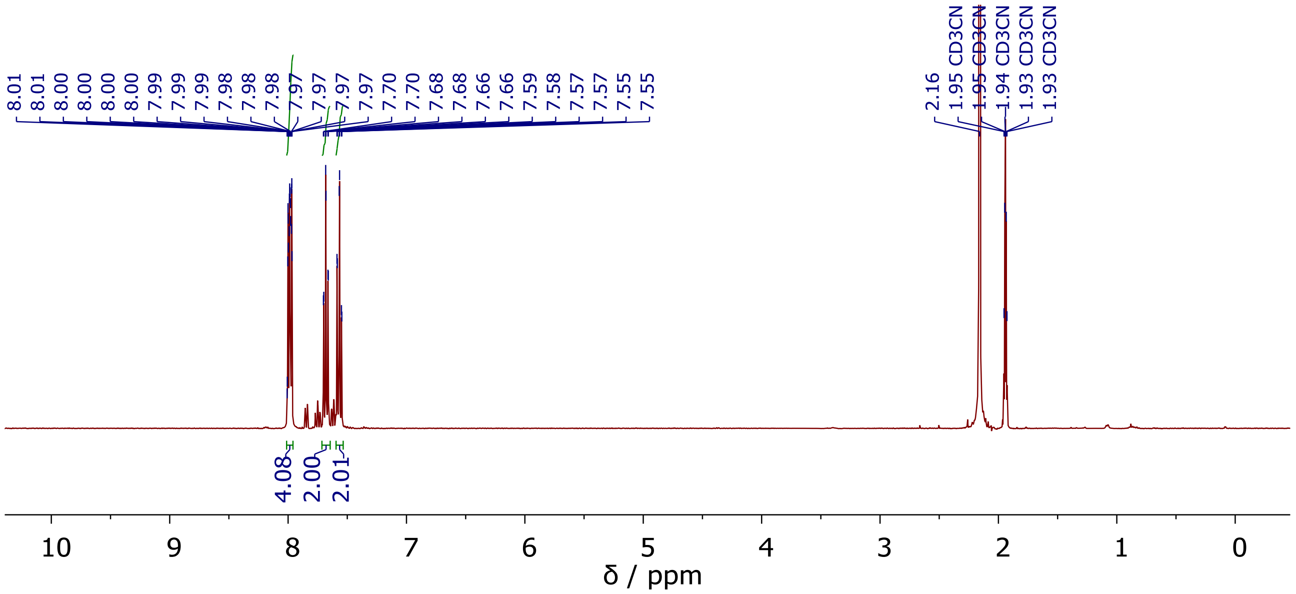

Figure S64. ^{1}H NMR (400 MHz, acetonitrile-d_3, 298 K) spectrum of dibenzo[*b*,*d*]thiophene 5-oxide (**4j**).

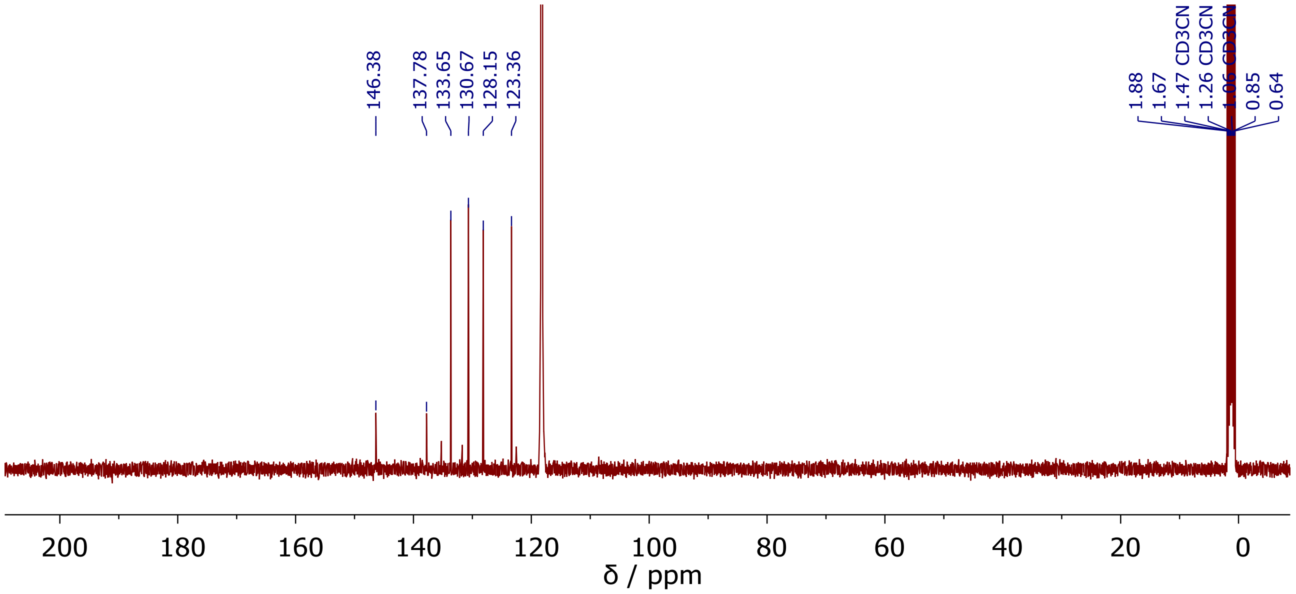

Figure S65. ^{13}C NMR (101 MHz, acetonitrile-d_3, 298 K) spectrum of dibenzo[*b*,*d*]thiophene 5-oxide (**4j**).

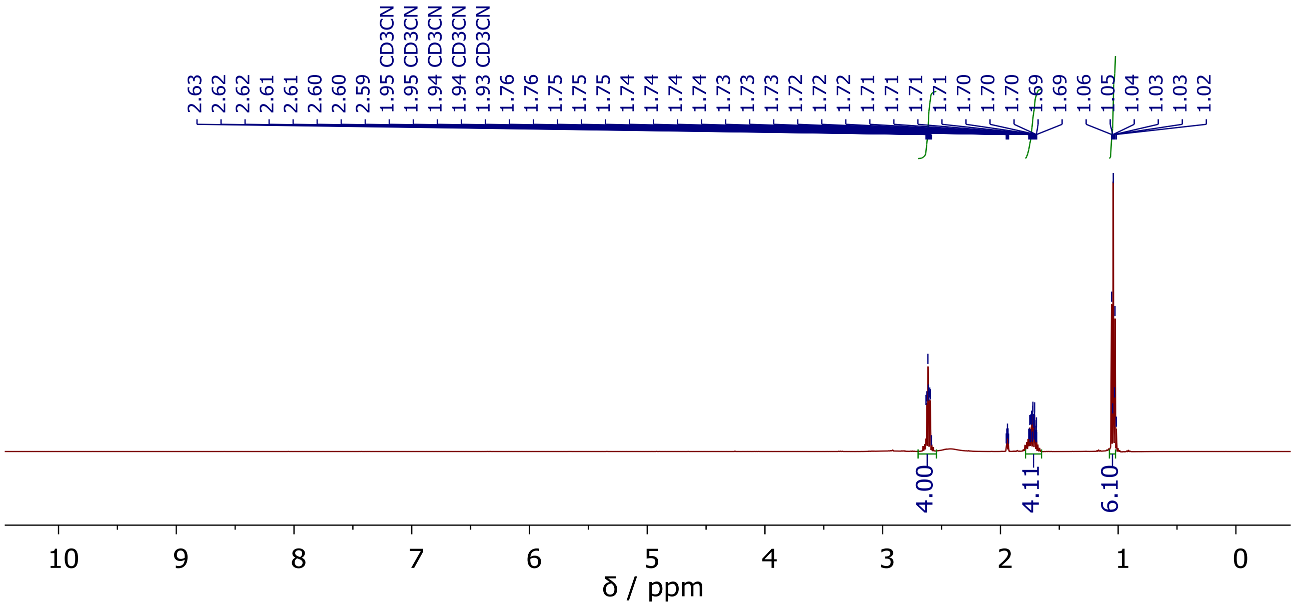

Figure S66. ^{1}H NMR (500 MHz, acetonitrile-d_3, 298 K) spectrum of 1-(propylsulfinyl)propane (**4l**).

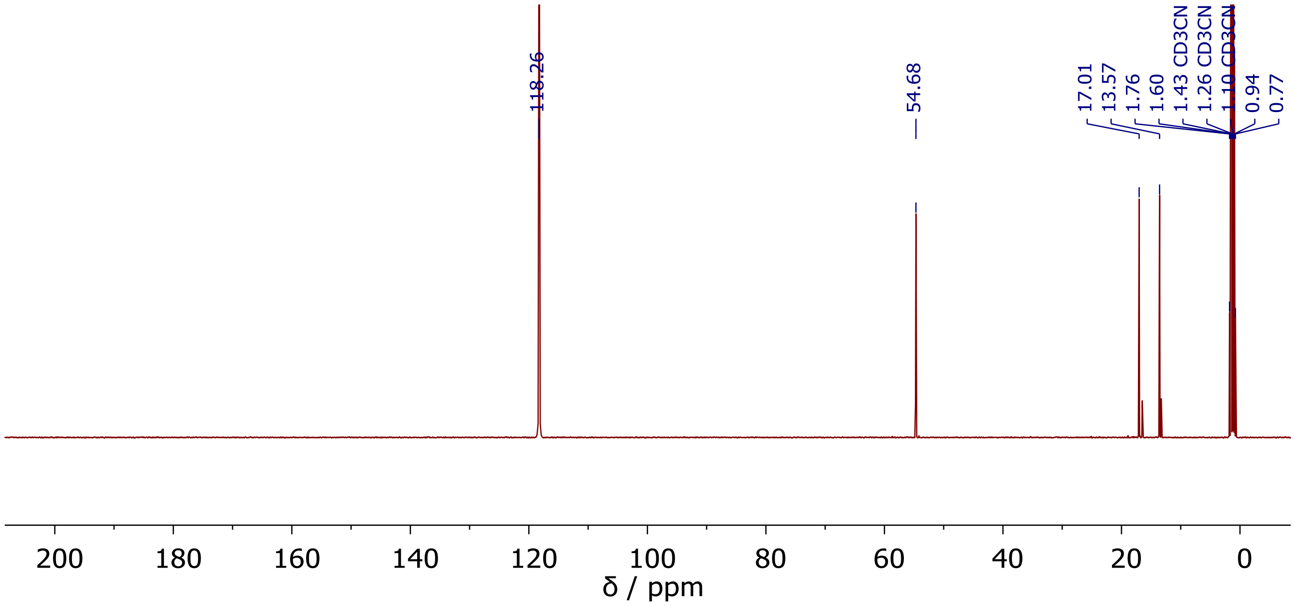

Figure S67. ^{13}C NMR (126 MHz, acetonitrile-d_3, 298 K) spectrum of 1-(propylsulfinyl)propane (**4l**).

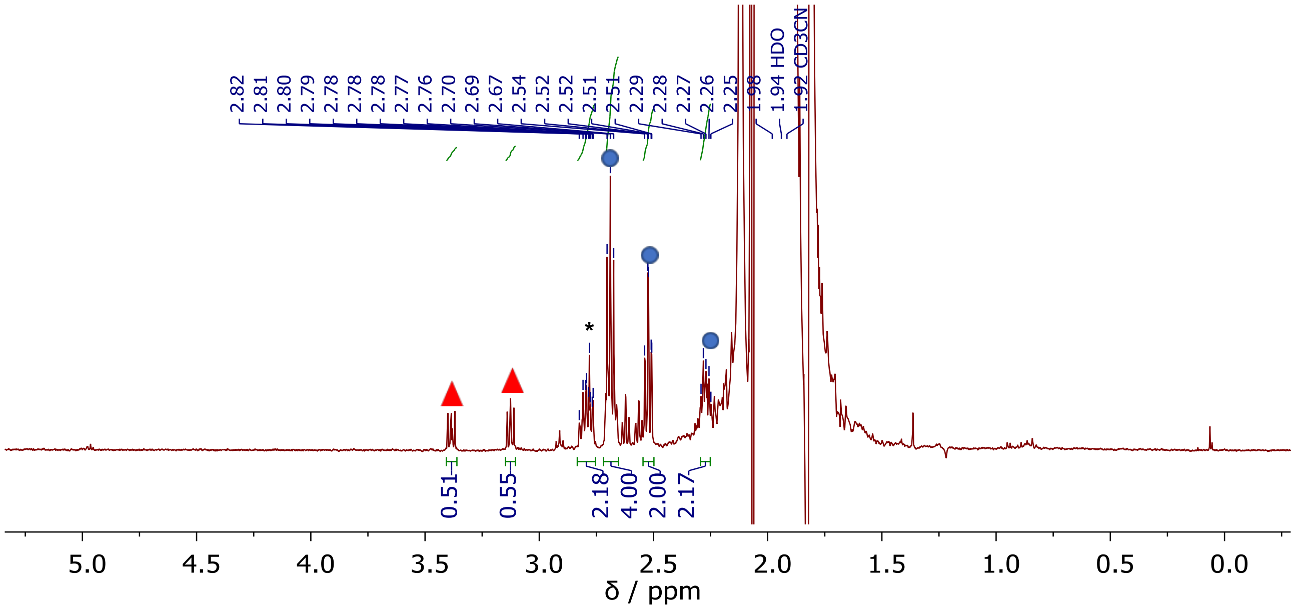

Figure S68. ^{1}H NMR (500 MHz, acetonitrile-d_3, 298 K) spectrum of tetrahydrothiophene 1-oxide (**4n**). Blue circles (•) correspond to sulfoxide **4n**, red triangles (▲) correspond to sulfone **5n**, and * corresponds to the substrate **3a**.

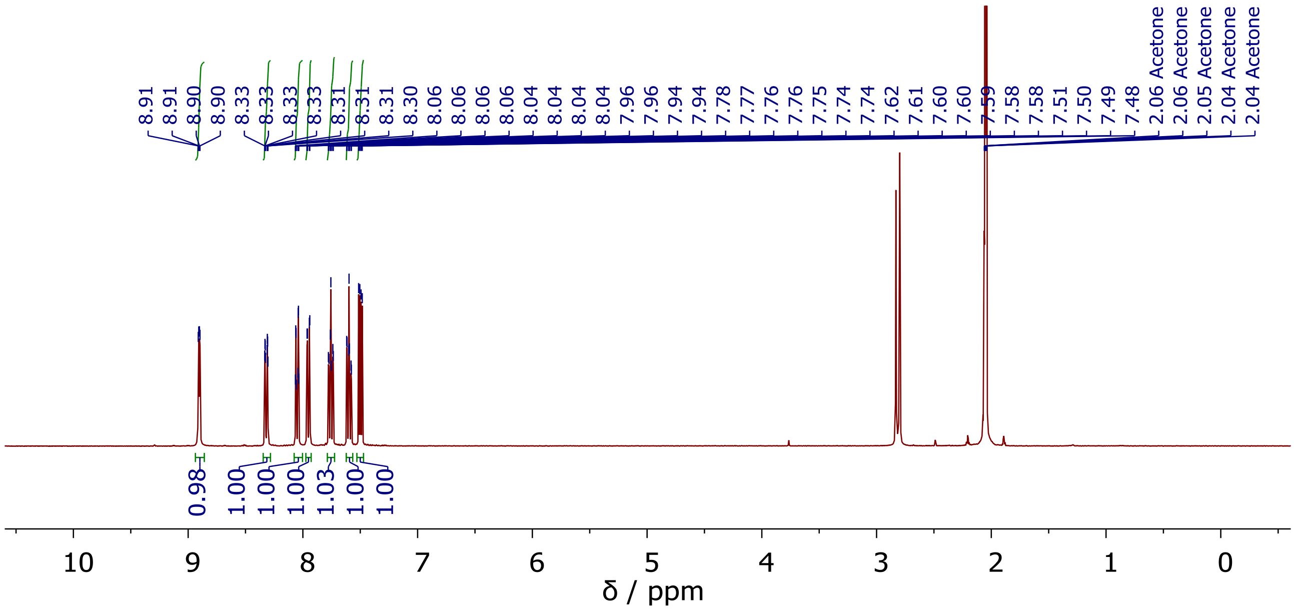

Figure S69. ^{1}H NMR (400 MHz, acetone-d_6, 298 K) spectrum of quinoline (**8a**).

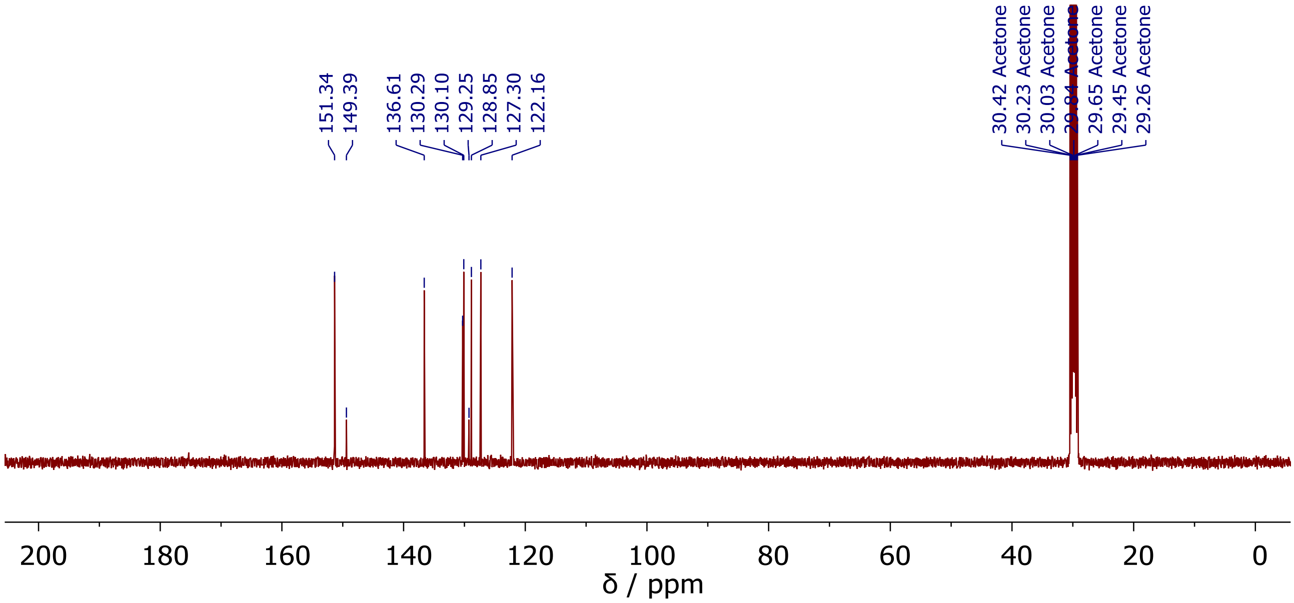

Figure S70. ^{13}C NMR (101 MHz, acetone-d_6, 298 K) spectrum of quinoline (**8a**).

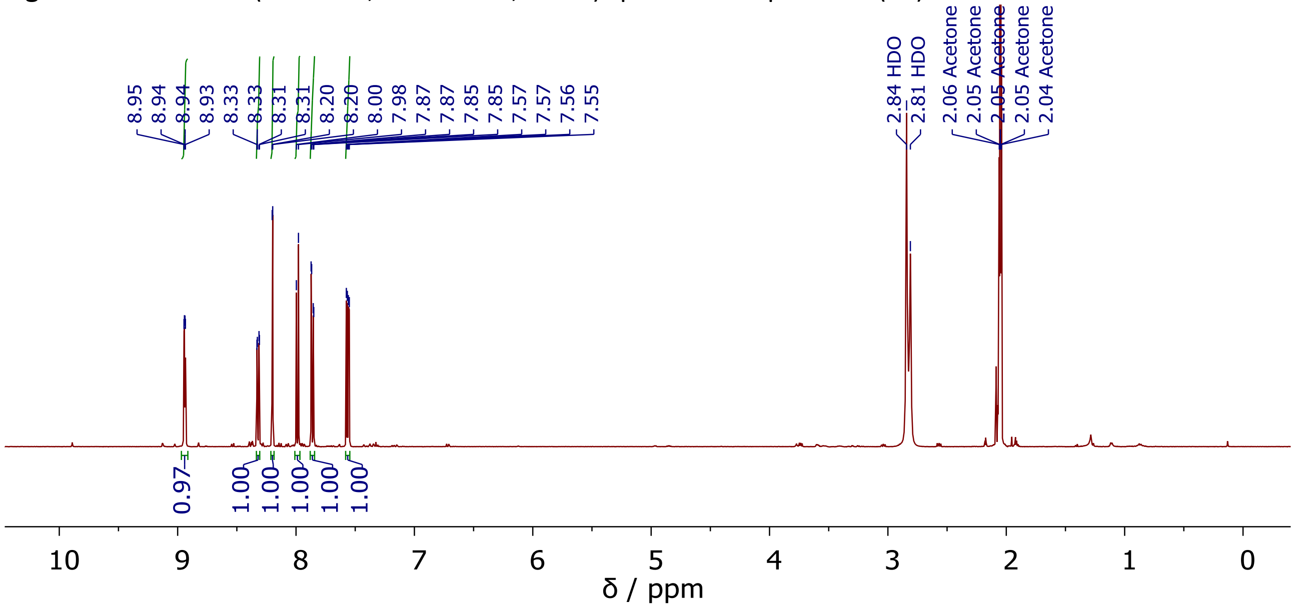

Figure S71. ^{1}H NMR (500 MHz, acetone-d_6, 298 K) spectrum of 6-bromoquinoline (**8b**).

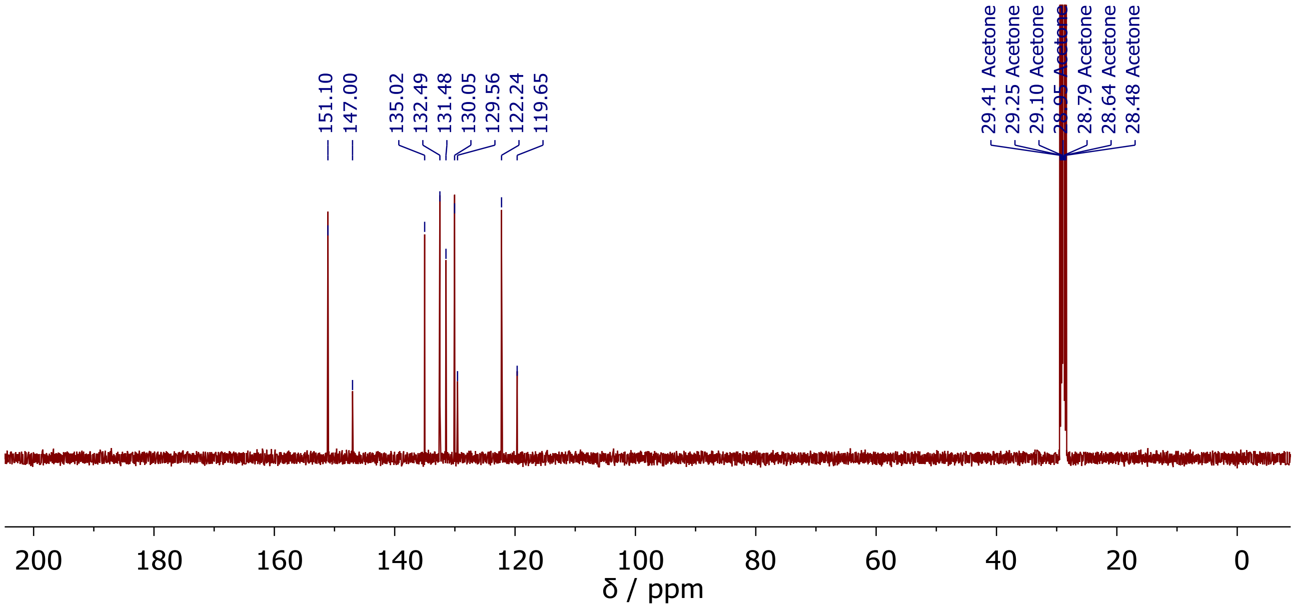

Figure S72. ^{13}C NMR (126 MHz, acetone-d_6, 298 K) spectrum of 6-bromoquinoline (**8b**).

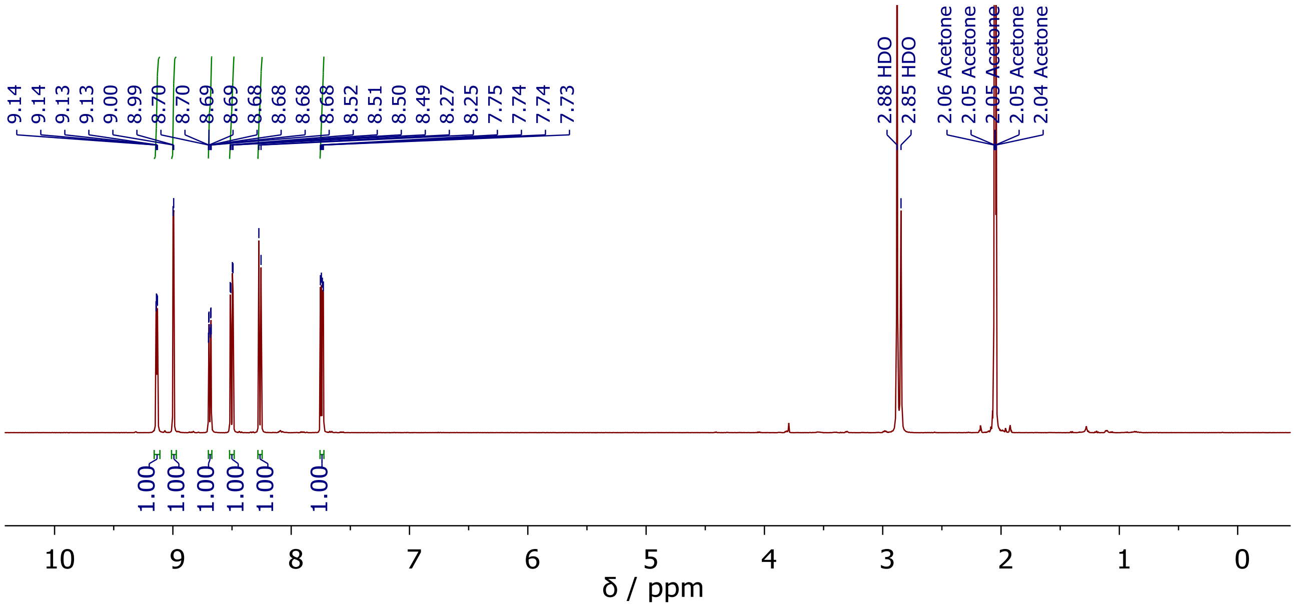

Figure S73. ^{1}H NMR (500 MHz, acetone-d_6, 298 K) spectrum of 6-nitroquinoline (**8c**).

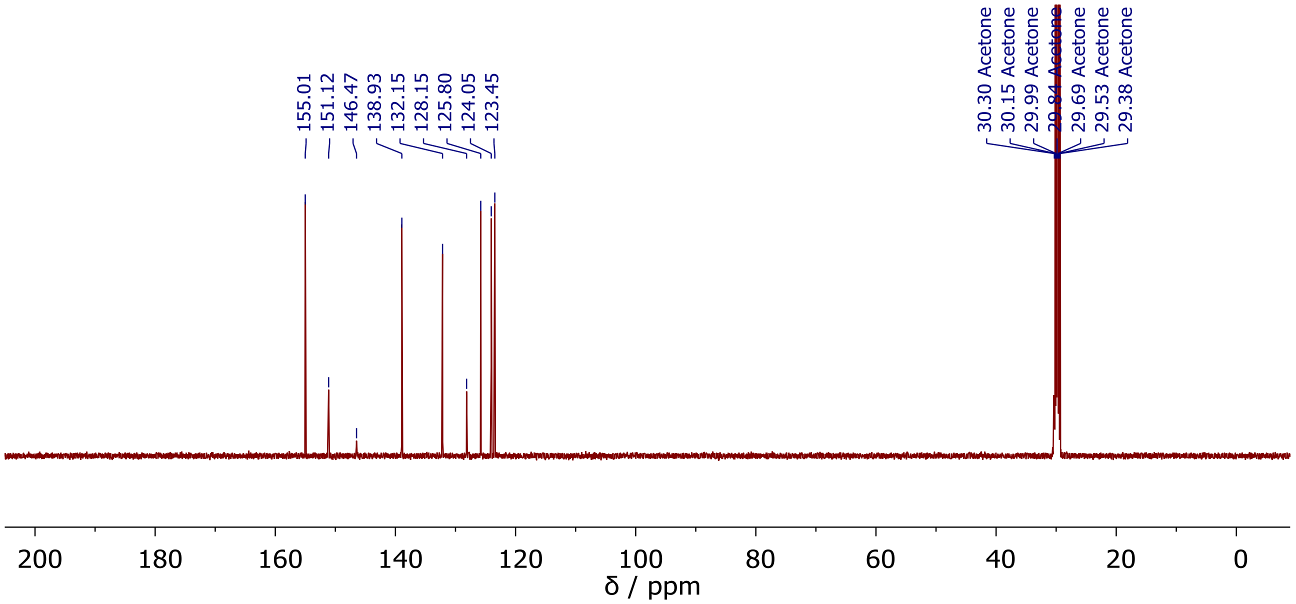

Figure S74. ^{13}C NMR (126 MHz, acetone-d_6, 298 K) spectrum of 6-nitroquinoline (**8c**).

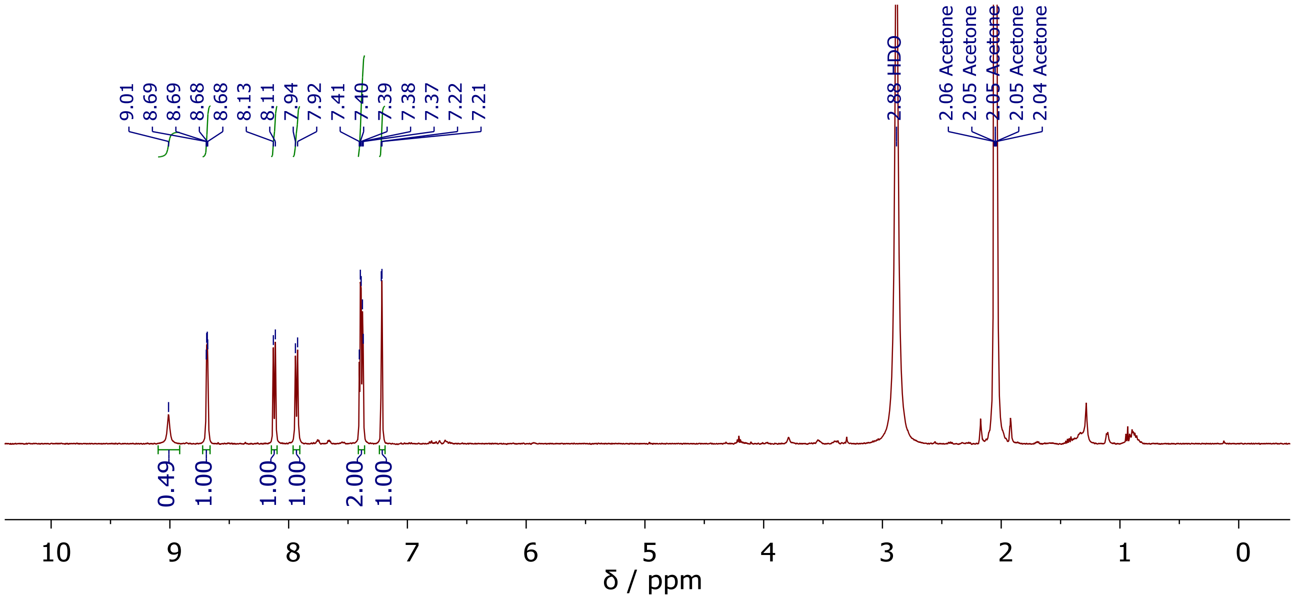

Figure S75. ^{1}H NMR (500 MHz, acetone-d_6, 298 K) spectrum of 6-hydroxyquinoline (**8d**).

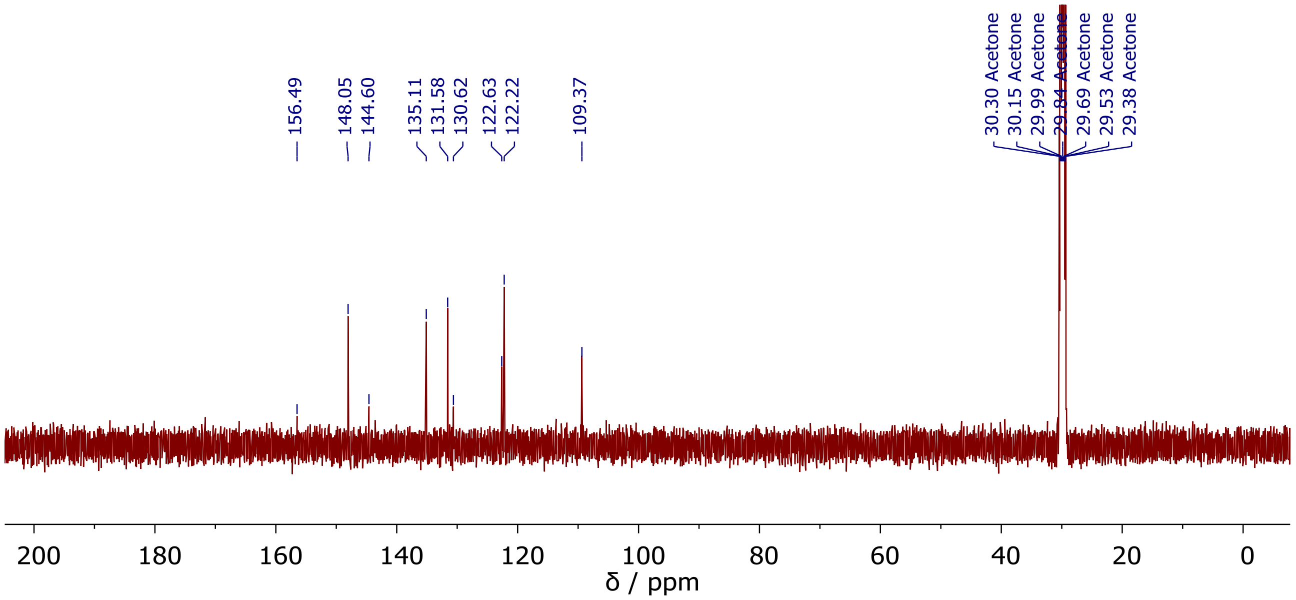

Figure S76. ^{13}C NMR (126 MHz, acetone-d_6, 298 K) spectrum of 6-hydroxyquinoline (**8d**).

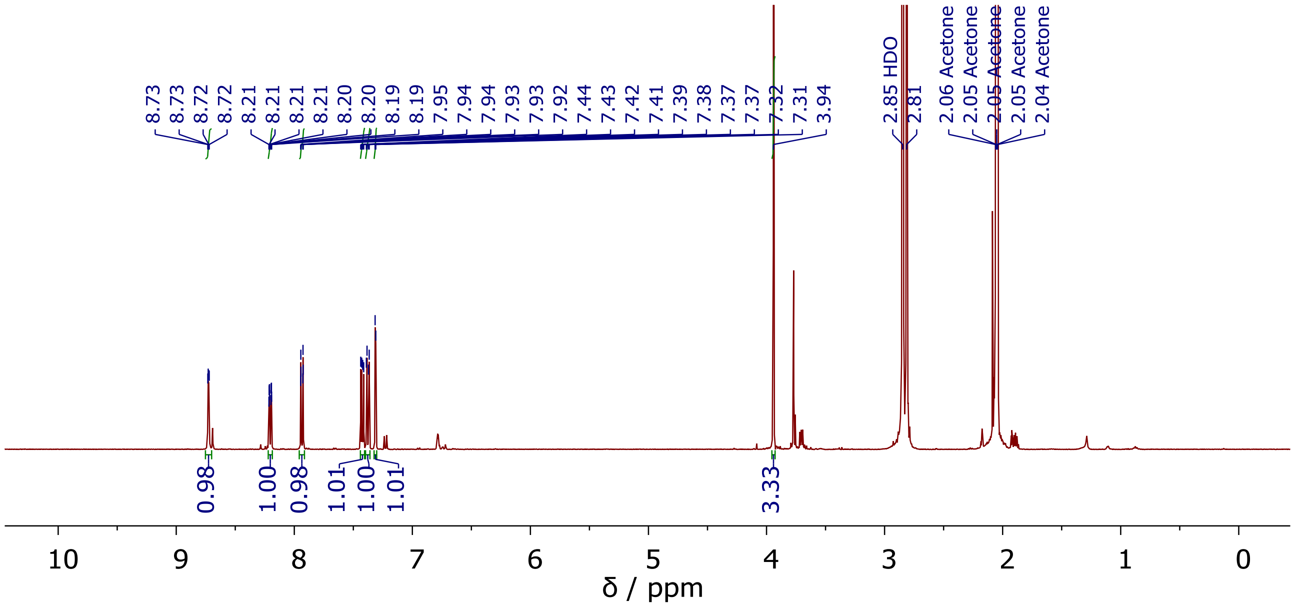

Figure S77. ^{1}H NMR (500 MHz, acetone-d_6, 298 K) spectrum of 6-methoxyquinoline (**8e**).

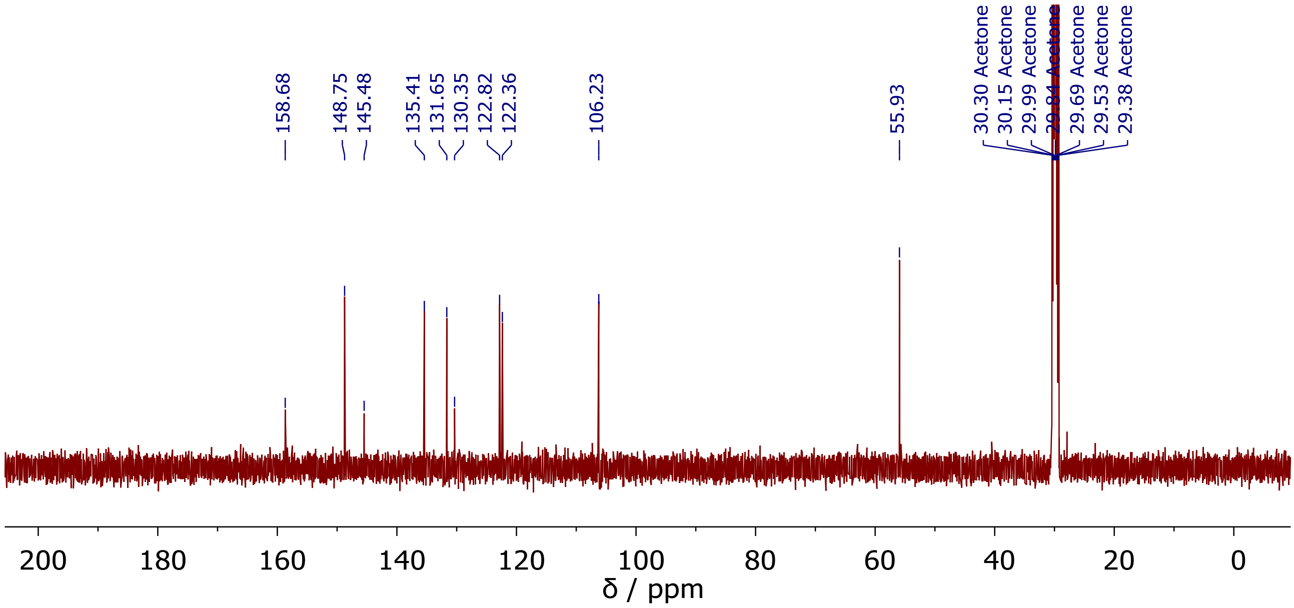

Figure S78. ^{13}C NMR (126 MHz, acetone-d_6, 298 K) spectrum of 6-methoxyquinoline (**8e**).

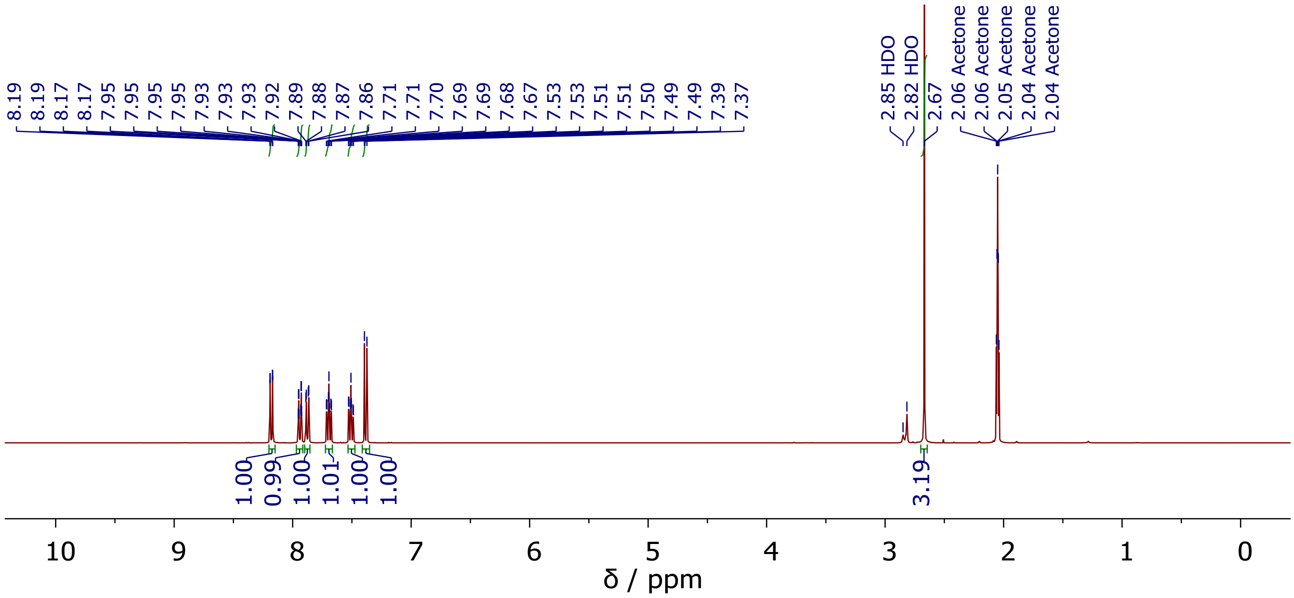

Figure S79. ^{1}H NMR (400 MHz, acetone-d_6, 298 K) spectrum of 2-methylquinoline (**8f**).

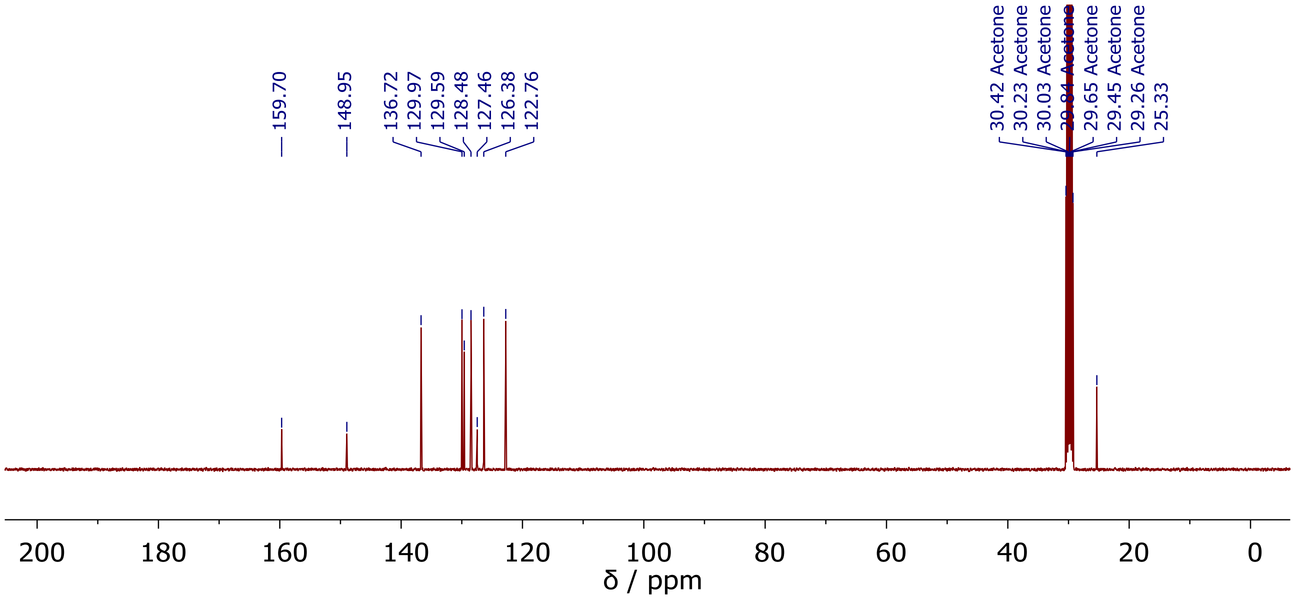

Figure S80. ^{13}C NMR (101 MHz, acetone-d_6, 298 K) spectrum of 2-methylquinoline (**8f**).

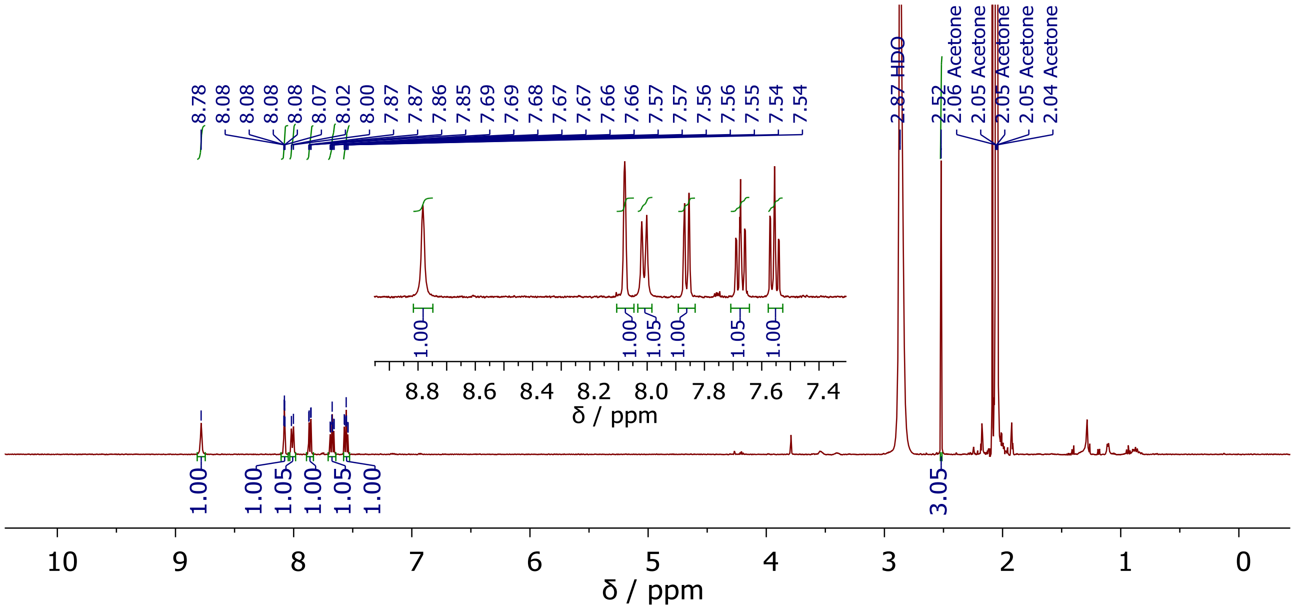

Figure S81. ^{1}H NMR (500 MHz, acetone-d_6, 298 K) spectrum of 3-methylquinoline (**8g**).

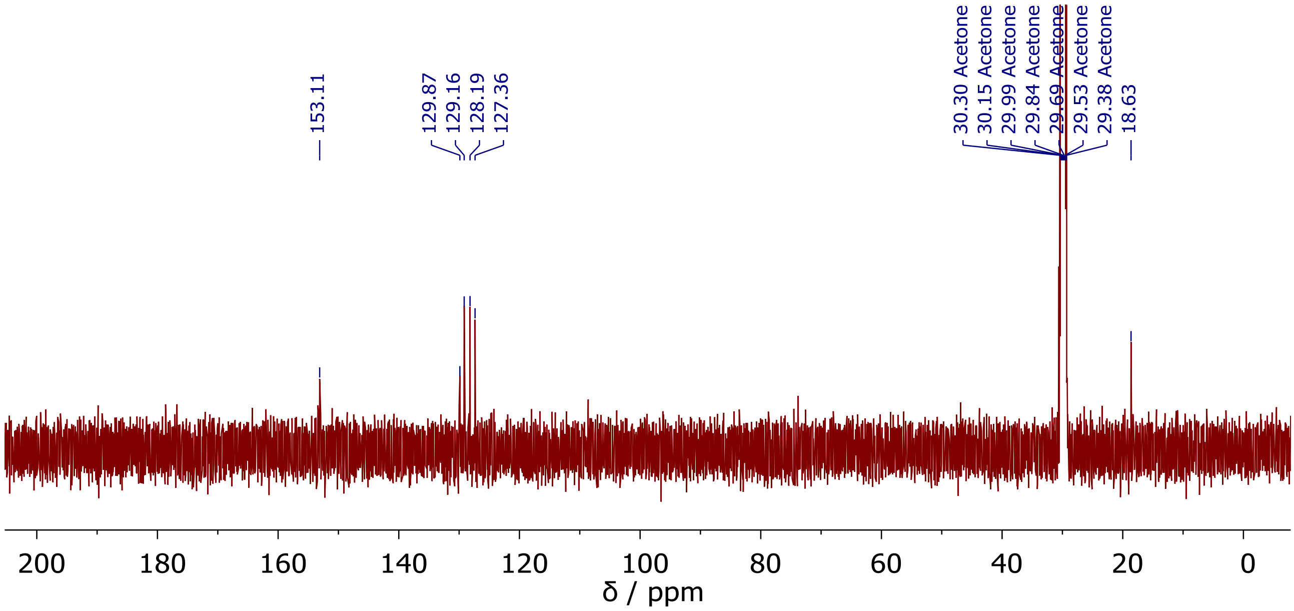

Figure S82. ^{13}C NMR (126 MHz, acetone-d_6, 298 K) spectrum of 3-methylquinoline (**8g**).

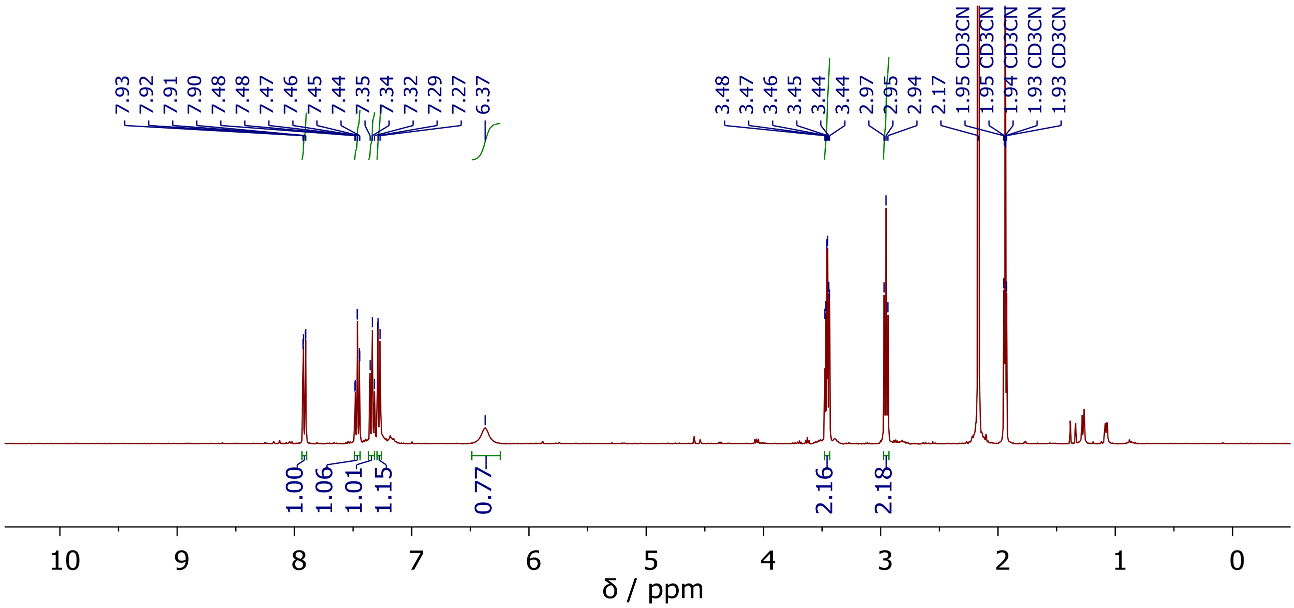

Figure S83. ^{1}H NMR (400 MHz, acetonitrile-d_3, 298 K) spectrum of 3,4-dihydroisoquinoline (**7h**).

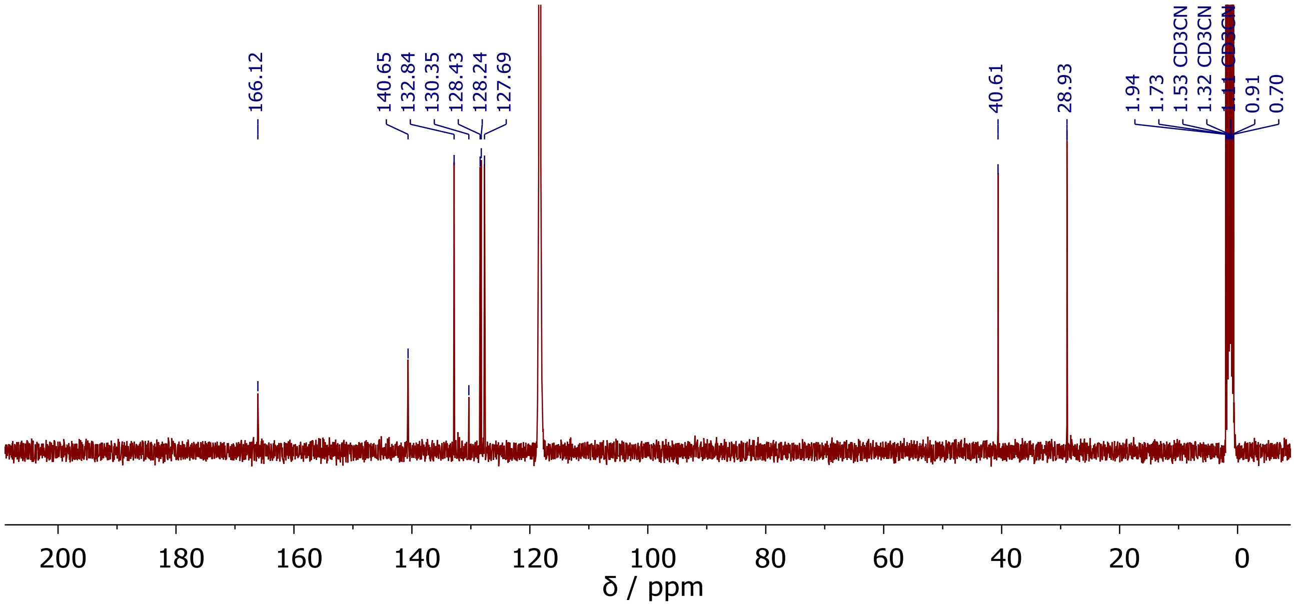

Figure S84. ^{13}C NMR (101 MHz, acetonitrile-d_3, 298 K) spectrum of 3,4-dihydroisoquinoline (**7h**).

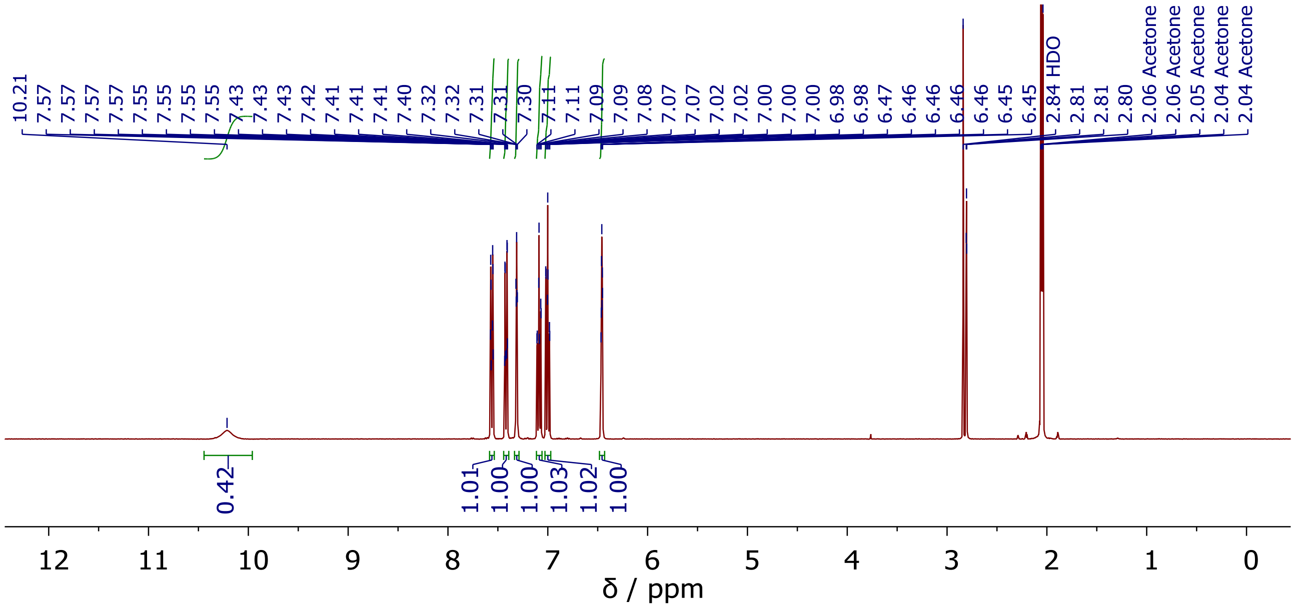

Figure S85. ^{1}H NMR (400 MHz, acetone-d_6, 298 K) spectrum of indole (**7i**).

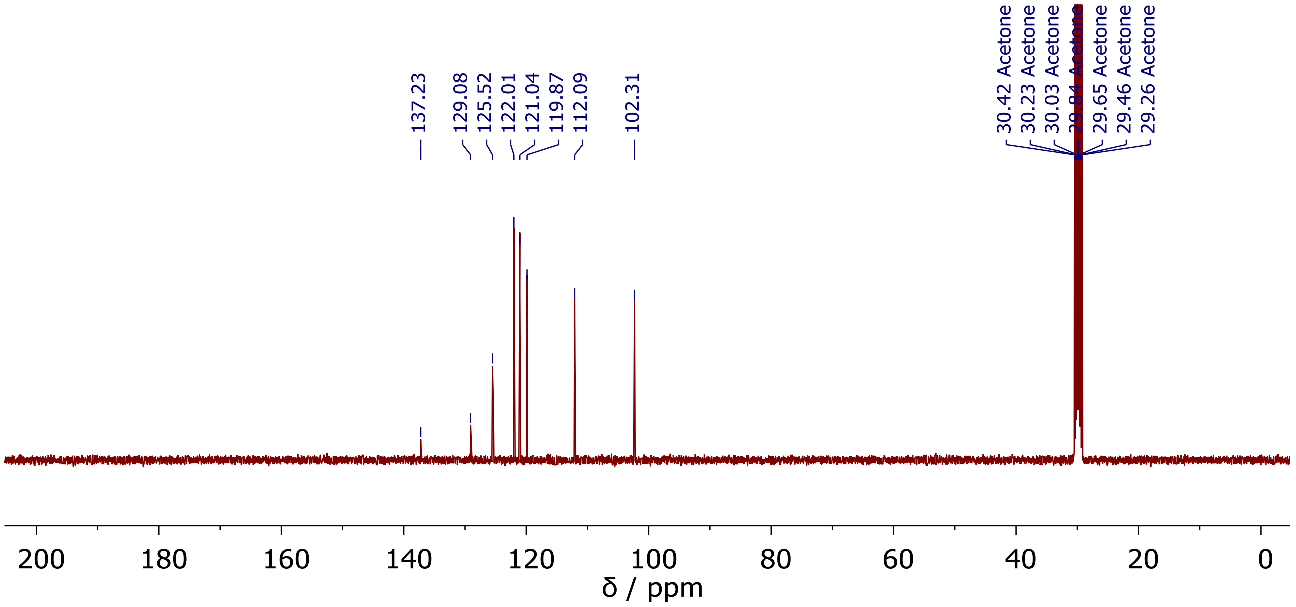

Figure S86. ^{13}C NMR (101 MHz, acetone-d_6, 298 K) spectrum of indole (**7i**).

REFERENCES

(1) Stoll, S.; Schweiger, A. EasySpin, a Comprehensive Software Package for Spectral Simulation and Analysis in EPR. *Journal of Magnetic Resonance* **2006**, *178* (1), 42–55. https://doi.org/10.1016/j.jmr.2005.08.013.

(2) Materna, K. L.; Hammarström, L. Photoredox Catalysis Using Heterogenized Iridium Complexes. March 2, 2021. https://doi.org/10.26434/chemrxiv.14132213.v1.

(3) Albert Weller. Photoinduced Electron Transfer in Solution: Exciplex and Radical Ion Pair Formation Free Enthalpies and Their Solvent Dependence. *Zeitschrift für Physikalische Chemie* **1982**, *133*, 93–98.

(4) Taras-Goslinska, K.; Jonsson, M. Solvent Effects on the Redox Properties of Thioethers. *Journal of Physical Chemistry A* **2006**, *110* (30), 9513–9517. https://doi.org/10.1021/jp0623746.
